# Antimutator and Mutational Spectrum Effects Can Combine to Reduce Evolutionary Potential in *Escherichia coli* Δ*nudJ*

**DOI:** 10.1093/molbev/msaf182

**Published:** 2025-07-29

**Authors:** Rowan Green, Huw Richards, Deniz Ozbilek, Francesca Tyrrell, Victoria Barton, Ziang Zhang, Simon C Lovell, Danna R Gifford, Mato Lagator, Andrew J McBain, Rok Krašovec, Christopher G Knight

**Affiliations:** School of Natural Sciences, Faculty of Science and Engineering, University of Manchester, Manchester, UK; Department of Zoology, Faculty of Science, University of British Columbia, Vancouver, Canada; School of Natural Sciences, Faculty of Science and Engineering, University of Manchester, Manchester, UK; School of Biological Sciences, Faculty of Biology, Medicine & Health, University of Manchester, Manchester, UK; School of Biological Sciences, Faculty of Biology, Medicine & Health, University of Manchester, Manchester, UK; School of Biological Sciences, Faculty of Biology, Medicine & Health, University of Manchester, Manchester, UK; School of Biological Sciences, Faculty of Biology, Medicine & Health, University of Manchester, Manchester, UK; School of Biological Sciences, Faculty of Biology, Medicine & Health, University of Manchester, Manchester, UK; School of Biological Sciences, Faculty of Biology, Medicine & Health, University of Manchester, Manchester, UK; School of Biological Sciences, Faculty of Biology, Medicine & Health, University of Manchester, Manchester, UK; School of Health Sciences, Faculty of Biology, Medicine & Health, University of Manchester, Manchester, UK; School of Biological Sciences, Faculty of Biology, Medicine & Health, University of Manchester, Manchester, UK; School of Natural Sciences, Faculty of Science and Engineering, University of Manchester, Manchester, UK

**Keywords:** mutation rate, distribution of fitness effects, mutation rate plasticity, density associated mutation rate plasticity, Nudix, NudJ, mutational spectrum, mutational signature, anti-evolution drugs, RpoB, rifampicin

## Abstract

The rate of spontaneous mutation is a key factor in determining the capacity of a population to adapt to a novel environment, for example, a bacterial population exposed to antibiotics. Genetic and environmental factors controlling the mutation rate commonly also cause shifts in the relative rates of different mutational classes, i.e. the mutational spectrum. When the mutational spectrum is altered, the relatively enriched and depleted mutations may differ in their fitness effects. Here, we explore how a reduced mutation rate and altered mutational spectrum can contribute to adaptation in *Escherichia coli*. We measure mutation rates across a set of Nudix hydrolase deletants, finding multiple strains with an antimutator phenotype. We focus on the antimutator Δ*nudJ*, which can cause a 6-fold mutation rate reduction relative to the wildtype, with an altered mutational spectrum biased towards A > C transversions. Its reduced mutation rate, most pronounced at low population densities, appears to occur via NudJ's role in nucleotide and/or prenyl metabolism, with a reduced internal ATP pool. Its effects may be reversed by mutations to genes, including *waaZ*, affecting the outer membrane. Not only does *nudJ* deletion reduce the probability of antibiotic resistance arising at all but through enhancing an existing hotspot for low fitness A > C rifampicin resistance mutations reduces the expected fitness of strains when resistance does arise. Thus, our findings with Δ*nudJ* suggest future anti-evolution drug strategies could suppress spontaneous resistance evolution not only through minimizing resistance mutations but also by specifically limiting access to the fittest mutations.

## Introduction

Understanding what controls microbial mutation rates is a central question in microbial evolution with important applications for tackling antimicrobial resistance and improving the reliability of engineered microbes. Rapid adaptation by “mutator” strains, with elevated mutation rates, can allow bacterial populations to overcome single (Couce et al. 2015), sequential (Elgrail et al. 2024), and double (Gifford et al. 2023) antibiotic therapy. Conversely “antimutator” strains, with reduced mutation rates, are less prone to developing antimicrobial resistance (Ragheb et al. 2019) and can provide more stable engineered strains for synthetic biology applications (e.g. [Pośfai et al. 2006; Umenhoffer et al. 2010; Csörgő et al. 2012; Deatherage et al. 2018; ScarabGenomics 2024]). Identifying ways to reduce bacterial mutation rates is therefore an area of great interest.

Increased mutation rates, caused by the disruption of genes involved in DNA replication and repair, are observed relatively frequently in both laboratory grown bacteria (e.g. [Good et al. 2017; Callens et al. 2023]) and clinical infections (e.g. [Oliver et al. 2000; Couce et al. 2016]). In contrast, strains with genetic reduction of mutation rates, termed “antimutator” strains, are more rarely observed, particularly when the genetic change is gene inactivation. Here, we identify genes that normally increase mutation rate, whose disruption gives an antimutator phenotype. Given the importance of mutation rates in determining adaptation to new environments (Swings et al. 2017), such genes could provide useful targets for “anti-evolution” drugs (Cirz et al. 2005; Alam et al. 2016; Ragheb et al. 2019; Carvajal-Garcia et al. 2024).

Most previous research into antimutators focuses on secondary mutations that reverse a preexisting mutator phenotype towards the wildtype phenotype (e.g. [Fijalkowska et al. 1993; Schaaper 1993, 1996; Wielgoss et al. 2013; Swings et al. 2017; Sprouffske et al. 2018; Ho et al. 2021]). Though rare, antimutator alleles in a wildtype background have been identified: *mfd* knockout in multiple bacterial species (Ragheb et al. 2019); mutants of *dnaE* in *Escherichia coli* (Oller and Schaaper 1994); various mutations of DNA polymerase (gene 43) of phage T4 (Drake 1993); and a temperature sensitive mutant of *purB* in *E. coli* (Geiger and Speyer 1977; but see Schaaper and Dunn 2001). These genes are integral to DNA replication and repair pathways, indicating that other components of these pathways might also have the potential to act as antimutators, although they have been less frequently identified or studied.

One under-explored gene family in this context encodes the Nudix hydrolase proteins. The Nudix hydrolases were originally defined by their “house-cleaning” role in degrading potentially deleterious metabolites (Bessman et al. 1996). Specifically, they were characterized by their activity in hydrolyzing nucleoside diphosphate derivatives, though substrates outside of this group have also been identified (Bessman et al. 1996, 2019; McLennan 2006). The only enzyme from this family to have been well studied in relation to mutational dynamics is MutT (also known as NudA). The Nudix knockout Δ*mutT* produces a mutator phenotype in bacteria (Maki and Sekiguchi 1992), yeast (homolog *PCD1*) (Nunoshiba 2004; Krašovec et al. 2017), and human cancer (homolog *MTH1*) (Tsuzuki et al. 2001). As well as increasing mutation rates, Δ*mutT* has been shown to alter the mutational spectrum via an increase in the relative rate of A > C transversions (Tajiri et al. 1995). MutT is also involved in density associated mutation rate plasticity, the widely conserved negative association between microbial mutation rates and final population density in batch culture (Krašovec et al. 2017). While wildtype strains of *E. coli* and *S. cerevisiae* show the expected association between population density and mutation rate, deletants of *mutT* and *PCD1,* respectively, show a constant, high, mutation rate across a range of final population densities (Krašovec et al. 2017, 2018). The role of MutT in density-associated mutation rate plasticity (DAMP) underscores its importance in controlling mutation rate dynamics. In contrast to *mutT*, little is known about the mutational consequences of knocking out other members of the Nudix hydrolase family (Kamiya et al. 2003; Hori et al. 2006; McLennan 2013 ).

We begin this study by examining mutation rates across a set of Nudix hydrolase knockouts in *E. coli*. We find that 4 of the 10 knockouts show a significant antimutator phenotype, indicating that their gene products contribute to mutagenesis. We characterize the mutational spectrum of one of these newly identified antimutators, Δ*nudJ,* identifying a significant shift towards A > C transversions. Interestingly, unlike the mutator Δ*mutT*, which also increases the proportion of A > C transversions, the Δ*nudJ* antimutator achieves this shift by decreasing the rate of A > G transitions. Following recent work in this area (Couce et al. 2013; Cano et al. 2022; Sane et al. 2023; Tuffaha et al. 2023), we explore whether the altered mutational spectrum of Δ*nudJ* results in differing fitness outcomes following selection for resistance to the antibiotic rifampicin. We find that the A > C dominated spectrum of Δ*nudJ* results in a bias towards less fit mutations. Therefore Δ*nudJ* adaptation to rifampicin is impaired both by a reduction in the total mutation rate and a bias towards high cost mutations.

## Results

### Several Nudix Hydrolase Knockouts Show an Antimutator Phenotype

**Fig. 1.**
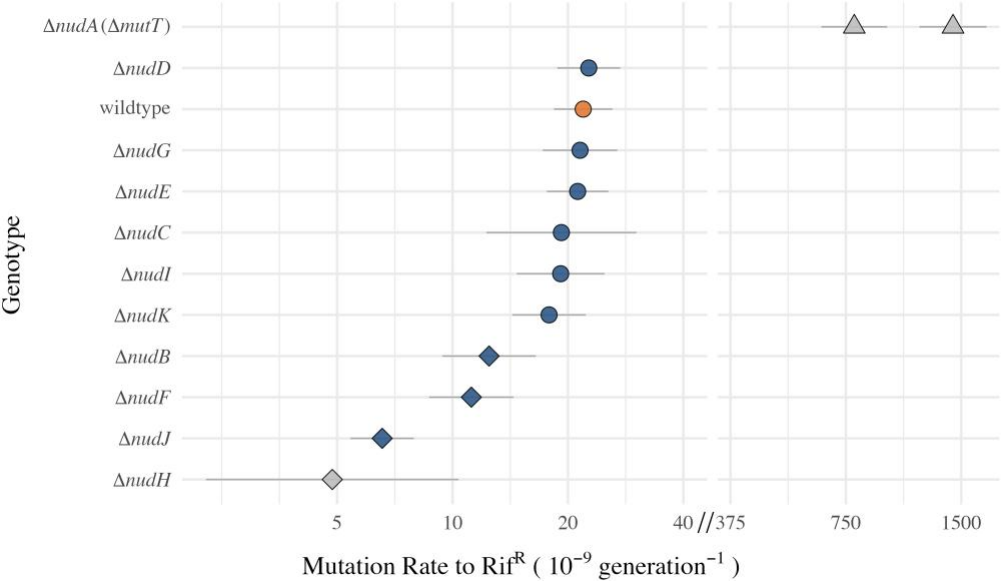
Mutation rate in nudix hydrolase knockouts. Points show mutation rate per genome per generation and error bars show 95% confidence intervals. Diamonds indicate strains with a significantly lower mutation rate than the wildtype BW25113 see supplementary table S1, Supplementary Material online. The wildtype BW25113 is highlighted in orange. Δ*nudH* is shown in grey as this point is an extrapolation (see text). Each of these estimates and error, aside from Δ*nudA,* derives from Statistical Model 1 fitted to multiple individual mutation rate estimates, collected at different population densities and accounting for observed variation of rate with density (see Supplementary Statistical Methods). Mutation rates shown here are estimated at a consistent population density of 1.47 × 10^8^·CFU.mL^−1^. Both the underlying data and fitted model are shown in supplementary fig. S1, Supplementary Material online. The fold difference between the mutation rate of Δ*nudA* and wildtype *E. coli* to nalidixic acid were previously measured by (Krašovec et al. 2017), using these data, the mutation rates of Δ*nudA* to rifampicin resistance were estimated. Both Keio knockout strains (Baba et al. 2006) are shown for Δ*nudA.* Note log scale *x* axis and break between 40 and 375. Genotypes shown from bottom to top are Δ*nudH* (*N*_Fluctuation Assays_ =12, *N*_Parallel Cultures_ =195), Δ*nudJ* (*N*_FA_ =47, *N*_PC_ =1089), Δ*nudF* (*N*_FA_ =16, *N*_PC_ =259), Δ*nudB* (*N*_FA_ =14, *N*_PC_ =228), Δ*nudK* (*N*_FA_ =14, *N*_PC_ =226), Δ*nudC* (*N*_FA_ =12, *N*_PC_ =193), Δ*nudI* (*N*_FA_ =14, *N*_PC_ =225), Δ*nudG* (*N*_FA_ =14, *N*_PC_ =226), Δ*nudE* (*N*_FA_ =14, *N*_PC_ =228), wildtype BW25113 (*N*_FA_ =92, *N*_PC_ =1613), Δ*nudD* (*N*_FA_ =14, *N*_PC_ =227), Δ*nudA*1 (estimated rifR mutation rate = 1428 × 10^−9^ gen^−1^, *N*_FA_ =30) & Δ*nudA*2 (estimated rifR mutation rate = 789 × 10^−9^ gen^−1^, *N*_FA_ =33). Raw data can be found in data file S3.

The Nudix hydrolase MutT plays a vital role in maintaining the wildtype mutation rate in *E. coli* (Maki and Sekiguchi 1992). However, *E. coli* contains a further 13 proteins with a Nudix domain, whose effects on mutation rate have, in most cases, not been reported (though see Kamiya et al. 2003; Hori et al. 2006). We estimated mutation rates across single-gene deletants of a further ten Nudix hydrolases to systematically assess the roles of this enzyme family in mutagenesis (Fig. 1). The magnitude of mutation rate change in Δ*mutT* strains (estimated to be between 50-fold (Swings et al. 2017) and 139-fold (Sprouffske et al. 2018) higher than the wildtype) enabled its early discovery as a mutator (Treffers et al. 1954). In contrast, the Nudix knockouts surveyed here show much more subtle effects on mutation rate; none show significant increases in mutation rate, but 4 of the 10 tested (Δ*nudB*, Δ*nudF*, Δ*nudH* and Δ*nudJ*) show significant reductions in mutation rate, between 1.8- and 4.5-fold lower than the wildtype (Fig. 1, supplementary table S1, Supplementary Material online). It is important to note that this estimate of spontaneous mutation rate is based on rifampicin resistance which is due to single nucleotide polymorphisms (SNPs) and small indels; it does not consider larger indels, local copy number alterations, or chromosomal rearrangements (Garibyan 2003).

The largest fold change in mutation rate shown is in Δ*nudH.* However, a growth defect in this strain prevents it from reaching comparable density to the other strains. Given the known effect of population density on mutation rates (Krašovec et al. 2017; Green et al. 2024), the mutation rate estimates shown in Fig. 1 are at the same population density across all assays—the overall mean. The Δ*nudH* strain never achieves this population density in our experiments; therefore, the mutation rate shown in Fig. 1 is an extrapolation (see supplementary fig. S1, Supplementary Material online), giving the estimate large error bars and questionable value. After Δ*nudH*, the largest change is seen in Δ*nudJ*, which is a clear outlier within the remaining Nudix deletant strains tested here, showing a mutation rate reduction at this population density of 3.3-fold (Fig. 1). A qualitatively similar reduction in mutation rate is seen for this strain at different marker loci (nalidixic acid resistance at the *gyrA* and *gyrB* locus [Harmand et al. 2017] and D-cycloserine resistance at the *cycA* locus [Maharjan and Ferenci 2015], supplementary fig. S2, Supplementary Material online). We therefore focus primarily on Δ*nudJ* going forward.

### NudJ's Metabolic Contribution to Mutagenesis via Prenyl and Nucleotide Metabolism

**Fig. 2.**
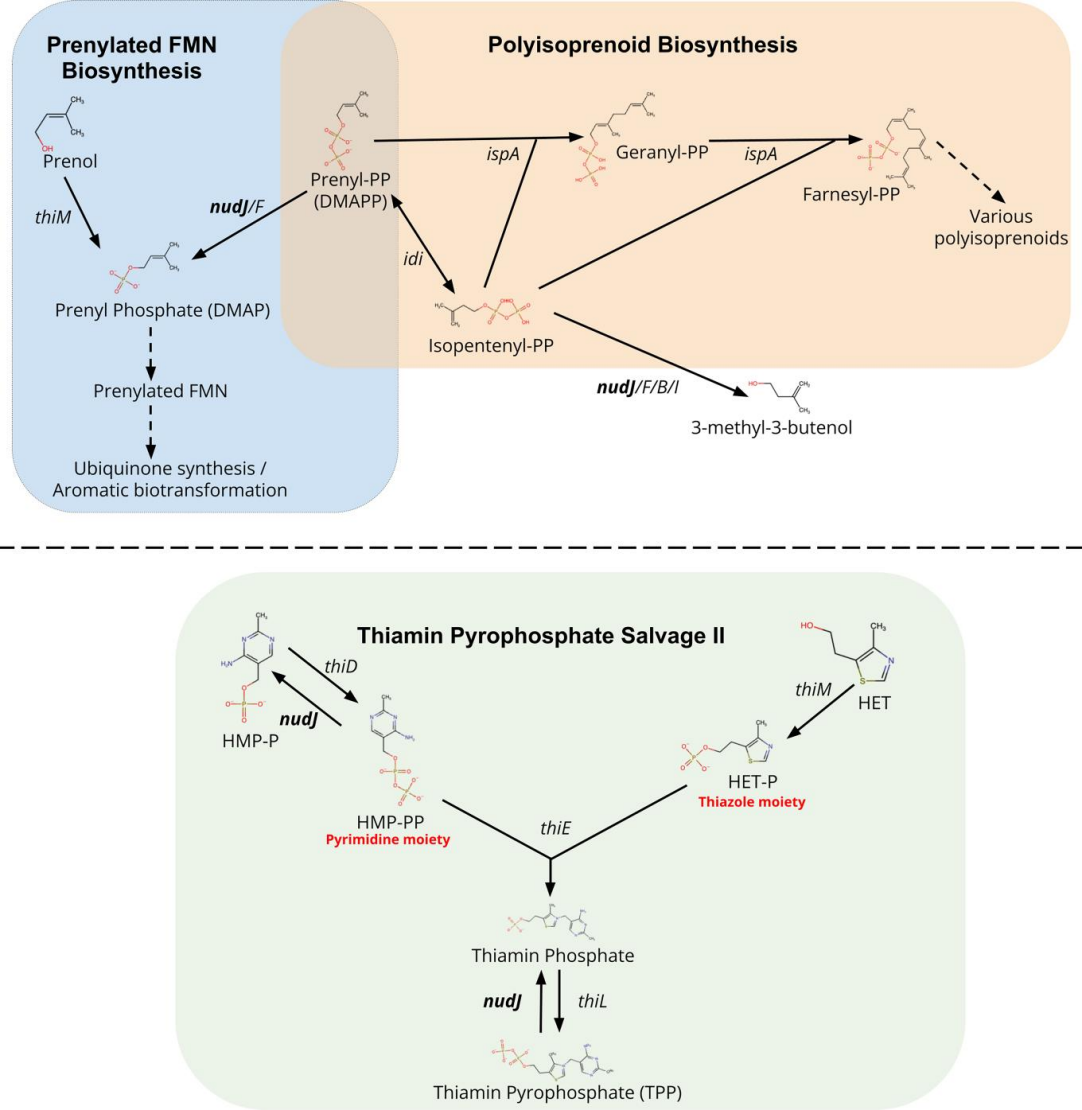
Simplified diagram of the comparative roles of ThiM and NudJ. In the prenylated FMN biosynthesis pathway, both NudJ and ThiM produce DMAP, enabling prenylated FMN production. However, in thiamin pyrophosphate salvage, ThiM contributes positively to TPP production while NudJ antagonises TPP production. The antagonistic roles of NudJ and IspA in polyisoprenoid biosynthesis are also included. See main text for references associated with all reactions shown. All chemical structures were generated using the RCSB PDB Chemical Sketch Tool (https://www.rcsb.org/chemical-sketch).

The observation of a strong antimutator phenotype in Δ*nudJ* indicates that some activity of NudJ is contributing to mutagenesis in *E. coli*. NudJ is known to show strong dephosphorylating activity in vitro against dimethylallyl-diphosphate (DMAPP), producing dimethylallyl-monophosphate (DMAP). DMAP is required for the production of the UbiD cofactor prenylated flavin mononucleotide (prFMN), which is essential for ubiquinone biosynthesis (Gulmezian et al. 2007; Payne et al. 2015; Wang et al. 2018; Fig. 2). DMAPP dephosphorylation activity is seen in 3 of the 4 identified antimutators (NudB, F & J but not NudH) but only in one of the 7 non-antimutators (NudI). As well as dephosphorylating DMAPP, NudJ also dephosphorylates the essential cofactor thiamin phyrophospate (TPP) and its precursor in thiamin salvage, 4-amino-2-methyl-5-hydroxymethylpyrimidine pyrophosphate (HMP-PP), in vitro (Lawhorn et al. 2004; Fig. 2). Both the dephosphorylation of TPP and HMP-PP by NudJ may diminish the available pool of TPP. Interestingly, the enzyme ThiM is also involved in both DMAP and TPP synthesis. Like NudJ, ThiM can produce DMAP (using prenol rather than DMAPP as a substrate; Wang et al. 2018). However, in the TPP salvage pathway ThiM has an opposing effect to NudJ: it phosphorylates 4-methyl-5-hydroxyethylthiazole (HET) to produce HET-*P*, aiding rather than antagonising the salvage of TPP (Mizote and Nakayama 1989), (Fig. 2).

We therefore hypothesise that NudJ affects mutation rate either via its role in DMAP or in TPP metabolism. Because ThiM has a similar role to NudJ in one case and an antagonistic role in the other, both these hypotheses may be tested via the Δ*thiM* mutant. If what matters for mutation rate is the shared role of ThiM and NudJ in DMAP formation, we would expect a decreased mutation rate in a Δ*thiM* strain. On the other hand, if TPP metabolism is key to mutation rate, we would expect an increase of mutation rate in the Δ*thiM* strain. We found a non-significant decrease in the mutation rate of Δ*thiM* compared to the wildtype (*t*_DF = 283_ = −0.599, *P*  *=* 0.549, Statistical Model 1; supplementary fig. S3, Supplementary Material online). It is therefore more likely that NudJ affects the mutation rate via its role in DMAPP dephosphorylation than in thiamin salvage. The lack of a significant effect of *thiM* deletion on the mutation rate is consistent either with some role of DMAPP dephosphorylation or with NudJ's role in mutagenesis being unrelated to either its role in DMAP production or in thiamin salvage.

NudJ has also been shown to dephosphorylate all deoxy- and ribonucleotides in their tri- and dinucleotide forms with a preference for GDP (Xu et al. 2006). The role of NudJ in mutagenesis may come via altering the nucleotide pool composition rather than through its activity in DMAP production and thiamin salvage. We hypothesize that NudJ affects the mutation rate via elevation of the internal nucleotide pool (similar to the “dNTP mutator” effect described by Gon et al. [2011]). To test this hypothesis, we measured the internal ATP concentration, the most abundant nucleotide, in cultures of the wildtype and the Nudix hydrolase knockouts. Δ*nudJ* shows the lowest internal ATP concentration observed across the tested strains, with an ATP concentration significantly lower than the wildtype (*t*_DF = 33_ = −2.82, *P* = 0.00794, Statistical Model 2, supplementary fig. S4, Supplementary Material online). These results are consistent with the hypothesis that NudJ contributes to mutagenesis via elevation of the internal nucleotide pool.

### Mutations in Genes Associated With the Outer Membrane Reverse the Δ*nudJ* Antimutator Phenotype

**Fig. 3.**
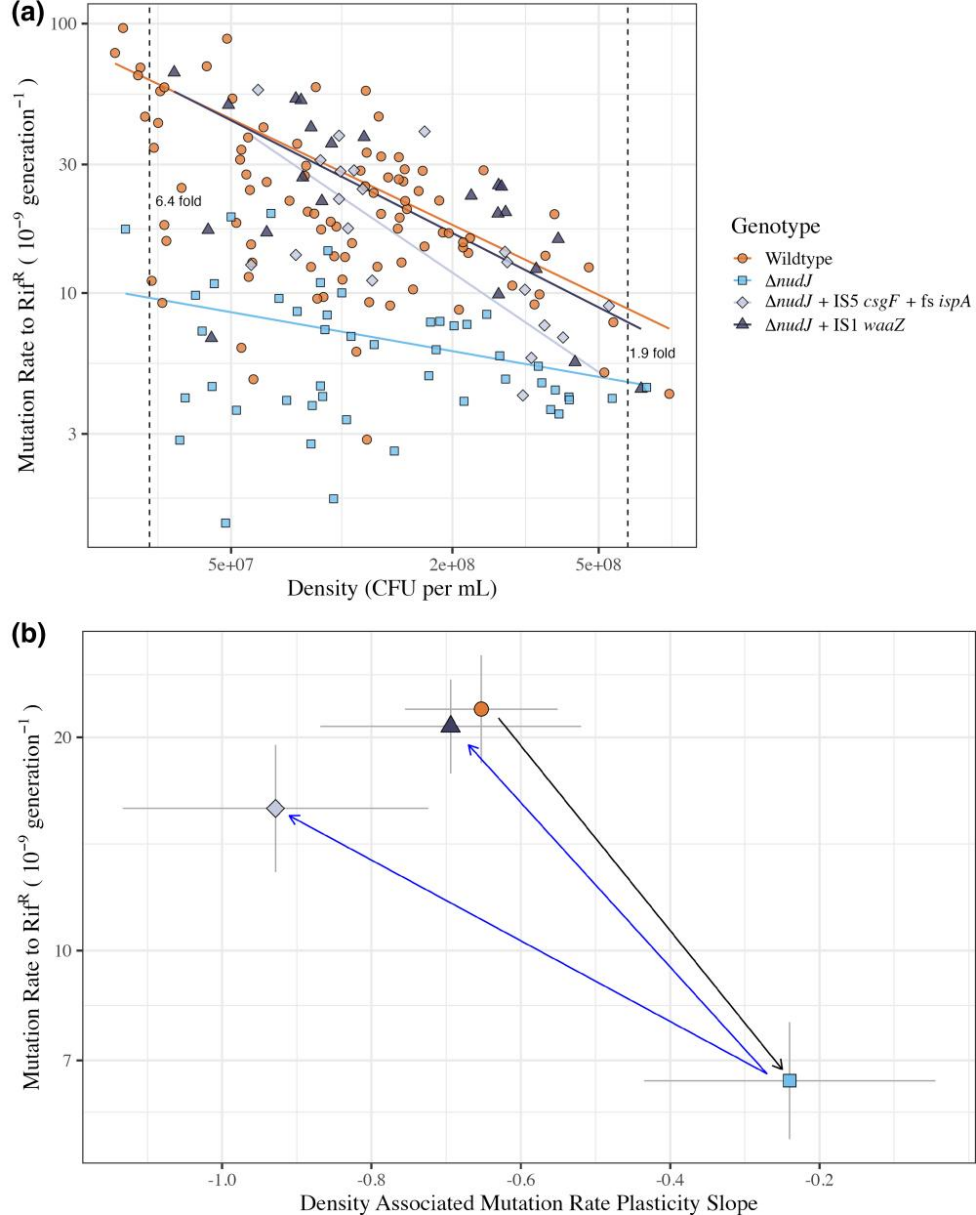
Mutation rates in wildtype BW25113 and Δ*nudJ* strains. a) Mutation rate to rifampicin resistance (mutational events per generation [×10^9^]) plotted as a function of final population density (CFU per mL). Orange circles: BW25113 wildtype (*N*_Fluctuation Assays_ = 92, *N*_Parallel Cultures_ = 1613); Pale blue squares: Δ*nudJ* (*N*_FA_ = 47, *N*_PC_ = 1089); Pale grey diamonds: Δ*nudJ* + *waaZ* IS1(*N*_FA_ = 22, *N*_PC_ = 478); Dark grey triangles: Δ*nudJ* + *csgF* IS5 + *ispA* FS (*N*_FA_ = 20, *N*_PC_ = 321). Dashed lines indicate population densities of 3 × 10^7^ and 6 × 10^8^ at which the fold difference in mutation rate between the wildtype BW25113 and Δ*nudJ* is estimated to be 6.4-fold (95% CI: 4.3–9.72) and 1.9-fold (95% CI: 1.4–2.5), respectively. b) Mutation rate (intercept at a density of 1.47 × 10^8^) and slope (density associated mutation rate plasticity) of lines of the fitted regressions shown in Fig. 2a with 95% confidence intervals (Statistical Model 1). Black arrow indicates the change in phenotype caused by *nudJ* deletion and blue arrows indicate the reversion in phenotype caused by secondary mutations. Mutation rates for wildtype and Δ*nudJ* are as in Fig. 1. Raw data can be found in data file S3.

Significant mutation rate reductions in wildtype *E. coli* are uncommon. We therefore verified our findings in the Δ*nudJ* strain by assaying mutation rates in a second independent *nudJ* deletant as well as 3 further colonies from the original deletant stock (totalling 5 strains: 4 from the original deletant and 1 from an independent deletant, all derived from the Keio collection [Baba et al. 2006]). Within this set of *nudJ* knockouts, we found clear variation in mutation rates; three show the previously observed antimutator phenotype while two show a wildtype mutation rate (supplementary fig. S5, Supplementary Material online). All of the strains with an antimutator phenotype derive from the original knockout and have no secondary mutations in coding regions. In contrast, the two strains showing a wildtype mutation rate carry secondary mutations in coding regions: the first, from the original knockout, has an IS1 insertion within the first quarter of the *waaZ* coding sequence; the second, deriving from an independent knockout, has both an IS5 insertion in the first half of the *csgF* coding sequence and a frameshift + CG mutation within the first 15 bases of the *ispA* coding sequence (Fig. 3). It is likely that these insertion sequence (ISs) and frameshift mutations are functionally equivalent to whole gene deletions. In both of these revertant strains, IS elements disrupt genes involved in the barrier between cells and their environment; WaaZ is involved in formation of the lipopolysaccharide core which forms part of the outer membrane (Frirdich et al. 2003) whilst CsgF is required for efficient assembly of curli, the major proteinaceous component of the extracellular matrix (Barnhart and Chapman 2006). Mutants in the *waaQGPSBIJYZK* operon have been shown to be deficient in curli production (Smith et al. 2017), potentially explaining the similar phenotypes of strains with each of these secondary mutations.

These secondary mutations may cause an increase in mutation rate independent of the *nudJ* allele, balancing the antimutator effect of *nudJ* deletion. Alternatively, these mutations might have no effect on their own, but epistatically interact with the *nudJ* deletion to revert the mutation rate. To test this, we assayed the mutation rate of a strain with *waaZ* deleted in a wildtype background. We focus on the *waaZ* mutant rather than the *csgF* + *ispA* mutant due to the conditional essentially of *ispA* (Fujisaki et al. 2005; Baba et al. 2006)*. waaZ* deletion failed to detectably increase the mutation rate (*t*_DF_  _=_  _18_ = −1.45, *P* = 0.919, supplementary fig. S6, Supplementary Material online, Statistical Model 3). Thus, *waaZ* disruption has an epistatic effect with *nudJ* deletion, significantly elevating the mutation rate in the Δ*nudJ* background (*t*_DF_  _=_  _102_ = 11.7, *P* = 4.41 × 10^−26^, Fig. 3, Statistical Model 1) but not the wildtype background.

To further test the hypothesis that NudJ modifies mutation rates via elevation of the internal nucleotide pool, we also measured internal ATP in the revertant Δ*nudJ* strains (supplementary fig. S4, Supplementary Material online). We find that, unlike Δ*nudJ* alone, Δ*nudJ* + IS1 *waaZ* does not significantly differ from the wildtype (*t*_DF = 33_ = −0.0155, *P* = 0.988, Statistical Model 2) whilst Δ*nudJ* + IS5 *csgF* + fs *ispA* actually increases the internal ATP concentration relative to the wildtype (*t*_DF = 33_ = 3.47, *P* = 0.00191, Statistical Model 2), contrasting with the decrease in ATP pool seen in the original Δ*nudJ* strain. This supports our hypothesis that the mutation rate decrease seen in Δ*nudJ* is caused by a change in the internal nucleotide pool, as indicated by a depletion of ATP, which is recovered to wildtype concentrations, or above, in the revertant strains.

A possible confounding factor in our discovery of Δ*nudJ* as an antimutator is that this could simply reflect the known association between growth rate and mutation rate (Novick and Szilard 1950; Chu et al. 2018; Maharjan and Ferenci 2018; Liu and Zhang 2019). Previous work has generally identified a negative correlation between microbial mutation rates per generation and growth rate (Novick and Szilard 1950; Maharjan and Ferenci 2018; Liu and Zhang 2019). We would therefore expect that if growth rate is driving the antimutator phenotype of Δ*nudJ*, this strain will have an increased growth rate. Instead, we see no change in the growth rate of the Δ*nudJ* strain (*t*_DF = 26_ = 0.641, *P* = 0.53, supplementary figs. S7 and S8, Supplementary Material online) although the carrying capacity is reduced in this strain at high but not low density (*t*_DF = 24_ = −7.16, *P* = 2.1 × 10^−7^ and *t*_DF = 24_ = −0.261, *P* = 0.8, respectively, supplementary figs. S7 and S8, Supplementary Material online; see also supplementary fig. S9, Supplementary Material online). This reduction in carrying capacity at high density is not restored by the *waaZ* insertion, which restores the wildtype mutation rate (*t*_DF = 24_ = −5.59, *P* = 9.5 × 10^−6^  supplementary figs. S7 and S8, Supplementary Material online; see also supplementary fig. S9, Supplementary Material online), again indicating that the role of NudJ in mutation rate determination is not due to changes in growth dynamics. The recovery of wildtype mutation rate in the Δ*nudJ* + IS1 *waaZ* strain without growth defect recovery suggests that these traits are evolving independently.

### The Antimutator Phenotype of Δ*nudJ* is Environment-dependent

Mutation rate is known to be negatively correlated with population density in wildtype *E. coli* (Krašovec et al. 2014). In addition to showing a reduced mutation rate, Δ*nudJ* also shows a significantly weaker relationship between density and mutation rate compared to the wildtype (Δ*nudJ* slope = −0.23 ± 0.19, wt slope = −0.65 ± 0.11, 95% CI, *t*_DF_  _=_  _102_ = 4.17, *P* = 1.24 × 10^−4^, Statistical Model 1). Because of this change in environmental responsiveness, the antimutator phenotype of Δ*nudJ* is the strongest at low densities. For example, at a low population density of 3 × 10^7^ CFU mL^−1^, we see a mutation rate decrease of over 6-fold (95% CI: 4.3–9.72) in Δ*nudJ* as compared to the wildtype, in contrast at a higher density of 6 × 10^8^ CFU mL^−1^ Δ*nudJ* only shows a 1.9-fold reduction in mutation rate (95% CI: 1.4–2.5, Fig. 3). DAMP is largely driven by elevation in the rate of A > G mutations at low density (Gifford et al. 2024); we, therefore, hypothesized that Δ*nudJ* may be less prone to this class of SNP.

### Mutational Spectrum is Depleted in A > G Transitions in Antimutator Δ*nudJ*

To test for changes in mutational spectrum in the Δ*nudJ* background, we collected spontaneously occurring rifampicin resistant mutants from mutation rate assays with the wildtype, Δ*nudJ* and Δ*nudJ* + IS1 *waaZ* strains. These mutants were collected from low density populations (defined as those grown in 80 mg.L^−1^ glucose) as it is in this condition that we see the largest difference in mutation rate (Fig. 3) and therefore might expect to see the greatest deviation in spectra between Δ*nudJ* and the wildtype. We sequenced the rifampicin resistance determining region (RRDR) of *rpoB* in resistant strains with each of these three genetic backgrounds. Although the distribution of mutational classes observed within the RRDR does not reflect that across the whole genome, this method does allow for robust comparison of the spectra of point mutations among strains (Garibyan 2003).

**Fig. 4.**
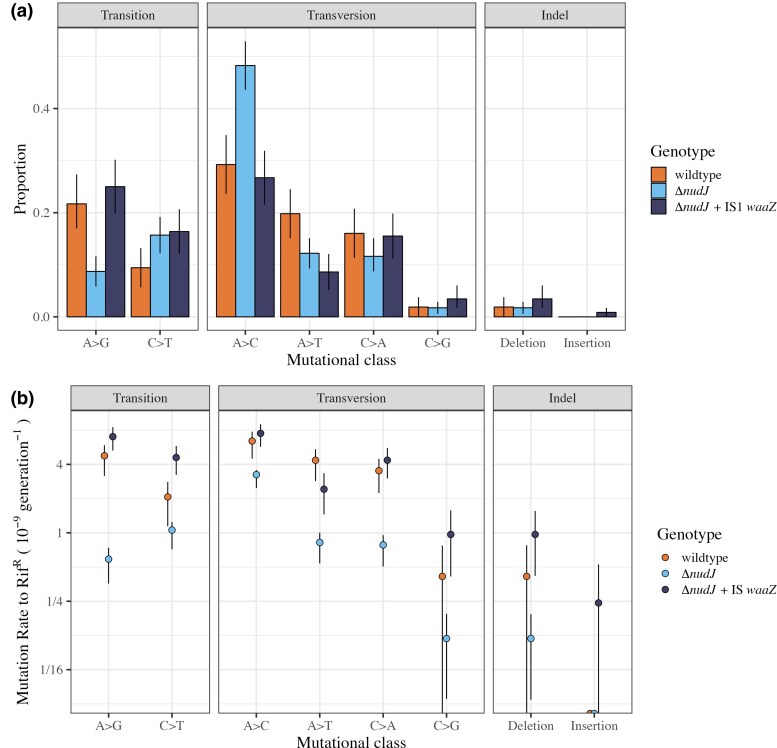
Mutational spectra of rifampicin resistance (rifR) mutations in wildtype BW25113, Δ*nudJ* and Δ*nudJ* + *waaZ* IS1 genetic backgrounds. a) Proportion of identified rifR mutations in the RRDR from each mutational class are shown for wildtype BW25113 in orange (*N* = 106), Δ*nudJ* in pale blue (*N* = 172), and Δ*nudJ* + *waaZ* IS1 in dark blue (*N* = 116). All mutations are listed in supplementary table S2, Supplementary Material online. b) Mutation rate to rifR by each mutational class is calculated by multiplying the proportion of rifR mutants in each mutational class and the mutation rate to rifR among all low density (80 mg glucose per l) fluctuation assays from which mutants were sequenced. Proportions are as in Fig. 4a. BW25113 wildtype is shown in orange (*N_FA_* = 5, *N_PC_* = 202), Δ*nudJ* in pale blue (*N_FA_* = 11), *N_PC_* = 464), and Δ*nudJ* + *waaZ* IS1 in dark blue (*N_FA_* = 5, *N_PC_* = 181). Note log scale *y* axis in b) Raw data can be found in supplementary data files S2 and S3, Supplementary Material online. For both subplots, error bars represent 80% confidence intervals calculated by bootstrapping.

Δ*nudJ* shows a significantly different mutational spectrum to the wildtype ancestor (LR _DF_  _=_  _7_ = 16.9, *P* = 0.036, Statistical Model 4). However, the secondary mutation in Δ*nudJ* + IS1 *waaZ* causes the mutational spectrum to revert to that of the wildtype (LR _DF_  _=_  _7_ = 5.8, *P* = 0.57, Statistical Model 4, Fig. 4). The most striking differences in the spectrum introduced by Δ*nudJ* are a decrease of over 50% in the proportion of A > G transitions (χ^2^_DF = 1_ = 7.65, *P*  *=* 0.0227), and a concurrent increase in A > C transversions (χ^2^_DF = 1_ = 9.68, *P*  *=* 0.0149) (supplementary table S3, Supplementary Material online). This is consistent with our prediction that a reduction in A > G transitions is driving the observed reduction in DAMP (Gifford et al. 2024) (Fig. 3). While the relative shifts in the mutational spectrum of Δ*nudJ* potentially account for its loss of DAMP, it is important to note that the absolute rates of all mutational classes, including A > C transversions, are decreased to differing degrees in Δ*nudJ* (Fig. 4b). The elevated rate of A > G transitions seen here in the presence of NudJ has also been observed under thymine starvation where this phenotype has been suggested to result from imbalances in the intracellular nucleotide pool (Kunz and Glickman 1985). This is consistent with our hypothesis that the role of NudJ in mutagenesis is driven by its nucleotide dephosphorylase activity, altering the intracellular nucleotide pool composition (Xu et al. 2006).

One alternative hypothesis for the reduced mutation rate of Δ*nudJ* to rifampicin resistance (rifR) is that a smaller possible pool of amino acid substitutions provides rifampicin resistance in the Δ*nudJ* background than in the wildtype. Across 169 mutants carrying single base pair substitution mutations in the Δ*nudJ* background 35 unique amino acid (AA) substitutions were observed (supplementary table S2, Supplementary Material online, supplementary fig. S10, Supplementary Material online). By randomly subsetting these mutants to the same size as the wildtype sample (104), we find a median of 30 unique AA substitutions (95% prediction interval 26 to 33, 1 × 10^6^ repeats), not significantly different from the 32 actually observed in the wildtype. This suggests that the possible pool of amino acid substitutions providing rifampicin resistance does not differ in size between the wildtype and Δ*nudJ* backgrounds.

### Mutational Hotspots in *rpoB* do not Differ Between the Δ*nudJ* Antimutator and the Wildtype

RifR mutations are not only biased by mutational class but also by genomic location, indicating the presence of mutational hot and cold spots within the sequenced region of *rpoB* (supplementary fig. S11, Supplementary Material online) (as previously observed e.g. Wolff et al. 2004). For example, while we find A > C transversions at position 1,687 (T563P) to account for 66 of 145 observed A > C mutations observed across all strains, the least frequent A > C transversion, at position 1,598 (L533R) was observed only 3 times. We find a significant effect of position on mutant frequency, accounting for differences in the relative rates of mutational classes, indicating that hotspots exist in the rifampicin resistance determining region of *rpoB* (Deviance_DF = 48_ = 86.9, *P* = 5.03 × 10^−4^). This is a conservative estimate given the likely existence of unobserved rifR mutants (Sun and Lind 2023). These hotspots do not correlate with fitness of the resultant mutant, providing no evidence of biased mutant selection or an evolutionary bias towards low cost resistance mutations (supplementary fig. S12, Supplementary Material online). We also tested if the degree to which a rifR mutant is enriched or depleted in the Δ*nudJ* or Δ*nudJ* + IS1 *waaZ* background, relative to the wildtype, is correlated with fitness, finding no correlation (supplementary fig. S13, Supplementary Material online). This observed independence of mutational frequency and mutant fitness has also been previously reported in *Pseudomonas aeruginosa* (MacLean et al. 2010).

It is possible that these hotspots, like the mutational spectra, will differ between genetic backgrounds. This would indicate that not only does *nudJ* deletion bias *E. coli* towards different mutational classes but towards different specific mutations within these classes. We find no significant evidence for changes in the strengths of mutational hotspots within any mutational class between the wildtype, Δ*nudJ* and Δ*nudJ* + IS1 *waaZ* (Deviance_DF = 45_ = 14.9, *P* > 0.99, Statistical Model 5, supplementary fig. S14, Supplementary Material online).

### The Available Distribution of Growth Effects is Altered by Mutational Spectrum

Because of the altered mutational spectrum of Δ*nudJ*, the probabilities of each particular spontaneous resistance mutation arising, i.e. mutation accessibility, is changed. For example, 12% of all rifR mutants identified in the wildtype background were D516G, caused by an A > G transition. In contrast, only 5% of rifR mutants in the Δ*nudJ* background were accounted for by this DNA and amino acid (AA) substitution. Particularly with rifampicin, where the target of resistance, *rpoB*, is an essential gene, different resistance mutations have different fitness costs associated with them (MacLean et al. 2010; Maharjan and Ferenci 2017). We therefore hypothesized that the distribution of fitness effects (DFE) of accessed rifR mutants (termed the DFE_β_ by Tuffaha et al. 2023) will differ depending on the genetic background in which they evolved. The DFE_β_ can be compared to the null expectation that each mutant will be observed an equal number of times, we will refer to this distribution as the DFE_all_ as in Tuffaha et al. 2023.

In order to characterise the DFE_β_ for each of our strains we measured the growth effects (as the area under the curve [AUC] of 24 h growth curves) of 27 RpoB amino acid substitutions. This type of growth assay is a good proxy for competitive fitness (see Fig. 3b in Vogwill and MacLean 2015). RifR mutant growth was measured in the MG1655 wildtype background, although this strain differs from the BW25113 background used for all fluctuation assay experiments both are similar, K12, strains (Long et al. 2017). The 27 amino acid substitutions selected account for 82.5% (325/394) of our mutants with identified mutations (the remaining 17.5% being 22 rare mutations). Given the environmental dependence of the fitness effects of *rpoB* mutations (Hall 2013; Gifford et al. 2016; Soley et al. 2023), we assessed mutant growth across 12 different environments to achieve a more general characterization of the impact of mutational spectrum on the DFE_β_. Our environments include all possible combinations of temperature (25°C vs. 37°C vs. 42°C), nutrient (minimal M9 vs. rich LB), and rifampicin (presence vs. absence). A weak correlation between the fitness effects of rifR mutations and their destabilising effect on the RpoB subunit is observed in this dataset (see supplementary Text, fig. S15 and S16, Supplementary Material online).

To test the hypothesis that mutants expected to evolve in different backgrounds will have different fitness distributions, we weighted the growth measured for the *rpoB* mutants in the wildtype MG1655 background according to the estimated probability of observing the underlying DNA mutation in each strain's mutational spectrum and compared those strains (see Statistical Model 6). We find that mutants evolved in the Δ*nudJ* background have a significantly lower average fitness (AUC measured in the wt MG1655 background) than those evolved in the BW25113 wildtype background (AUC measured in the wt MG1655 background) (*t*_DF = 1572_ = −3.84, *P* = 1.29 × 10^−4^, Statistical Model 6, supplementary fig. S17, Supplementary Material online). Therefore, not only is rifampicin resistance observed less frequently in Δ*nudJ*, due to the reduced mutation rate, but when it does arise, greater fitness costs are typically incurred. We can also estimate the fitness costs of rifR from the data going into our mutation rate estimates. These are counts of rifR colony forming units, which will be reduced if resistance mutations carry a cost in the non-selective environment of the fluctuation assay. By co-estimating the average mutant fitness with the mutation rate, we see that rifR mutants do indeed show a lower average fitness in the Δ*nudJ* background relative to the wildtype (supplementary fig. S18, Supplementary Material online).

The shift towards lower fitness mutants is driven by a single A > C mutational hotspot at position 1687 in *rpoB*, which produces the amino acid substitution T563P (supplementary fig. S14, Supplementary Material online). The T563P mutant shows the poorest growth (as measured by AUC) in 10 of the 12 environments tested (See supplementary data file x, Supplementary Material online). Due to the elevated proportion of A > C mutations in the Δ*nudJ* strain, this mutant is accessed substantially more frequently by the Δ*nudJ* strain than by the wildtype (T563P makes up 24.4% vs. 12.3% of mutants respectively; see supplementary fig. S13, Supplementary Material online). The importance of this single low fitness hotspot in driving the reduced fitness of rifR mutants accessed by the Δ*nudJ* strain can be evidenced by removing this amino acid substitution from our analysis, leaving no significant difference between the fitness of mutants accessed by Δ*nudJ* versus the wildtype (*t* = 1.49, *P* = 0.136). This finding highlights that a single mutational hotspot with an extreme fitness effect can drive significant changes in the DFE of resistance mutations when the target size is relatively small. Small mutational targets are common for antibiotic resistance phenotypes (e.g. quinolone nalidixic acid has a target size of ∼20 possible mutations in *gyrA/B* (Yamagishi et al. 1986; Harmand et al. 2017) while aminoglycoside streptomycin has even fewer possible resistance mutations in *rpsL* (Bohman et al. 1984)).

## Discussion

By characterizing the roles of 10 Nudix hydrolases in spontaneous mutagenesis, we have highlighted disruption of NudJ, NudB, NudF, and NudH as novel pathways to reduced mutation rates in *E. coli* (Fig. 1). We focused on the *nudJ* deletant, finding that not only is the mutation rate reduced, but the mutational spectrum is also significantly altered in this strain (Fig. 4). Δ*nudJ* has a mutational spectrum significantly depleted in A > G transitions and instead has a much higher proportion of A > C transversions than the wildtype. This change in spectrum causes Δ*nudJ* to preferentially access rifampicin resistance mutations of significantly lower fitness than those accessed by the wildtype (supplementary fig. S17, Supplementary Material online), with low fitness being associated with a predicted destabilising effect of the resistance mutations on the RNA polymerase subunit (supplementary fig. S16, Supplementary Material online). Both the antimutator phenotype and altered mutational spectrum of Δ*nudJ* are reversed by a secondary mutation in *waaZ*, demonstrating the complexities underlying the genetic determination of microbial mutation rates (Figs. 3 and 4). Taking Δ*nudJ* as a model, we show how multiple factors influencing evolvability (mutation rate, mutation rate plasticity and shifts in the available DFE caused by mutational spectrum) can be simultaneously altered by a single gene knockout. This study highlights the importance of considering not only the mutation rate but also the mutational spectrum and environmental responsiveness in these traits if we are to understand the evolutionary impacts of mutation rate modifying alleles. If leveraged well, these additional properties of antimutators will enhance our ability to control evolutionary trajectories.

### Mutational Hotspots

Although we find significant evidence of mutational hotspots in *rpoB* (supplementary fig. S11, Supplementary Material online), we do not find the location of these hotspots to be altered in the Δ*nudJ* antimutator strain (supplementary fig. S14, Supplementary Material online). This echoes the findings of Shepherd et al. (2022) who find that a transversion based hotspot identified in wildtype *Pseudomonas fluorescens* remains active in a transition biased Δ*mutS* mutator. These findings indicate that, while changes in mutational spectra and mutation rates are commonly correlated due to a shared cause (e.g. Lee et al. 2012; Garushyants et al. 2024), in this case most likely associated with the nucleotide pool, mutational hotspots are controlled by distinct mechanisms (reviewed by Horton and Taylor 2023). While mutation rates and spectra are typically controlled by the genome wide effects of mutation rate modifying alleles, mutational hotspots are typically determined by local sequence context. For example, the most frequently observed mutation in this study (an A > C mutation at position 1687 leading to AA substitution T563P) alters the sequence at positions 1685 to 1691 from “AA**A**CCCC” to “AA**C**CCCC”; this is consistent with the mutagenic effect of G runs 5′ of a T nucleotide (as would be seen on the opposite, leading, strand, reading “GGGG**T**TT” in the wildtype) (Cherry 2023).

### Synergy Between Mutation Rates and Biases

The mutation supply rate in a population is the product of the per-genome mutation rate and population size (de Visser et al. 1999), making reduced mutation rates likely to be more consequential in small populations in which mutational supply is already more limited. Where mutation supply, and hence clonal interference, is limited, adaptation is likely to proceed by the “survival of the first arrival” (MacLean et al. 2010; Cano et al. 2022). Although strongest under low mutational supply it is worth noting that mutational biases can still impact evolutionary paths under a relatively high mutation supply rate (Yampolsky and Stoltzfus 2001; Cano et al. 2022). Both mutation rate and mutational spectrum can therefore play important roles in the evolvability of a population with this effect depending on demographic factors. The possible contribution of mutations to adaptation depends on their phenotypic effects, which can vary between mutational classes (e.g. Couce and Tenaillon 2019; Sane et al. 2023, 2025). Given that mutation rate changes often come with shifts in mutational spectrum (Foster et al. 2015; Garushyants et al. 2024), there is potential for synergism or antagonism between the effects of mutation rate and mutational spectrum on the evolvability of a population. In the case of the Δ*nudJ* strain, we see a synergistic effect where both mutation rate and spectrum limit the potential for adaptive evolution to a rifampicin-containing environment.

### Evolvability to Other Selective Pressures

It is entirely plausible that, had we considered another drug, the mutational spectrum of Δ*nudJ* may instead have provided access to mutants with greater average fitness than the wildtype spectrum. Using bootstrap sampling of published data, we show that the mutational spectrum of Δ*nudJ* will facilitate access to nalidixic acid resistance mutations with lower fitness than those accessed by the wildtype (supplementary fig. S19, Supplementary Material online). In contrast, the relative growth effects of trimethoprim resistance mutations accessed under the mutational spectra of Δ*nudJ* and the wildtype vary systematically by the environment in which growth is measured (supplementary fig. S19, Supplementary Material online). These estimates do not account for the effect of mutational hotspots (see Methods for details). This omission reveals that even in the absence of strong mutational hotspots with extreme fitness values (as seen here for rifampicin), the Δ*nudJ* mutational spectrum can substantially shift the expected distribution of fitness effects of resistance.

We can also consider previous work by Couce et al. (2013) who show that a Δ*mutT* mutator is biased towards higher cost mutations to rifampicin resistance but towards less costly streptomycin resistance mutations when compared to the C > A biased Δ*mutY* mutator. Given the A > C dominated mutational spectrum of both Δ*mutT* and Δ*nudJ*, we could infer that a NudJ inhibitor would be more effective in preventing rifampicin resistance compared to streptomycin resistance. Variation in the cost of resistance will not only alter population growth during antibiotic therapy but will also alter the chances of the sensitive wildtype strain re-invading following the withdrawal of antibiotic therapy (Guo et al. 2024).

### The Drift Barrier Hypothesis

The rarity of antimutator alleles is predicted by the “drift-barrier hypothesis” which proposes that mutation rates are generally under directional selection towards a minimum and only prevented from reaching this minimum by the effect of genetic drift (Sung et al. 2012; though challenged by Liu and Zhang 2021). The large effective population size of *E. coli* would suggest that most easily accessible alleles, such as those causing single gene disruption, which reduce mutation rates, should already be fixed. However, the assumption that antimutators should be selected for ignores fitness effects; for example, mutation rate modifiers may have direct pleiotropic fitness consequences (Saint-Ruf et al. 2007; Fitzsimmons et al. 2018) and the fitness consequences of the particular mutations they modify may vary (Couce et al. 2013; Sane et al. 2023, 2025). For example, the Δ*nudJ* strain used here has a significantly reduced carrying capacity under high density conditions (supplementary fig. S7, Supplementary Material online). A second important consideration is the known environment-by-genotype interactions, which determine microbial mutation rates (e.g. Fowler et al. 1994; Green et al. 2024). Because of the environmental effect, an allele that appears to elevate mutation rates in the lab may not do so in the natural habitat of the organism and may therefore persist in the wild population. Therefore, the identification of antimutators under lab conditions does not itself refute the drift-barrier hypothesis and the scope for identification of as yet undiscovered antimutators may be greater than previously assumed.

### Antievolution Drugs

Reducing NudJ activity, here by gene deletion but potentially in future via an inhibitor drug, reduces access to certain antibiotic resistance mutations. This aligns with the concept of antievolution drugs, capable of slowing the evolution of resistance during antibiotic treatment, sufficient that an infection is expunged either by the antibiotic or immune system before resistance arises (Ragheb et al. 2019). This has been explored via the inhibition of RecA, which promotes antibiotic induced mutagenesis. Its inhibition can potentiate the activity of multiple classes of antibiotics (Alam et al. 2016). Bacterial populations can also evolve antimicrobial resistance through horizontal gene transfer, which is similarly potentially susceptible to anti-evolution strategies: chemically inhibiting bacterial competence can reduce the spread of antimicrobial resistance genes within an infectious population (Domenech et al. 2020). It has been observed that antibiotic tolerance, persistence and resilience can provide a population with greater opportunity to evolve bona fide antibiotic resistance (Brauner et al. 2016; Liu et al. 2022). Therefore, targeting resilience, tolerance or persistence may also prove to be useful avenues for anti-evolution drug research.

### Comparisons to Other Antimutators

Similar to Δ*nudJ*, deletion of either *mfd* in multiple bacterial species or *PSP2* in *S. cerevisiae* have previously been shown to reduce mutation rates (Ragheb et al. 2019; Liu and Zhang 2021). Mfd interacts with RNA polymerase (RNAP) to remove blocked RNAP and recruit repair enzymes to DNA lesions as well as aiding transcription of hard to transcribe regions (Selby et al. 1991; Ragheb et al. 2021), while PSP2 binds to specific mRNAs, promoting their translation (Liu and Zhang 2021). Both of these proteins are involved in RNA transcription or translation whereas NudJ interacts with single nucleotides, catalyzing the dephosphorylation of deoxy and ribo nucleotides in both the di and tri-phosphate forms (Xu et al. 2006). This contrast suggests that whilst the antimutator phenotypes of these strains are similar, the molecular mechanisms responsible are distinct.

The mutation rate reduction seen in Δ*mfd* strains (2- to 5-fold) is similar to our observations in Δ*nudJ* (2- to 6-fold). When compared to the >100 fold increases in mutation rate seen in some mutator strains, these changes are the modest. However, dramatic reductions in the ability of *Salmonella typhimurium* to evolve resistance to multiple antibiotics was seen in the absence of *mfd* (Ragheb et al. 2019); demonstrating that small mutation rate decreases can have a large impact on evolutionary trajectories. In that study, genetic mutator strains were frequently observed when the wildtype strain evolved in the presence of trimethoprim; however, the evolution of a mutator phenotype was never observed in the Δ*mfd* antimutator. Therefore, antimutators are not only less likely to access mutations providing antibiotic resistance but are also less likely to access mutations leading to a mutator phenotype, which can facilitate resistance evolution (Couce et al. 2015; Gifford et al. 2023; Elgrail et al. 2024 ). The logic behind this finding is that the greater a strain's mutation rate, the greater its chance of accessing mutation rate modifying alleles. This is reflected by frequent observations of both positive (Gifford et al. 2023; Elgrail et al. 2024) and negative (McDonald et al. 2012; Wielgoss et al. 2013; Sprouffske et al. 2018) mutation rate modifying alleles in mutator strains during experimental evolution.

A more similar case to *nudJ* is *purB* in *E. coli*, where a temperature sensitive mutation (*purB^ts^*) provides an antimutator phenotype (Geiger and Speyer 1977; though see Schaaper and Dunn 2001). Like NudJ, PurB is involved in nucleotide metabolism; PurB plays an essential role in purine biosynthesis (Zhang et al. 2008). Interestingly, *purB* and *nudJ* are only ∼1,800 bp apart in the *E. coli* genome and are also commonly co-expressed (Szklarczyk et al. 2022). This raises the possibility that the antimutator phenotypes of Δ*nudJ* and *purB^ts^* may share a common cause.

### Mechanisms of NudJ Induced Mutation

Mutator strains, with elevated mutation rates, often show dramatic changes in mutational spectra which reflect the role of the disrupted gene. For example, in Δ*mutT* strains, oxidized dGTP is no longer removed from the cytoplasm, leading to A:G mispairing events, which result in many A > C transversion mutations (Maki and Sekiguchi 1992). As well as having evolutionary implications, our finding of a significantly altered mutational spectrum in Δ*nudJ* provides hypotheses as to the mechanism of NudJ's role in mutagenesis. Our finding that *nudJ* deletion specifically decreases the rate of A > G transitions indicates that the presence of NudJ preferentially enables these mutations. An elevated rate of A > G transitions is also observed in *E. coli* starved of the nucleotide thymine, and this has been attributed to imbalances in the dNTP pool (Kunz and Glickman 1985). Consistent with the hypothesis that the mutagenic effects of NudJ act via imbalances of the nucleotide pool, we find significantly reduced concentrations of ATP in the *nudJ* deletant strain (supplementary fig. S4, Supplementary Material online).

It is possible the antimutator phenotype of Δ*nudJ* arises through the same mechanism as the antimutagenic effect of guanosine supplementation reported by Novick and Szilard 1952; which may or may not be associated with the mutational effects of thymine starvation (Kunz and Glickman 1985) and the “dNTP mutator” effect (Gon et al. 2011). Among 17 deoxy- and ribonucleosides tested by Xu et al. (2006), NudJ shows the greatest hydrolase activity towards GTP, it is therefore plausible that in the absence of NudJ more guanosine will accumulate in the cell. Gudkov et al. (2006) show that, in the presence of γ-irradiation or heating, guanosine can prevent the formation of 8-oxoguanine (8-OG) and the deamination of cytosine in DNA. These results suggest that guanosine will reduce the rates of multiple mutational classes, consistent with our observation that all mutational classes are suppressed, to differing extents, in Δ*nudJ* (Fig. 4b). We suggest that future work should measure individual nucleotide, dNDP, dNTP and NTP molecule concentrations in Δ*nudJ* and the wildtype to specifically quantify any imbalance in these nucleotide pools potentially using HPLC-based methods as in Buckstein et al. (2008) and Varik et al. (2017) . This will allow us to develop more specific hypotheses as to the mechanism(s) by which NudJ impacts mutagenesis.

Aside from its nucleoside dephosphorylase activity, NudJ (along with Nud B, F & I) is also known to dephosphorylate DMAPP to DMAP which is used to synthesise prFMN (Wang et al. 2018). prFMN is required by UbiD-like enzymes for the biosynthesis of ubiquinone (Wang et al. 2018). This link between antimutators Δ*nudJ,* Δ*nudB*, and Δ*nudF* and the known antioxidant ubiquinone (Søballe and Poole 2000) is intriguing given the known role of oxidative stress in determining mutation rates (e.g. Carvajal-Garcia et al. 2023; Lagage et al. 2023; Green et al. 2024). NudJ, NudF, and NudB are also known to hydrolyse isopentenyl diphosphate (IPP) to 3-methyl-3-butenol (Chou and Keasling 2012; Fig. 2). IPP and DMAPP (both degraded by NudJ) are combined to form geranyl-PP by IspA (Fujisaki et al. 1986; Fig. 2). This may explain the phenotypic reversion associated with a secondary *ispA* mutation in a Δ*nudJ* strain, while *nudJ* deletion may increase intracellular geranyl-PP concentrations, the disruption of *ispA* will reduce the genranyl-PP pool, countering the effect of *nudJ* deletion. However, as *ispA* and *csgF* are disrupted in the same Δ*nudJ* strain, it is possible that either one of these secondary mutations, or the combined effects of both, are responsible for the mutation rate reversion (Fig. 3, supplementary fig. S5, Supplementary Material online).

The contributions of WaaZ and potentially of CsgF to mutagenesis in the Δ*nudJ* background also remain to be explored. Both are disrupted by IS insertions in our revertant Δ*nudJ* strains, which may have an impact on not only their own transcription but that of the other genes in their operons (Jubelin et al. 2005; Lee et al. 2009; Kanai et al. 2022). We suggest that the reversal of the Δ*nudJ* phenotype by these secondary mutations in the outer membrane and extracellular matrix may operate by altering permeability of the cell to nucleotides, reversing the effect of the *nudJ* deletion on the internal nucleotide pool (Goyal et al. 2014; Wang et al. 2021). In line with this, we find that while the internal ATP concentration is decreased in Δ*nudJ*, these revertant strains show a return to wildtype or greater internal ATP concentrations (supplementary fig. S4, Supplementary Material online).

### The Need for Improved Mutation Rate Assays

This work exploits the rifampicin resistance phenotype to characterize mutation rates and spectra in a relatively high-throughput manner. This comes at the cost of measuring these phenotypes only in the RRDR locus of *rpoB*. Mutation rates measured either by fluctuation assays (FA) to rifampicin resistance or by genome wide mutation accumulation (MA) experiments (Foster 2006) are generally in agreement for known mutator strains (e.g. Δ*mutT* and Δ*mutL* characterized by both Krašovec et al. (2017) (FA) and by Foster et al. (2015) (MA) and Lee et al. (2012) (MA)). However, we are not aware of a direct comparison of these methods applied to an antimutator strain. In the future, MA experiments and/or sequence-based methods (Sloan et al. 2018; e.g. Schreck et al. 2023) should be applied to Δ*nudJ,* and the other antimutator Nudix hydrolase knockout strains, to estimate the mutation rate and spectra at a genome-wide scale.

There is potential for mutation rate and spectrum estimates to be confounded when using fluctuation assays. Firstly, a true reduction in mutation rate could lead to a false conclusion of altered mutational spectrum. Strains with lower mutation supply (de Visser et al. 1999) are less likely to evolve multiple rifR clones within a single population. If more than 1 rifR clone evolves in a population, the clone which goes through more divisions (determined by fitness and time of arrival) will be more abundant in the final population. We sequenced a single mutant from each parallel culture at random, the chances of a rifR clone being selected therefore depend on its frequency in the population. Because of this, a genotype with a higher mutation rate may be more likely to be enriched for rifR mutants with greater fitness. We do not believe this is driving our finding of an altered mutational spectrum in Δ*nudJ* as no correlation is observed between rifR mutant fitness and the degree to which it is enriched/depleted in the Δ*nudJ* background (supplementary fig. S13, Supplementary Material online, *t* = 0.16, *P* = 0.87). We also note that whilst the previously discussed low fitness mutant T563P mutant is enriched by 2-fold in the Δ*nudJ* background, mid-fitness A > C mutant I572S and high-fitness A > C mutant I572L are also enriched by 3-fold and 1.2-fold, respectively (supplementary fig. S13, Supplementary Material online). This suggests a true bias towards A > C mutations in Δ*nudJ.* Secondly, it is possible that a strain with a truly altered mutational spectrum will lead to a false conclusion of a reduced mutation rate *if* the change in spectrum favours low fitness mutants which sometimes do not form visible colonies by the point at which they are counted. Such an effect, however, would be specific to the relationship between spectrum and fitness at the particular marker locus chosen. To test this, we performed mutation rate assays testing different marker loci by using 2 other drugs, D-cycloserine (resistance gained by loss of function of *cycA* [Maharjan and Ferenci 2015]) and nalidixic acid (resistance gained by function retaining mutations in *gyrA/gyrB* [Yamagishi et al. 1986; Aleixandre et al. 1989; Harmand et al. 2017]). These alternative loci give a qualitatively similar result to the *rpoB* locus of rifampicin resistance in that the Δ*nudJ* mutant is estimated to have a lower mutation rate than wildtype supplementary fig. S2, Supplementary Material online. The consistency of the Δ*nudJ* antimutator phenotype across multiple marker loci suggests that this phenotype is not an artifact of the Δ*nudJ* mutational spectrum being shifted so as to access rifR mutants with lower chances of colony formation. However, in the future, genome-wide mutation rate estimates should be carried out for Δ*nudJ* and any other antimutator strains identified.

Given the previously discussed limitations of fluctuation assays and the labour intensive and selection biased (Fusco et al. 2016; Mahilkar et al. 2022; Wahl and Agashe 2022; Schreck et al. 2023) nature of MA experiments, it is clear that improved methods for microbial mutation rate estimation are needed. One potential direction for innovation is the development of population sequencing based assays with the potential to keep labour time low while generating genome-wide rate and spectrum estimates and avoiding strong effects of selection (Caravagna et al. 2020; Schreck et al. 2023; Furuyama et al. 2025; Green et al. 2025). However, for these methods to succeed in providing rapid, genome-wide mutation rate estimates for bacteria, increases in accessible sequencing depth will be necessary.

### Future Directions

The serendipitous reversion of Δ*nudJ* to a wildtype mutation rate in 2 independent strains during this investigation raises the questions of whether these reversions are under positive selection and how large the target size for reversion is. Although we find that minor growth defects in Δ*nudJ* are not recovered by Δ*nudJ* + *waaZ* IS1 or Δ*nudJ* + *csgF* IS5 + *ispA* FS (supplementary fig. S7–S9, Supplementary Material online), there may be other selective benefits not captured by growth curve assays. The reversion of mutation rates in these strains without clear recovery of growth defects suggests that mutation rates and growth phenotypes are genetically separable. The stability and independence of growth, nucleotide pool and mutation rate and spectrum phenotypes could be better understood in the future using experimental evolution studies on the strains characterized here.

## Conclusions

As well as identifying novel *E. coli* antimutators, this study also highlights the general importance of considering not only mutation rates but also mutational spectra in predicting evolutionary outcomes. Here, we use rifampicin resistance mutations to explore the effect of mutational spectra on fitness outcomes. The highly constrained nature of rifampicin resistance evolution is reflective of many antibiotics for which resistance can be gained by target site modification. Because of the more modest mutation rate changes seen in antimutators, as opposed to mutators, the effect of mutational spectrum is likely to be more pronounced. This makes the potential for rational manipulation of mutational spectrum by antievolution drugs, designed to reduce mutation rates, a feasible method for increasing their success. We therefore suggest that future work should assess the mutational spectra within known antimutators to build a more general picture of antimutator phenotypes. The environmental responsiveness of both mutation rates and spectra demonstrated in this study will be a hurdle to the development of such drugs. It is therefore important for future research on mutation rate manipulation to consider both environmental plasticity as well as the relationship between mutational spectrum and fitness outcomes in the environment of interest (e.g. an infection under antibiotic treatment). By bringing together all of these facets shaping evolvability, we will improve our ability to predict evolutionary outcomes, opening up the possibility of using this knowledge to manipulate the course of evolution.

## Materials and Methods

### Strains and Media

The wildtype BW25113 is the ancestor of Δ*waaZ,* Δ*thiM* and Δ*nudBCDEFGHIJK*, which were sourced from the Keio collection (Baba et al. 2006). All knockouts in the Keio collection appear twice; Δ*waaZ* was sourced from the first available knockout, Δ*thiM* from the second available knockout and all stocks of Δ*nudBCDEFGHIJK* from the first available knockout aside from Δ*nudJ* + fs *ispA* + IS5 *csgF*, which derives from the second *nudJ* knockout. Wildtype MG1655 was kindly provided by Karina Xavier and is the ancestor to all *rpoB* mutants used for rifR growth assays. These MG1655 *rpoB* mutants were collected and sequenced by (Gifford et al. 2024).

We used Milli-Q water for all media, all chemicals are supplied by Sigma-Aldrich unless stated otherwise. LB medium contained: 10 g of NaCl (Thermo Fisher Scientific), 5 g of yeast extract (Thermo Fisher Scientific), and 10 g of tryptone (Thermo Fisher Scientific) per litre. DM medium contained 0.5 g of C_6_H_5_Na_3_O_7_ ·2H_2_O, 1 g of (NH_4_)2SO_4_ (Thermo Fisher Scientific), 2 g of H_2_KO_4_P and 7 g of HK_2_O_4_P· 3H_2_O per litre; 100 mg L^−1^ MgSO_4_ ·7H_2_O (406 µmol), 4.4 μg L^−1^ thiamin hydrochloride and glucose to the desired concentration (80 to 1000 mg L^−1^) were added to DM after autoclaving. M9 medium contained 6.8 g of Na_2_HPO_4_, 3 g of KH_2_PO_4_, 0.5 g of NaCl, and 1 g of NH_4_Cl per litre; 100 µM CaCl_2_.2H_2_O, 2 mM MgSO_4_.7H_2_O, 3 µM thiamine hydrochloride, 11.2 mM glucose, and 0.2% casamino acids were added after autoclaving. Selective tetrazolium arabinose agar (TA) medium contained 10 g of tryptone, 1 g of yeast extract, 3.75 g of NaCl and 15 g bacto agar per litre; after autoclaving 3 g of arabinose and 0.05 g of 2,3,5-triphenyl-tetrazolium chloride were added per litre, this was supplemented with freshly prepared rifampicin (50 µg ml^−1^) dissolved in 1 mL of methanol. For all cell dilutions, sterile saline (8.5 g L^−1^ NaCl) was used.

### Fluctuation Assays

Fluctuation assays were conducted as described in Krašovec et al. (2019). Briefly, an ice scrape from glycerol stock was incubated in LB for 4 to 7 h (until visibly turbid). A 1,000-fold dilution then used for transfer to 10 mL overnight cultures in DM with the appropriate glucose concentration for acclimatization (80 to 1,000 mg.L^−1^). The density of these overnight cultures was then measured by OD and all cultures were diluted to a hypothetical OD of 3 × 10^−6^, equating to ∼3,000 CFU per mL, in fresh media. Large initial cultures were split into 19 to 20 parallel cultures of 0.5 to 1.25 mL in deep 96-well plates and incubated for 24 h. For each treatment, 3 “Nt” wells were taken and diluted appropriately before plating on non-selective TA agar to determine the final population size and density reached. The remaining wells were plated in their entirety on selective TA agar plates containing 50 mg.L^−1^ rifampicin and dried in a sterile biosafety cabinet. “Nt” plates were incubated for 24 h and selective plates for 44 to 48 h before colonies were counted. Estimates of mutational events were calculated using the maximum likelihood method as implemented by R package flan (Mazoyer et al. 2017). Because of the known effect of density on microbial mutation rates we estimate mutation rates for all strains at the mean density of the dataset (Fig. 1, supplementary fig. S1, Supplementary Material online) (Krašovec et al. 2014, 2017, 2018; Green et al. 2024).

Intracellular ATP was assayed at the end of most fluctuation assays (308/402) via luminescence using a Promega GloMax luminometer and the Promega Bac-Titer Glo kit, according to the manufacturer's instructions. We measured the luminescence of each culture 0.5 and 510 s after adding the Bac-Titer Glo reagent and calculated net luminescence as luminescence_510s_ − luminescence_0.5s_.

Liquid-based fluctuation assays were used in comparing the mutation rates of Δ*waaZ* and the wildtype (supplementary fig. S6, Supplementary Material online). The protocol remained the same as above aside from the identification of mutants in selective wells. Rather than plating the wells on rifampicin agar, fresh LB was added to the wells at this point along with rifampicin to a final concentration of (50 mg.L^−1^). After 2 d of further incubation, OD measurements were taken for all selective wells, OD > 0.2 was categorized as presence of a mutant and OD ≤ 0.2 as absence. Mutation rates were estimated using the P0 method as implemented by R package flan (Mazoyer et al. 2017).

### PCR and Sanger Sequencing

rifR mutants were picked from selective plates (selecting whichever colony has its centre nearest to the centre of the agar plate) at the end of low density fluctuation assays (defined as 80 mg.L^−1^ glucose) with the BW25113 wildtype, Δ*nudJ* or Δ*nudJ* + IS1 *waaZ* and grown to turbidity (4 to 5 h) in LB before freezing in 18% glycerol at −80°C. Mutants were then streaked from glycerol stocks onto TA agar and one colony was picked into 25 µL molecular grade H_2_O and mixed to break the cells by osmotic lysis, this solution was used as the DNA template. Twenty-five microliters of polymerase chain reaction (PCR) reactions contained 2.5 µL DNA template, 0.125 µL Phusion High-Fidelity DNA Polymerase (M0530S), 0.25 µL 500 μM forward and reverse primers, 1.25 µL DMSO, 5 µL buffer, 0.5 µL 10 mM dNTPs and 15.125 µL nuclease free H_2_O. Forward primer 5′-ATGATATCGACCACCTCGG-3′ and reverse primer 3′-TTCACCCGGATACATCTCG-5′ were used for all reactions as in (Gifford et al. 2024). PCR was run with the following protocol: (i) initial denaturation (98˚C for 5 min); (ii) denaturation (98˚C for 10 s); (iii) annealing (55˚C for 30 s); (iv) extension (72˚C for 1 min); (v) repeat steps 2 to 4 for 30 cycles; (vi) final extension (72˚C for 5 min); and (vii) hold at 4˚C. PCR product verification was performed by gel electrophoresis carried out on 1% agarose TAE gel with 0.1% SybrSafe stain. PCR products were submitted to Source BioScience for PCR product clean-up and sequencing (Source BioScience, Cheshire). Each product was submitted with either forward or reverse primer as the sequencing primer.

Raw Sanger sequencing results were converted to trimmed fasta files using R package SangeranayseR (Chao et al. 2021), mutations were then identified by alignment to the reference sequence (EG10894). All mutations were verified by visual inspection of the relevant chromatogram.

Of 463 sequenced isolates 2 had 2 mutations in the RRDR, these 4 mutations were excluded (Strain 1: wildtype position 1604 C > T + 1715 T > A, Strain 2: Δ*nudJ* 1598 T > G + 1604 C > T). This is because we cannot be sure of the order in which these mutations occurred; once a first mutation is acquired in *rpoB* the mutational spectrum for the second mutation may be altered due to pleiotropic effects of *rpoB* mutations (Bergval et al. 2007; Jago et al. 2025). Where 2 peaks were present at a given position, we required the primary peak to be over 3 × the height of a secondary peak in order to accept the mutation as conclusive, 2 mutations were excluded by this requirement (1 wildtype 1687 A > C, 1 nudJ + waaZ 1687 A > C). If we include the six filtered mutations, our finding of a significantly altered mutational spectrum in Δ*nudJ* versus wildtype is retained (LR = 17, *P* = 0.0354), as are the respective increase and decrease of A > C and A > G mutations in Δ*nudJ* (χ^2^ = 9.88 and *P* = 0.0134, χ^2^ = 7.3 and *P* = 0.0275, respectively). Our finding of reduced fitness in rifR mutants from the Δ*nudJ* background across all 12 tested environments is also retained (*t*_DF = 1555_ = −12.5, *P* = 6.88 × 10^−36^). Sixty-five of 463 sequenced mutants have no mutation in the RRDR (∼14% of filtered reads), this is to be expected as rifampicin resistance can be achieved by mutations outside the RRDR (Garibyan 2003). For the wildtype and Δ*nudJ* + IS1 *waaZ*, ∼18% of sequenced strains show no mutation in the RRDR (24/132 and 24/141, respectively) whereas for Δ*nudJ* <9% of sequenced strains (17/190) show no mutation in the RRDR. This is consistent with the findings of Garibyan 2003 who find that, in the wildtype background, 23/24 identified mutations outside the RRDR are A > T or A > G mutations, both proportionally underrepresented in Δ*nudJ* (Fig. 4).

The inclusion of block effects significantly improves the fit of a multinomial model of the effect of genotype on mutational spectrum (LR_df = 21_ = 36.2, *P*  *=* 0.0209). These block effects are likely the result of slight differences in conditions between experimental blocks; for example, changes in media composition and growth temperature are known to impact mutational spectrum (Chu et al. 2018; Liu and Zhang 2019). We therefore use a model accounting for block effects when analyzing mutational spectrum in order to account for this potentially confounding effect.

### Growth Curves

Growth curves of rifR mutants in the MG1655 background (used for AUC in supplementary figs. S12, S13, S16, S17, Supplementary Material online) were initiated by incubating 1 µL from frozen glycerol stocks in 200 µL LB at 37°C with 200 rpm shaking overnight. These cultures were then diluted 200-fold into appropriate fresh media (M9 supplemented with 2 g per L glucose or LB), with or without rifampicin (50 mg.L^−1^) and grown at 25, 37 or 42°C (giving a total of 12 possible environments). Clear 96-well plates were incubated for 24 h with OD readings taken every 30 min using BMG Omega plate reader. Thirty-four mutants were tested in total (27 of which bear mutations identified in the BW25113 wildtype, Δ*nudJ* or Δ*nudJ* + IS1 *waaZ* strains) with six repeats per mutant in each environment. Each mutant appeared twice within each 96-well plate, in order to minimize the effect of greater evaporation in the edge wells on our results, no mutant was placed in an edge well twice within a single plate. Summary statistics were computed for each growth curve individually using R package growthcurver (Sprouffske 2016, R Core Team, 2023) and means taken across each environment × mutant combination were paired to mutants identified in the BW25113 wildtype, Δ*nudJ* and Δ*nudJ* + IS1 *waaZ* strains for further analysis. Carrying out all rifR mutant growth curves in the wildtype MG1655 background prevents any assessment of potential epistasis between mutations in *rpoB* and other mutations in *nudJ, waaZ, csgF*, and *ispA*.

Growth curves of the BW25113 wildtype, Δ*nudJ*, and Δ*nudJ* + IS1 *waaZ* (as shown in supplementary figs. S7 and S8, Supplementary Material online) were prepared as for a fluctuation assay and grown in minimal DM with either 80 or 1,000 mg of glucose per L. Parallel cultures of 150 µL were grown in a shallow 400 µL well plates, and OD600 readings were taken every 10 min with shaking at 200 rpm for 1 min prior to each reading.

### Estimation of ΔΔg Caused by *rpoB* Mutations

ΔΔG of each mutant identified by RRDR sequencing was estimated using FoldX 5.0 (Schymkowitz et al. 2005). Reference protein structure for the RpoB protein was downloaded from alphafold (AF-P0A8V2-F1-v4) (Jumper et al. 2021; Varadi et al. 2024), based on UniProt entry P0A8V2 (Ovchinnikov et al. 1981; Bateman et al. 2023). Reference protein structure for the RNAP holoenzyme complex was downloaded from the Protein Data Bank (Braffman et al. 2019). Before introducing AA substitutions, the RpoB and RNAP protein structures were repaired using the FoldX command “RepairPDB” with options: *−ionStrength = 0.05 −pH*  *=*  *7 −water*  *=*  *CRYSTAL −vdwDesign = 2 −pdbHydrogens*  *=*  *FALSE.* FoldX command BuildModel was then used with default settings to estimate the ΔΔG resulting from each observed AA substitution for both RpoB and RNAP separately.

### Whole Genome Sequencing

Knockouts Δ*nudB,* Δ*nudF,* Δ*nudI*, and the wildtype BW25113 were sequenced to ≥30 × coverage with illumina short reads by MicrobesNG (https://microbesng.com); verification that strains carried the correct gene knockouts and no additional genic mutations was performed using breseq version 0.38.1 with default parameters. The wildtype BW25113 genome was used as a reference (Grenier et al. 2014) as modified by Gifford et al. (2023) to include improved IS annotation.

All Δ*nudJ* strains were sequenced by MicrobesNG *(*https://microbesng.com) using their hybrid service combining ≥30 × coverage with illumina short reads and ≥50 × coverage with Oxford nanopore technology long reads. All mutations were predicted using breseq (Deatherage and Barrick 2014) with the wildtype BW25113 genome as a reference (Grenier et al. 2014) modified to include improved IS annotation by Gifford et al. (2023). Mutations were also verified by using short reads from one Δ*nudJ* strain as a query and the full assembled genome from another Δ*nudJ* strain as the reference, as provided by MicrobesNG (https://microbesng.com). Both methods gave identical predictions.

### Bootstrapping Fitness Differences Between Antibiotic Resistance Mutations Accessed by Δ*nudJ* vs. the Wildtype

We analyzed the impact of mutational spectra on antibiotic resistance costs for nalidixic acid (Nal) and trimethoprim (Tmp) resistant mutants (supplementary fig. S19, Supplementary Material online). Strains one base-pair substitution removed from the wildtype from Harmand et al. (2017) (Nal) and Palmer et al. (2015) (Tmp) were included (compiled data taken from [Gifford et al. 2018]). Separately for each antibiotic, 100 mutants were sampled, with replacement, following the mutational spectra observed in Δ*nudJ* and the wildtype among rifampicin resistant mutants in this study (Fig. 4). The sampling probability for each mutation type was proportional to its frequency in the rifampicin resistance spectrum. For example, in the case of the wildtype, 28% of rifR mutants carry A > C transversions while only 10% are C > T transitions; therefore, 2.8 A > C mutants will be sampled for each C > T mutant sampled. The mean selection coefficient (nalidixic acid) or AUC of a 30 h growth curve (trimethoprim) was then calculated for the set of 100 mutants sampled by Δ*nudJ* and the wildtype in each tested environment and the difference between these mean values recorded. This bootstrapping procedure was repeated 1,000 times for each antibiotic. For nalidixic acid, 13 unique mutations each causing a distinct amino acid substitution were included while for trimethoprim 8 unique mutations, 1 in the promoter region, and 7 in the coding region (2 of which result in the same AA substitution), were included.

This approach does not consider the presence of mutational hotspots in any of the target genes considered (*rpoB, gyrA* & *folA* for rif, nal & tmp, respectively). It also does not account for differences in the spectrum of available mutations to each antibiotic (for example, 50% of possible resistance mutations to one drug may be A > G transitions while this type of mutation may account for only 10% of possible resistance mutations to another drug). These limitations are, to some extent, a result of incomplete knowledge of the entire set of mutational targets for any of these drugs. Even with extensive mutant selection and sequencing, mutational “coldspots” may remain unobserved (Sun and Lind 2023). Despite these limitations, this method provides a valuable first approximation of how the costs of antibiotic resistance may be influenced by the specific mutational spectra of Δ*nudJ* versus the wildtype.

### Statistical Methods

All statistical models associated with this study are detailed in the Supplementary Statistical Methods document available at osf.io/mu2ek.

## Supplementary Material

msaf182_Supplementary_Data

## Data Availability

Data and analysis code are archived with the Open Science Framework at osf.io/mu2ek. The full paper along with supplementary statistics and figures can be recreated using the quarto markdown files, containing all R code, MainTextCode.qmd and SuppStats.qmd. Raw data files are as follows: DataGuide.xlsx describes all variables in the data tables listed here Data File S1—Names for statistical models required to run MainTextCode.qmd Data File S2—All rifR mutations identified by Sanger sequencing the RRDR of *rpoB* Data File S3—Solid agar fluctuation assay results and intracellular ATP assay results Data File S4—Liquid fluctuation assay results for Δ*waaZ* and the wildtype Data File S5—Growth curve summary statistics for rifR mutants in the MG1655 background Data File S6—ΔΔG estimates for both *rpoB* and the RNAP complex under each given rifR mutation Data File S7—Fitness of *gyrA* mutants as measured by Harmand et al. (2017) and summarized by Gifford et al. (2018) Data File S8—Wildtype *gyrA* sequence required to run MainTextCode.qmd Data File S9—Codon table required to run MainTextCode.qmd Data File S10—Wildtype *folA* sequence required to run MainTextCode.qmd Data File S11—Results from illumina whole genome sequencing of *nudJ* knockout strains Data File S12—Raw growth curves and summary statistics for wildtype BW25113, Δ*nudJ* and Δ*nudJ* + IS1 *waaZ* Data File S13—Fitness of *folA* mutants as measured by Palmer et al. (2015) Data File S14—Raw data from fluctuation assays to Nal and Cyc markers

## References

[msaf182-B1] Alam M, Alhhazmi A, DeCoteau John F, Luo Y, Geyer CR. Reca inhibitors potentiate antibiotic activity and block evolution of antibiotic resistance. Cell Chem Biol. 2016:23(3):381–391. 10.1016/j.chembiol.2016.02.010. 26991103

[msaf182-B2] Aleixandre V, Urios A, Herrera G, Blanco M. New Escherichia coli gyrA and gyrB mutations which have a graded effect on DNA supercoiling. Mol Gen Genet. 1989:219(1-2):306–312. 10.1007/bf00261192. 2559316

[msaf182-B3] Baba T, Ara T, Hasegawa M, Takai Y, Okumura Y, Baba M, Datsenko KA, Tomita M, Wanner BL, Mori H. Construction of *Escherichia coli* K-12 in-frame, single-gene knockout mutants: the Keio collection. Mol Syst Biol. 2006:2(1):2006.0008. 10.1038/msb4100050. PMC1681482 16738554

[msaf182-B4] Barnhart MM, Chapman MR. Curli biogenesis and function. Annu Rev Microbiol. 2006:60(1):131–147. 10.1146/annurev.micro.60.080805.142106. 16704339 PMC2838481

[msaf182-B5] Bateman A, Martin M-J, Orchard S, Magrane M, Ahmad S, Alpi E, Bowler-Barnett EH, Britto R, Bye-A-Jee H, Cukura A, et al UniProt: the Universal Protein Knowledgebase in 2023. Nucleic Acids Res. 2023:51:D523–D531. 10.1093/nar/gkac1052. 36408920 PMC9825514

[msaf182-B6] Bergval IL, Klatser PR, Schuitema ARJ, Oskam L, Anthony RM. Specific mutations in the *Mycobacterium tuberculosis rpoB* gene are associated with increased *dnaE2*expression. FEMS Microbiol Lett. 2007:275(2):338–343. 10.1111/j.1574-6968.2007.00905.x. 17868360

[msaf182-B7] Bessman MJ . A cryptic activity in the Nudix hydrolase superfamily. Protein Sci. 2019:28(8):1494–1500. 10.1002/pro.3666. 31173659 PMC6635765

[msaf182-B8] Bessman MJ, Frick DN, O’Handley SF. The MutT proteins or “Nudix” hydrolases, a family of Versatile, widely distributed, “Housecleaning” enzymes. J Biol Chem. 1996:271(41):25059–25062. 10.1074/jbc.271.41.25059. 8810257

[msaf182-B9] Bohman K, Ruusala T, Jelenc PC, Kurland CG. Kinetic impairment of restrictive streptomycin-resistant ribosomes. Mol Gen Genet. 1984:198(1):90–99. 10.1007/bf00328706. 6394968

[msaf182-B10] Braffman N, Hauver J, Campbell EA, Darst SA. *Protein structure for* *Escherichia coli* RNA polymerase sigma70-holoenzyme bound to upstream fork promoter DNA. 2019. 10.2210/pdb6n62/pdb.

[msaf182-B11] Brauner A, Fridman O, Gefen O, Balaban NQ. Distinguishing between resistance, tolerance and persistence to antibiotic treatment. Nat Rev Microbiol. 2016:14(5):320–330. 10.1038/nrmicro.2016.34. 27080241

[msaf182-B12] Buckstein MH, He J, Rubin H. Characterization of nucleotide pools as a function of physiological state in *Escherichia coli*. J Bacteriol. 2008:190(2):718–726. 10.1128/jb.01020-07. 17965154 PMC2223692

[msaf182-B13] Callens M, Rose CJ, Finnegan M, Gatchitch F, Simon L, Hamet J, Pradier L, Dubois M-P, Bedhomme S. Hypermutator emergence in experimental *Escherichia coli* populations is stress-type dependent. Evol Lett. 2023:7(4):252–261. 10.1093/evlett/qrad019. 37475751 PMC10355175

[msaf182-B14] Cano AV, Rozhoňová H, Stoltzfus A, McCandlish DM, Payne JL. Mutation bias shapes the spectrum of adaptive substitutions. Proc Natl Acad Sci U S A. 2022:119(7):e2119720119. 10.1073/pnas.2119720119. 35145034 PMC8851560

[msaf182-B15] Caravagna G, Sanguinetti G, Graham TA, Sottoriva A. The MOBSTER R package for tumour subclonal deconvolution from bulk DNA whole-genome sequencing data. BMC Bioinformatics. 2020:21(1):531. 10.1186/s12859-020-03863-1. 33203356 PMC7672894

[msaf182-B16] Carvajal-Garcia J, Bracey H, Johnson AE, Hernandez Viera AJ, Egli M, Simsek EN, Jaremba EA, Kim K, Merrikh H. A small molecule that inhibits the evolution of antibiotic resistance. NAR Mol Med. 2024:1(1):ugae001. 10.1093/narmme/ugae001. 38911259 PMC11188740

[msaf182-B17] Carvajal-Garcia J, Samadpour AN, Hernandez Viera AJ, Merrikh H. Oxidative stress drives mutagenesis through transcription-coupled repair in bacteria. Proc Natl Acad Sci U S A. 2023:120(27):e2300761120. 10.1073/pnas.2300761120. 37364106 PMC10318952

[msaf182-B18] Chao K-H, Barton K, Palmer S, Lanfear R. Sangeranalyser: simple and interactive analysis of sanger sequencing data in r. Genome Biol Evol. 2021:13(3):evab028. 10.1093/gbe/evab028. 33591316 PMC7939931

[msaf182-B19] Cherry JL . T residues preceded by runs of G are hotspots of T→G mutation in Bacteria. Genome Biol Evol. 2023:15(6):evad087. 10.1093/gbe/evad087. 37216188 PMC10243904

[msaf182-B20] Chou HH, Keasling JD. Synthetic pathway for production of five-carbon alcohols from isopentenyl diphosphate. Appl Environ Microbiol. 2012:78(22):7849–7855. 10.1128/aem.01175-12. 22941086 PMC3485928

[msaf182-B21] Chu X-L, Zhang B-W, Zhang Q-G, Zhu B-R, Lin K, Zhang D-Y. Temperature responses of mutation rate and mutational spectrum in an *Escherichia coli* strain and the correlation with metabolic rate. BMC Evol Biol. 2018:18(1):126. 10.1186/s12862-018-1252-8. 30157765 PMC6116381

[msaf182-B22] Cirz RT, Chin JK, Andes DR, de Crécy-Lagard V, Craig WA, Romesberg FE. Inhibition of mutation and combating the evolution of antibiotic resistance. PLoS Biol. 2005:3(6):e176. 10.1371/journal.pbio.0030176. 15869329 PMC1088971

[msaf182-B23] Couce A, Alonso-Rodriguez N, Costas C, Oliver A, Blázquez J. Intrapopulation variability in mutator prevalence among urinary tract infection isolates of *Escherichia coli*. Clin Microbiol Infect. 2016:22(6):566.e1–566.e7. 10.1016/j.cmi.2016.03.008. 27021422

[msaf182-B24] Couce A, Guelfo JR, Blázquez J. Mutational Spectrum drives the rise of mutator Bacteria. PLoS Genet. 2013:9(1):e1003167. 10.1371/journal.pgen.1003167. 23326242 PMC3542065

[msaf182-B25] Couce A, Rodríguez-Rojas A, Blázquez J. Bypass of genetic constraints during mutator evolution to antibiotic resistance. Proc R Soc Lond B Biol Sci. 2015:282(1804):20142698. 10.1098/rspb.2014.2698. PMC4375862 25716795

[msaf182-B26] Couce A, Tenaillon O. Mutation bias and GC content shape antimutator invasions. Nat Commun. 2019:10(1):3114. 10.1038/s41467-019-11217-6. 31308380 PMC6629674

[msaf182-B27] Csörgő B, Fehér T, Tímár E, Blattner FR, Pósfai G. Low-mutation-rate, reduced-genome *Escherichia coli*: an improved host for faithful maintenance of engineered genetic constructs. Microb Cell Fact. 2012:11(1):11. 10.1186/1475-2859-11-11. 22264280 PMC3280934

[msaf182-B28] Deatherage DE, Barrick JE. Identification of mutations in laboratory-evolved microbes from next-generation sequencing data using breseq. Methods Mol Biol. 2014:1151:165–188. 10.1007/978-1-4939-0554-6_12. 24838886 PMC4239701

[msaf182-B29] Deatherage DE, Leon D, Rodriguez ”E, Omar SK, Barrick JE. Directed evolution of *Escherichia coli* with lower-than-natural plasmid mutation rates. Nucleic Acids Res. 2018:46(17):9236–9250. 10.1093/nar/gky751. 30137492 PMC6158703

[msaf182-B30] de Visser JAGM, Zeyl CW, Gerrish PJ, Blanchard JL, Lenski RE. Diminishing returns from mutation supply rate in asexual populations. Science. 1999:283(5400):404–406. 10.1126/science.283.5400.404. 9888858

[msaf182-B31] Domenech A, Brochado AR, Sender V, Hentrich K, Henriques-Normark B, Typas A, Veening J-W. Proton motive force disruptors block bacterial competence and horizontal gene transfer. Cell Host Microbe. 2020:27(4):544–555.e3. 10.1016/j.chom.2020.02.002. 32130952

[msaf182-B32] Drake JW . General antimutators are improbable. J Mol Biol. 1993:229(1):8–13. 10.1006/jmbi.1993.1002. 8421317

[msaf182-B33] Elgrail MM, Sprouffske K, Dartey JO, Garcia AM. Emergence of a multilocus mutator genotype in mutator *Escherichia coli* experimental populations under repeated lethal selection. J Evol Biol. 2024:37(3):346–352. 10.1093/jeb/voae007. 38367184

[msaf182-B34] Fijalkowska IJ, Dunn RL, Schaaper RM. Mutants of *Escherichia coli* with increased fidelity of DNA replication. Genetics. 1993:134(4):1023–1030. 10.1093/genetics/134.4.1023. 8375645 PMC1205570

[msaf182-B35] Fitzsimmons WJ, Woods RJ, McCrone JT, Woodman A, Arnold JJ, Yennawar M, Evans R, Cameron CE, Lauring AS. A speed fidelity trade-off determines the mutation rate and virulence of an RNA virus. PLoS Biol. 2018:16(6):e2006459. 10.1371/journal.pbio.2006459. 29953453 PMC6040757

[msaf182-B36] Foster PL . Methods for determining spontaneous mutation rates. Methods Enzymol. 2006:409:195–213. 10.1016/s0076-6879(05)09012-9. 16793403 PMC2041832

[msaf182-B37] Foster PL, Lee H, Popodi E, Townes JP, Tang H. Determinants of spontaneous mutation in the bacterium *Escherichia coli* as revealed by whole-genome sequencing. Proc Natl Acad Sci U S A. 2015:112(44):E5990–E5999. 10.1073/pnas.1512136112. 26460006 PMC4640725

[msaf182-B38] Fowler RG, Erickson JA, Isbell RJ. Activity of the *Escherichia coli mutT* mutator allele in an anaerobic environment. J Bacteriol. 1994:176(24):7727–7729. 10.1128/jb.176.24.7727-7729.1994. 8002599 PMC197232

[msaf182-B39] Frirdich E, Lindner B, Holst O, Whitfield C. Overexpression of the *waaZ* gene leads to modification of the structure of the inner core region of *Escherichia coli* lipopolysaccharide, truncation of the outer core, and reduction of the amount of O polysaccharide on the cell surface. J Bacteriol. 2003:185(5):1659–1671. 10.1128/jb.185.5.1659-1671.2003. 12591884 PMC148070

[msaf182-B40] Fujisaki S, Nishino T, Katsuki H. Isoprenoid synthesis in *Escherichia coli*. Separation and partial purification of four enzymes involved in the synthesis 1. J Biochem. 1986:99(5):1327–1337. 10.1093/oxfordjournals.jbchem.a135600. 3519603

[msaf182-B41] Fujisaki S, Takahashi I, Hara H, Horiuchi K, Nishino T, Nishimura Y. Disruption of the structural gene for farnesyl diphosphate synthase in *Escherichia coli*. J Biochem. 2005:137(3):395–400. 10.1093/jb/mvi049. 15809342

[msaf182-B42] Furuyama TN, Guedes de Carvalho IMV, Janini LMR, Antoneli F. Enlarging viral mutation estimation: A view from the distribution of mutation rates. bioRxiv 644362 10.1101/2025.03.20.644362. 20 March 2025, preprint: not peer reviewed PMC13465770 42584925

[msaf182-B43] Fusco D, Gralka M, Kayser J, Anderson A, Hallatschek O. Excess of mutational jackpot events in expanding populations revealed by spatial LuriaDelbrück experiments. Nat Commun. 2016:7(1):12760. 10.1038/ncomms12760. 27694797 PMC5059437

[msaf182-B44] Garibyan L . Use of the *rpoB* gene to determine the specificity of base substitution mutations on the *Escherichia coli* chromosome. DNA Repair (Amst). 2003:2(5):593–608. 10.1016/s1568-7864(03)00024-7. 12713816

[msaf182-B45] Garushyants SK, Sane M, Selifanova MV, Agashe D, Bazykin GA, Gelfand MS. Mutational signatures in wild type *Escherichia coli* strains reveal predominance of DNA polymerase errors. Genome Biol Evol. 2024:16(4):evae035. 10.1093/gbe/evae035. 38401265 PMC10995721

[msaf182-B46] Geiger JR, Speyer JF. A conditional antimutator in *E. coli*. Mol Gen Genet. 1977:153(1):87–97. 10.1007/bf01036000. 329107

[msaf182-B47] Gifford DR, Berríos-Caro E, Joerres C, Suñé M, Forsyth JH, Bhattacharyya A, Galla T, Knight CG. Mutators can drive the evolution of multi-resistance to antibiotics. PLoS Genet. 2023:19(6):e1010791. 10.1371/journal.pgen.1010791. 37311005 PMC10292718

[msaf182-B48] Gifford DR, Bhattacharyya A, Geim A, Marshall E, Krašovec R, Knight CG. Environmental and genetic influence on the rate and spectrum of spontaneous mutations in *Escherichia coli*. Microbiology. 2024:170(4):001452. 10.1099/mic.0.001452. 38687010 PMC11084559

[msaf182-B49] Gifford DR, Krašovec R, Aston E, Belavkin RV, Channon A, Knight CG. Environmental pleiotropy and demographic history direct adaptation under antibiotic selection. Heredity (Edinb). 2018:121(5):438–448. 10.1038/s41437-018-0137-3. 30190561 PMC6180006

[msaf182-B50] Gifford DR, Moss E, MacLean RC. Environmental variation alters the fitness effects of rifampicin resistance mutations in *Pseudomonas aeruginosa*. Evolution. 2016:70(3):725–730. 10.1111/evo.12880. 26880677

[msaf182-B51] Gon S, Napolitano R, Rocha W, Coulon S, Fuchs RP. Increase in dNTP pool size during the DNA damage response plays a key role in spontaneous and induced-mutagenesis in *Escherichia coli*. Proc Natl Acad Sci U S A. 2011:108(48):19311–19316. 10.1073/pnas.1113664108. 22084087 PMC3228436

[msaf182-B52] Good BH, McDonald MJ, Barrick JE, Lenski RE, Desai MM. The dynamics of molecular evolution over 60,000 generations. Nature. 2017:551(7678):45–50. 10.1038/nature24287. 29045390 PMC5788700

[msaf182-B53] Goyal P, Krasteva PV, Van Gerven N, Gubellini F, Van den Broeck I, Troupiotis-Tsaïlaki A, Jonckheere W, Péhau-Arnaudet G, Pinkner JS, Chapman MR, et al Structural and mechanistic insights into the bacterial amyloid secretion channel CsgG. Nature. 2014:516(7530):250–253. 10.1038/nature13768. 25219853 PMC4268158

[msaf182-B54] Green R, Bawn M, Angus-Whiteoak A, Jago M, Whelan FJ, Lagator M, Hall N, Krasovec R, Knight CG. Identifying rare spontaneous mutations through wildtype *E. coli* population sequencing. bioRxiv 663732. 10.1101/2025.07.08.663732., 12 July 2025, preprint: not peer reviewed.

[msaf182-B55] Green R, Wang H, Botchey C, Zhang SNN, Wadsworth C, Tyrrell F, Letton J, McBain AJ, Paszek P, Krašovec R, et al Collective peroxide detoxification determines microbial mutation rate plasticity in *E. coli*. PLoS Biol. 2024:22(7):e3002711. 10.1371/journal.pbio.3002711. 39008532 PMC11272383

[msaf182-B56] Grenier F, Matteau D, Baby V, Rodrigue S. Complete genome sequence of *Escherichia coli* BW25113. Genome Announc. 2014:2(5):e01038-14. 10.1128/genomea.01038-14. 25323716 PMC4200154

[msaf182-B57] Gudkov SV, Shtarkman IN, Smirnova VS, Chernikov AV, Bruskov VI. Guanosine and inosine as natural antioxidants and radioprotectors for mice exposed to lethal doses of γ-radiation. Dokl Biochem Biophys. 2006:407(1):47–50. 10.1134/s1607672906020013. 16776063

[msaf182-B58] Gulmezian M, Hyman KR, Marbois BN, Clarke CF, Javor GT. The role of UbiX in Escherichia coli coenzyme Q biosynthesis. Arch Biochem Biophys. 2007:467(2):144–153. 10.1016/j.abb.2007.08.009. 17889824 PMC2475804

[msaf182-B59] Guo Z, Feng S, Liang L, Wu Z, Min L, Wang R, Li J, Zhong L-L, Zhao H, Chen X, et al Assessment of the reversibility of resistance in the absence of antibiotics and its relationship with the resistance gene's fitness cost: a genetic study with mcr-1. Lancet Microbe. 2024:5(8):100846. 10.1016/s2666-5247(24)00052-1. 38870982

[msaf182-B60] Hall AR . Genotype-by-environment interactions due to antibiotic resistance and adaptation in *Escherichia coli*. J Evol Biol. 2013:26(8):1655–1664. 10.1111/jeb.12172. 23701170

[msaf182-B61] Harmand N, Gallet R, Jabbour-Zahab R, Martin G, Lenormand T. Fisher's geometrical model and the mutational patterns of antibiotic resistance across dose gradients. Evolution. 2017:71(1):23–37. 10.1111/evo.13111. 27805262

[msaf182-B62] Ho W-C, Behringer MG, Miller SF, Gonzales J, Nguyen A, Allahwerdy M, Boyer GF, Lynch M. Evolutionary dynamics of asexual hypermutators adapting to a novel environment. Genome Biol Evol. 2021:13(12):evab257. 10.1093/gbe/evab257. 34864972 PMC8643662

[msaf182-B63] Hori M, Asanuma T, Inanami O, Kuwabara M, Harashima H, Kamiya H. Effects of overexpression and antisense RNA expression of Orf17, a MutT-type enzyme. Biol Pharm Bull. 2006:29(6):1087–1091. 10.1248/bpb.29.1087. 16754998

[msaf182-B64] Horton JS, Taylor TB. Mutation bias and adaptation in bacteria. Microbiology. 2023:169(11):001404. 10.1099/mic.0.001404. 37943288 PMC10710837

[msaf182-B65] Jago MJ, Soley JK, Denisov S, Walsh CJ, Gifford DR, Howden BP, Lagator M. High-throughput method characterizes hundreds of previously unknown antibiotic resistance mutations. Nat Commun. 2025:16(1):780. 10.1038/s41467-025-56050-2. 39824824 PMC11742677

[msaf182-B66] Jubelin G, Vianney A, Beloin C, Ghigo J-M, Lazzaroni J-C, Lejeune P, Dorel C. Cpxr/OmpR interplay regulates curli gene expression in response to osmolarity in *Escherichia coli*. J Bacteriol. 2005:187(6):2038–2049. 10.1128/jb.187.6.2038-2049.2005. 15743952 PMC1064031

[msaf182-B67] Jumper J, Evans R, Pritzel A, Green T, Figurnov M, Ronneberger O, Tunyasuvunakool K, Bates R, Žídek A, Potapenko A, et al Highly accurate protein structure prediction with AlphaFold. Nature. 2021:596(7873):583–589. 10.1038/s41586-021-03819-2. 34265844 PMC8371605

[msaf182-B68] Kamiya H, Iida E, Murata-Kamiya N, Yamamoto Y, Miki T, Harashima H. Suppression of spontaneous and hydrogen peroxide-induced mutations by a MutT-type nucleotide pool sanitization enzyme, the *Escherichia coli* Orf135 protein. Genes Cells. 2003:8(12):941–950. 10.1046/j.1365-2443.2003.00688.x. 14750949

[msaf182-B69] Kanai Y, Tsuru S, Furusawa C. Experimental demonstration of operon formation catalyzed by insertion sequence. Nucleic Acids Res. 2022:50(3):1673–1686. 10.1093/nar/gkac004. 35066585 PMC8860574

[msaf182-B70] Krašovec R, Belavkin RV, Aston JAD, Channon A, Aston E, Rash BM, Kadirvel M, Forbes S, Knight CG. Mutation rate plasticity in rifampicin resistance depends on *Escherichia coli* cellcell interactions. Nat Commun. 2014:5(1):3742. 10.1038/ncomms4742. 24776982 PMC4007418

[msaf182-B71] Krašovec R, Richards H, Gifford DR, Belavkin RV, Channon A, Aston E, McBain AJ, Knight CG. Opposing effects of final population density and stress on *Escherichia coli* mutation rate. ISME J. 2018:12(12):2981–2987. 10.1038/s41396-018-0237-3. 30087411 PMC6230470

[msaf182-B72] Krašovec R, Richards H, Gifford DR, Hatcher C, Faulkner KJ, Belavkin RV, Channon A, Aston E, McBain AJ, Knight CG. Spontaneous mutation rate is a plastic trait associated with population density across domains of life. PLoS Biol. 2017:15(8):e2002731. 10.1371/journal.pbio.2002731. 28837573 PMC5570273

[msaf182-B73] Krašovec R, Richards H, Gomez G, Gifford DR, Mazoyer A, Knight CG. Measuring microbial mutation rates with the fluctuation assay. J Vis Exp. 2019:153:e60406. 10.3791/60406. 31840662

[msaf182-B74] Kunz BA, Glickman BW. Mechanism of mutation by thymine starvation in *Escherichia coli*: clues from mutagenic specificity. J Bacteriol. 1985:162(3):859–864. 10.1128/jb.162.3.859-864.1985. 3888966 PMC215854

[msaf182-B75] Lagage V, Chen V, Uphoff S. Adaptation delay causes a burst of mutations in bacteria responding to oxidative stress. EMBO Rep. 2023:24(1):e55640. 10.15252/embr.202255640. 36397732 PMC9827559

[msaf182-B76] Lawhorn BG, Gerdes SY, Begley TP. A genetic screen for the identification of thiamin metabolic genes. J Biol Chem. 2004:279(42):43555–43559. 10.1074/jbc.m404284200. 15292217

[msaf182-B77] Lee H, Popodi E, Tang H, Foster PL. Rate and molecular spectrum of spontaneous mutations in the bacterium *Escherichia coli* as determined by whole-genome sequencing. Proc Natl Acad Sci U S A. 2012:109(41):E2774–E2783. 10.1073/pnas.1210309109. 22991466 PMC3478608

[msaf182-B78] Lee J-H, Lee K-L, Yeo W-S, Park S-J, Roe J-H. SoxRS-mediated lipopolysaccharide modification enhances resistance against multiple drugs in *Escherichia coli*. J Bacteriol. 2009:191(13):4441–4450. 10.1128/jb.01474-08. 19376854 PMC2698492

[msaf182-B79] Liu H, Zhang J. Yeast spontaneous mutation rate and Spectrum vary with environment. Curr Biol. 2019:29(10):1584–1591.e3. 10.1016/j.cub.2019.03.054. 31056389 PMC6529271

[msaf182-B80] Liu H, Zhang J. The rate and molecular spectrum of mutation are selectively maintained in yeast. Nat Commun. 2021:12(1):4044. 10.1038/s41467-021-24364-6. 34193872 PMC8245649

[msaf182-B81] Liu Q, Zhu J, Dulberger CL, Stanley S, Wilson S, Chung ES, Wang X, Culviner P, Liu YJ, Hicks ND, et al Tuberculosis treatment failure associated with evolution of antibiotic resilience. Science. 2022:378(6624):1111–1118. 10.1126/science.abq2787. 36480634 PMC9968493

[msaf182-B82] Long CP, Gonzalez JE, Feist AM, Palsson BO, Antoniewicz MR. Fast growth phenotype of E. coli K-12 from adaptive laboratory evolution does not require intracellular flux rewiring. Metab Eng. 2017:44:100–107. 10.1016/j.ymben.2017.09.012. 28951266 PMC5845443

[msaf182-B83] MacLean RC, Perron GG, Gardner A. Diminishing returns from beneficial mutations and pervasive epistasis shape the fitness landscape for rifampicin resistance in *Pseudomonas aeruginosa*. Genetics. 2010:186(4):1345–1354. 10.1534/genetics.110.123083. 20876562 PMC2998316

[msaf182-B84] Maharjan R, Ferenci T. Mutational signatures indicative of environmental stress in Bacteria. Mol Biol Evol. 2015:32(2):380–391. 10.1093/molbev/msu306. 25389207

[msaf182-B85] Maharjan R, Ferenci T. The fitness costs and benefits of antibiotic resistance in drug-free microenvironments encountered in the human body. Environ Microbiol Rep. 2017:9(5):635–641. 10.1111/1758-2229.12564. 28677342

[msaf182-B86] Maharjan RP, Ferenci T. The impact of growth rate and environmental factors on mutation rates and spectra in *Escherichia coli*. Environ Microbiol Rep. 2018:10(6):626–633. 10.1111/1758-2229.12661. 29797781

[msaf182-B87] Mahilkar A, Raj N, Kemkar S, Saini S. Selection in a growing colony biases results of mutation accumulation experiments. Sci Rep. 2022:12(1):15470. 10.1038/s41598-022-19928-5. 36104390 PMC9475022

[msaf182-B88] Maki H, Sekiguchi M. Mutt protein specifically hydrolyses a potent mutagenic substrate for DNA synthesis. Nature. 1992:355(6357):273–275. 10.1038/355273a0. 1309939

[msaf182-B89] Mazoyer A, Drouilhet R, Despreaux S, Ycart B. flan: An R Package for Inference on Mutation Models. The R Journal. 2017:9(1):334–351. 10.32614/RJ-2017-029. Flan: FLuctuation ANalysis on mutation models.

[msaf182-B90] McDonald MJ, Hsieh Y-Y, Yu Y-H, Chang S-L, Leu J-Y. The evolution of low mutation rates in experimental mutator populations of saccharomyces cerevisiae. Curr Biol. 2012:22(13):1235–1240. 10.1016/j.cub.2012.04.056. 22727704

[msaf182-B91] McLennan AG . The Nudix hydrolase superfamily. Cell Mol Life Sci. 2006:63(2):123–143. 10.1007/s00018-005-5386-7. 16378245 PMC11136074

[msaf182-B92] McLennan AG . Substrate ambiguity among the nudix hydrolases: biologically significant, evolutionary remnant, or both? Cell Mol Life Sci. 2013:70(3):373–385. 10.1007/s00018-012-1210-3. 23184251 PMC11113851

[msaf182-B93] Mizote T, Nakayama H. The *thiM* locus and its relation to phosphorylation of hydroxyethylthiazole in *Escherichia coli*. J Bacteriol. 1989:171(6):3228–3232. 10.1128/jb.171.6.3228-3232.1989. 2542220 PMC210041

[msaf182-B94] Novick A, Szilard L. Experiments with the chemostat on spontaneous mutations of Bacteria. Proc Natl Acad Sci U S A. 1950:36(12):708–719. 10.1073/pnas.36.12.708. 14808160 PMC1063276

[msaf182-B95] Novick A, Szilard L. Anti-mutagens. Nature. 1952:170(4335):926–927. 10.1038/170926a0. 13013261

[msaf182-B96] Nunoshiba T . A novel Nudix hydrolase for oxidized purine nucleoside triphosphates encoded by ORFYLR151c (PCD1 gene) in *Saccharomyces cerevisiae*. Nucleic Acids Res. 2004:32(18):5339–5348. 10.1093/nar/gkh868. 15475388 PMC524280

[msaf182-B97] Oliver A, Cantoń R, Campo P, Baquero F, Blaźquez J. High frequency of hypermutable *Pseudomonas aeruginosa* in cystic fibrosis lung infection. Science. 2000:288(5469):1251–1253. 10.1126/science.288.5469.1251. 10818002

[msaf182-B98] Oller AR, Schaaper RM. Spontaneous mutation in *Escherichia coli* containing the *dnaE911* DNA polymerase antimutator allele. Genetics. 1994:138(2):263–270. 10.1093/genetics/138.2.263. 7828810 PMC1206145

[msaf182-B99] Ovchinnikov YA, Monastyrskaya GS, Gubanov VV, Guryev SO, Chertov O, Modyanov NN, Grinkevich VA, Makarova IA, Marchenko TV, Polovnikova IN, et al The primary structure of *Escherichia coli* RNA polymerase. Eur J Biochem. 1981:116(3):621–629. 10.1111/j.1432-1033.1981.tb05381.x. 6266829

[msaf182-B100] Palmer AC, Toprak E, Baym M, Kim S, Veres A, Bershtein S, Kishony R. Delayed commitment to evolutionary fate in antibiotic resistance fitness landscapes. Nat Commun. 2015:6(1):7385. 10.1038/ncomms8385. 26060115 PMC4548896

[msaf182-B101] Payne KAP, White MD, Fisher K, Khara B, Bailey SS, Parker D, Rattray NJW, Trivedi DK, Goodacre R, Beveridge R, et al New cofactor supports α, β-unsaturated acid decarboxylation via 1,3-dipolar cycloaddition. Nature. 2015:522(7557):497–501. 10.1038/nature14560. 26083754 PMC4988494

[msaf182-B102] Pośfai G, Plunkett G, Feheŕ T, Frisch D, Keil GM, Umenhoffer K, Kolisnychenko V, Stahl B, Sharma SS, de Arruda M, et al Emergent properties of reduced-genome *Escherichia coli*. Science. 2006:312(5776):1044–1046. 10.1126/science.1126439. 16645050

[msaf182-B103] Ragheb MN, Merrikh C, Browning K, Merrikh H. Mfd regulates RNA polymerase association with hard-to-transcribe regions in vivo, especially those with structured RNAs. Proc Natl Acad Sci U S A. 2021:118(1):e2008498118. 10.1073/pnas.2008498118. 33443179 PMC7817204

[msaf182-B104] Ragheb MN, Thomason MK, Hsu C, Nugent P, Gage J, Samadpour AN, Kariisa A, Merrikh CN, Miller SI, Sherman DR, et al Inhibiting the evolution of antibiotic resistance. Mol Cell. 2019:73(1):157–165.e5. 10.1016/j.molcel.2018.10.015. 30449724 PMC6320318

[msaf182-B105] R Core Team . R: A Language and Environment for Statistical Computing. R Foundation for Statistical Computing, Vienna, Austria. 2023. https://www.R-project.org.

[msaf182-B106] Saint-Ruf C, Pesut J, Sopta M, Matic I. Causes and consequences of DNA repair activity modulation during stationary phase in *Escherichia coli*. Crit Rev Biochem Mol Biol. 2007:42(4):259–270. 10.1080/10409230701495599. 17687668

[msaf182-B107] Sane M, Diwan GD, Bhat BA, Wahl LM, Agashe D. Shifts in mutation spectra enhance access to beneficial mutations. Proc Natl Acad Sci U S A. 2023:120(22):e2207355120. 10.1073/pnas.2207355120. 37216547 PMC10235995

[msaf182-B108] Sane M, Parveen S, Agashe D. Mutation bias alters the distribution of fitness effects of mutations. PLoS Biol. 2025:23(7):e3003282. 10.1371/journal.pbio.3003282. 40658723 PMC12273949

[msaf182-B109] ScarabGenomics . 2024. Clean Genome® LowMut—Scarab Genomics. scarabgenomics.com. https://www.scarabgenomics.com/product/clean-genome-lowmut/[Accessed 2024-November-4]

[msaf182-B110] Schaaper RM . The mutational specificity of two *Escherichia coli dnaE* antimutator alleles as determined from *lacI* mutation spectra. Genetics. 1993:134(4):1031–1038. 10.1093/genetics/134.4.1031. 8375646 PMC1205571

[msaf182-B111] Schaaper RM . Suppressors of *Escherichia coli mutT*: antimutators for DNA replication errors. Mutat Res. 1996:350(1):17–23. 10.1016/0027-5107(95)00086-0. 8657178

[msaf182-B112] Schaaper RM, Dunn RL. The antimutator phenotype of *E. coli mud* is only apparent and results from delayed appearance of mutants. Mutat Res. 2001:480-481:71–75. 10.1016/s0027-5107(01)00170-1. 11506800

[msaf182-B113] Schreck CF, Fusco D, Karita Y, Martis S, Kayser J, Duvernoy M-C, Hallatschek O. Impact of crowding on the diversity of expanding populations. Proc Natl Acad Sci U S A. 2023:120(11):e2208361120. 10.1073/pnas.2208361120. 36881622 PMC10089160

[msaf182-B114] Schymkowitz J, Borg J, Stricher F, Nys R, Rousseau F, Serrano L. The FoldX web server: an online force field. Nucleic Acids Res. 2005:33(Web Server):W382–W388. 10.1093/nar/gki387. 15980494 PMC1160148

[msaf182-B115] Selby CP, Witkin EM, Sancar A. *Escherichia coli mfd* mutant deficient in “mutation frequency decline” lacks strand-specific repair: in vitro complementation with purified coupling factor. Proc Natl Acad Sci U S A. 1991:88(24):11574–11578. 10.1073/pnas.88.24.11574. 1763073 PMC53178

[msaf182-B116] Shepherd MJ, Horton JS, Taylor TB. A near-deterministic mutational hotspot in *Pseudomonas fluorescens* is constructed by multiple interacting genomic features. Mol Biol Evol. 2022:39(6):msac132. 10.1093/molbev/msac132. 35707979 PMC9234803

[msaf182-B117] Sloan DB, Broz AK, Sharbrough J, Wu Z. Detecting rare mutations and DNA damage with sequencing-based methods. Trends Biotechnol. 2018:36(7):729–740. 10.1016/j.tibtech.2018.02.009. 29550161 PMC6004327

[msaf182-B118] Smith D, Price J, Burby P, Blanco L, Chamberlain J, Chapman M. The production of curli amyloid fibers is deeply integrated into the biology of *Escherichia coli*. Biomolecules. 2017:7(4):75. 10.3390/biom7040075. 29088115 PMC5745457

[msaf182-B119] Søballe B, Poole RK. Ubiquinone limits oxidative stress in *Escherichia coli*. Microbiology. 2000:146(4):787–796. 10.1099/00221287-146-4-787. 10784036

[msaf182-B120] Soley JK, Jago M, Walsh CJ, Khomarbaghi Z, Howden BP, Lagator M. Pervasive genotype-by-environment interactions shape the fitness effects of antibiotic resistance mutations. Proc R Soc Lond B Biol Sci. 2023:290(2005):20231030. 10.1098/rspb.2023.1030. PMC10427823 37583318

[msaf182-B121] Sprouffske K, Aguilar-Rodríguez J, Sniegowski P, Wagner A. High mutation rates limit evolutionary adaptation in *Escherichia coli*. PLoS Genet. 2018:14(4):e1007324. 10.1371/journal.pgen.1007324. 29702649 PMC5942850

[msaf182-B122] Sprouffske K, Wagner A. Growthcurver: an R package for obtaining interpretable metrics from microbial growth curves. BMC Bioinformatics. 2016:17:172. 10.1186/s12859-016-1016-7. Growthcurver: Simple metrics to summarize growth curves. 27094401 PMC4837600

[msaf182-B123] Sun TA, Lind PA. Distribution of mutation rates challenges evolutionary predictability. Microbiology. 2023:169(5):001323. 10.1099/mic.0.001323. 37134005 PMC10268835

[msaf182-B124] Sung W, Ackerman MS, Miller SF, Doak TG, Lynch M. Drift-barrier hypothesis and mutation-rate evolution. Proc Natl Acad Sci U S A. 2012:109(45):18488–18492. 10.1073/pnas.1216223109. 23077252 PMC3494944

[msaf182-B125] Swings T, Van den Bergh B, Wuyts S, Oeyen E, Voordeckers K, Verstrepen KJ, Fauvart M, Verstraeten N, Michiels J. Adaptive tuning of mutation rates allows fast response to lethal stress in *Escherichia coli*. eLife. 2017:6:e22939. 10.7554/elife.22939. 28460660 PMC5429094

[msaf182-B126] Szklarczyk D, Kirsch R, Koutrouli M, Nastou K, Mehryary F, Hachilif R, Gable AL, Fang T, Doncheva N, Pyysalo S, et al The STRING database in 2023: proteinprotein association networks and functional enrichment analyses for any sequenced genome of interest. Nucleic Acids Res. 2022:51(D1):D638–D646. 10.1093/nar/gkac1000. PMC9825434 36370105

[msaf182-B127] Tajiri T, Maki H, Sekiguchi M. Functional cooperation of MutT, MutM and MutY proteins in preventing mutations caused by spontaneous oxidation of guanine nucleotide in *Escherichia coli*. Mutat Res. 1995:336(3):257–267. 10.1016/0921-8777(94)00062-b. 7739614

[msaf182-B128] Treffers HP, Spinelli V, Belser NO. A factor (or mutator gene) influencing mutation rates in *Escherichia Coli*. Proc Natl Acad Sci U S A. 1954:40(11):1064–1071. 10.1073/pnas.40.11.1064. 16578437 PMC1063964

[msaf182-B129] Tsuzuki T, Egashira A, Igarashi H, Iwakuma T, Nakatsuru Y, Tominaga Y, Kawate H, Nakao K, Nakamura K, Ide F, et al Spontaneous tumorigenesis in mice defective in the *MTH1* gene encoding 8-oxo-dGTPase. Proc Natl Acad Sci U S A. 2001:98(20):11456–11461. 10.1073/pnas.191086798. 11572992 PMC58751

[msaf182-B130] Tuffaha MZ, Varakunan S, Castellano D, Gutenkunst RN, Wahl LM. Shifts in mutation bias promote mutators by altering the distribution of fitness effects. Am Nat. 2023:202(4):503–518. 10.1086/726010. 37792927 PMC11288183

[msaf182-B131] Umenhoffer K, Fehér T, Balikó G, Ayaydin F, Pósfai J, Blattner FR, Pósfai G. Reduced evolvability of *Escherichia coli* MDS42, an IS-less cellular chassis for molecular and synthetic biology applications. Microb Cell Fact. 2010:9(1):38. 10.1186/1475-2859-9-38. 20492662 PMC2891674

[msaf182-B132] Varadi M, Bertoni D, Magana P, Paramval U, Pidruchna I, Radhakrishnan M, Tsenkov M, Nair S, Mirdita M, Yeo J, et al AlphaFold protein structure database in 2024: providing structure coverage for over 214 million protein sequences. Nucleic Acids Res. 2024:52(D1):D368–D375. 10.1093/nar/gkad1011. 37933859 PMC10767828

[msaf182-B133] Varik V, Oliveira SRA, Hauryliuk V, Tenson T. HPLC-based quantification of bacterial housekeeping nucleotides and alarmone messengers ppGpp and pppGpp. Sci Rep. 2017:7(1):11022. 10.1038/s41598-017-10988-6. 28887466 PMC5591245

[msaf182-B134] Vogwill T, MacLean RC. The genetic basis of the fitness costs of antimicrobial resistance: a meta-analysis approach. Evol Appl. 2015:8(3):284–295. 10.1111/eva.12202. 25861386 PMC4380922

[msaf182-B135] Wahl LM, Agashe D. Selection bias in mutation accumulation. Evolution. 2022:76(3):528–540. 10.1111/evo.14430. 34989408

[msaf182-B136] Wang J, Ma W, Wang X. Insights into the structure of *Escherichia coli* outer membrane as the target for engineering microbial cell factories. Microb Cell Fact. 2021:20(1):73. 10.1186/s12934-021-01565-8. 33743682 PMC7980664

[msaf182-B137] Wang P-H, Khusnutdinova AN, Luo F, Xiao J, Nemr K, Flick R, Brown G, Mahadevan R, Edwards EA, Yakunin AF. Biosynthesis and activity of prenylated FMN cofactors. Cell Chem Biol. 2018:25(5):560–570.e6. 10.1016/j.chembiol.2018.02.007. 29551348

[msaf182-B138] Wielgoss S, Barrick JE, Tenaillon O, Wiser MJ, Dittmar WJ, Cruveiller S, Chane-Woon-Ming B, Médigue C, Lenski RE, Schneider D. Mutation rate dynamics in a bacterial population reflect tension between adaptation and genetic load. Proc Natl Acad Sci U S A. 2013:110(1):222–227. 10.1073/pnas.1219574110. 23248287 PMC3538217

[msaf182-B139] Wolff E, Kim M, Hu K, Yang H, Miller JH. Polymerases leave fingerprints: analysis of the mutational Spectrum in *Escherichia coli rpoB* to assess the role of polymerase IV in spontaneous mutation. J Bacteriol. 2004:186(9):2900–2905. 10.1128/jb.186.9.2900-2905.2004. 15090533 PMC387785

[msaf182-B140] Xu W, Dunn CA, O’Handley SF, Smith DL, Bessman MJ. Three new nudix hydrolases from *Escherichia coli*. J Biol Chem. 2006:281(32):22794–22798. 10.1074/jbc.m603407200. 16766526

[msaf182-B141] Yamagishi J, Yoshida H, Yamayoshi M, Nakamura S. Nalidixic acid-resistant mutations of the *gyrB* gene of *Escherichia coli*. Mol Gen Genet. 1986:204(3):367–373. 10.1007/BF00331012. 3020376

[msaf182-B142] Yampolsky LY, Stoltzfus A. Bias in the introduction of variation as an orienting factor in evolution. Evol Dev. 2001:3(2):73–83. 10.1046/j.1525-142x.2001.003002073.x. 11341676

[msaf182-B143] Zhang Y, Morar M, Ealick SE. Structural biology of the purine biosynthetic pathway. Cell Mol Life Sci. 2008:65(23):3699–3724. 10.1007/s00018-008-8295-8. 18712276 PMC2596281

