# Supplementary material for: Antimutator and Mutational Spectrum Effects Can Combine to Reduce Evolutionary Potential in Escherichia coli ΔnudJ
Source: Mol Biol Evol. 2025 Jul 29;42(8):msaf182. doi: 10.1093/molbev/msaf182 (PMC12359138; doi:10.1093/molbev/msaf182)
Supplement: msaf182_Supplementary_Data [file msaf182_supplementary_data.zip › MainText_Rev2_ONLY_SUPPLEMENT_WITH_REFERENCES.pdf]

Supplementary Materials (Text, Figures & Tables) for Green et al, 2025

Supplementary Text: RifR mutant fitness is correlated with the destabilising effect of mutations

To test the basis for the variation among the different DFE_{β}, we asked what explanatory factor could be determining the fitness of individual *rpoB* mutants. One potential cause of mutant fitness variation is the change in protein stability caused by the change to the AA sequence. Previous evidence suggests that mutations with more destabilising effects on the protein will impair function, and therefore fitness (Bershtein et al. 2012; Sarkisyan et al. 2016). Using an empirical forcefield implemented by FoldX (Schymkowitz et al. 2005) we estimated the change in the Gibbs free energy of folding ($\Delta\Delta$G) caused by each of the observed mutations. For each mutant we estimated the $\Delta\Delta$G based on the structure of the RpoB subunit alone and of the whole RNA polymerase (RNAP) complex. These estimates were correlated, though with outliers corresponding to one specific AA position (overall Spearman's rho = 0.558, P = 0.00291, Figure S15). In both cases the majority of mutations were predicted to be destabilising ($\Delta\Delta$G>0) (Table S2). When fitting a statistical model to predict fitness, as measured by the proxy of AUC, from $\Delta\Delta$G of the RpoB subunit and $\Delta\Delta$G of the RNAP complex we find that only the former effect is significant. We find a significant negative correlation between AUC and $\Delta\Delta$G of the RpoB subunit (F = 18.9, DF = 1, 318, $P = 1.84 \times 10^{-5}$, Statistical Model 7, Figure S16), consistent with the hypothesis that more destabilising mutations will impair fitness to a greater extent. However, although this correlation is significant, the proportion of observed variance in AUC explained by the $\Delta\Delta$G of the RpoB subunit is small (see Statistical Model 7). These results indicate that protein stability, while significant, is not the sole determinant of the pleiotropic growth effects of *rpoB* mutations. Therefore, although a bias towards selecting mutations with more destabilising effects on protein folding may lead to negative shifts in the DFE_{β}, much of the variance in fitness effects of mutations is not explained by protein stability.

Supplementary References

Bershtein S, Mu W, Shakhnovich EI. 2012. Soluble oligomerization provides a beneficial fitness effect on destabilizing mutations. *Proceedings of the National Academy of Sciences* 109: 4857–4862. DOI: 10.1073/pnas.1118157109.

Sarkisyan KS, Bolotin DA, Meer MV, Usmanova DR, Mishin AS, Sharonov GV, Ivankov DN, Bozhanova NG, Baranov MS, Soylemez O, et al. 2016. Local fitness landscape of the green fluorescent protein. *Nature* 533: 397–401. DOI: 10.1038/nature17995.

Schymkowitz J, Borg J, Stricher F, Nys R, Rousseau F, Serrano L. 2005. The FoldX web server: an online force field. *Nucleic Acids Research* 33: W382–W388. DOI: 10.1093/nar/gki387.

Graves S, Piepho H-P, Dorai-Raj LS with help from S. 2024. multcompView: Visualizations of paired comparisons. DOI: 10.32614/CRAN.package.multcompView.

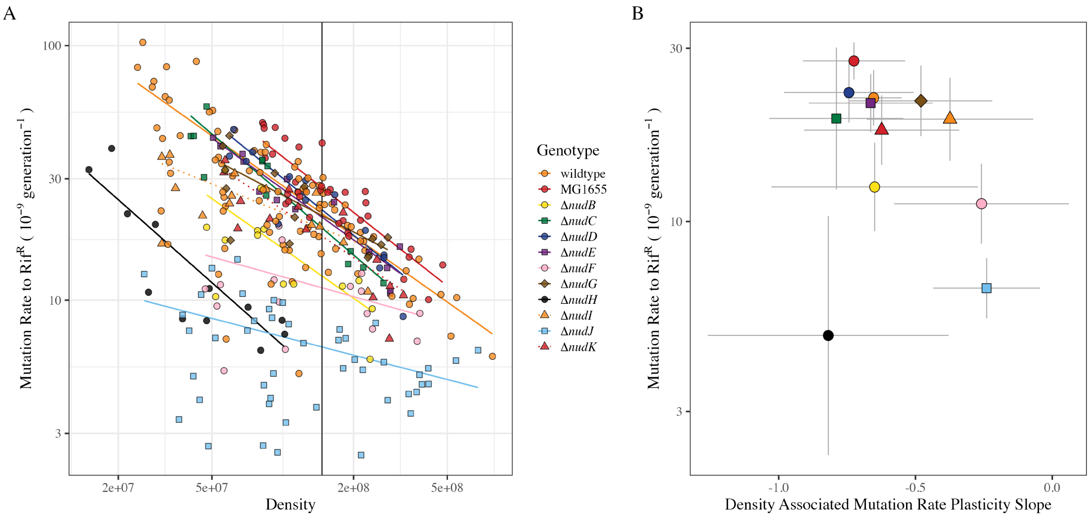

Figure S1: **Data underlying mutation rate estimates for Nudix hydrolase knockouts.** A) Mutation rate to rifampicin resistance (mutational events per division (x10^{9})) plotted as a function of final population density (CFU per mL). Raw data points are normalised by subtracting random effects associated with experimental block (see supplementary Statistical Methods document). B) Coefficients with 95% CI from a regression model fitting mutation rate as a function of genotype and population density with interaction (Methods). Wildtype BW25113 is shown as orange circles ($N_{\text{Fluctuation Assays}} = 92$, $N_{\text{Parallel Cultures}} = 1613$), wildtype MG1655 is shown as red circles ($N_{\text{FA}} = 35$, $N_{\text{PC}} = 567$), **Δ*nudB*** is shown as yellow circles ($N_{\text{FA}} = 14$, $N_{\text{PC}} = 228$), **Δ*nudC*** is shown as green squares ($N_{\text{FA}} = 12$, $N_{\text{PC}} = 193$), **Δ*nudD*** is shown as dark blue circles ($N_{\text{FA}} = 14$, $N_{\text{PC}} = 227$), **Δ*nudE*** is shown as purple squares ($N_{\text{FA}} = 14$, $N_{\text{PC}} = 228$), **Δ*nudF*** is shown as pink circles ($N_{\text{FA}} = 16$, $N_{\text{PC}} = 259$), **Δ*nudG*** is shown as brown diamonds ($N_{\text{FA}} = 14$, $N_{\text{PC}} = 226$), **Δ*nudH*** is shown as black circles ($N_{\text{FA}} = 12$, $N_{\text{PC}} = 195$), **Δ*nudI*** is shown as orange triangles ($N_{\text{FA}} = 14$, $N_{\text{PC}} = 225$), **Δ*nudJ*** is shown as light blue squares ($N_{\text{FA}} = 47$, $N_{\text{PC}} = 1089$), **Δ*nudK*** is shown as red triangles ($N_{\text{FA}} = 14$, $N_{\text{PC}} = 226$). Raw data can be found in data file S3.

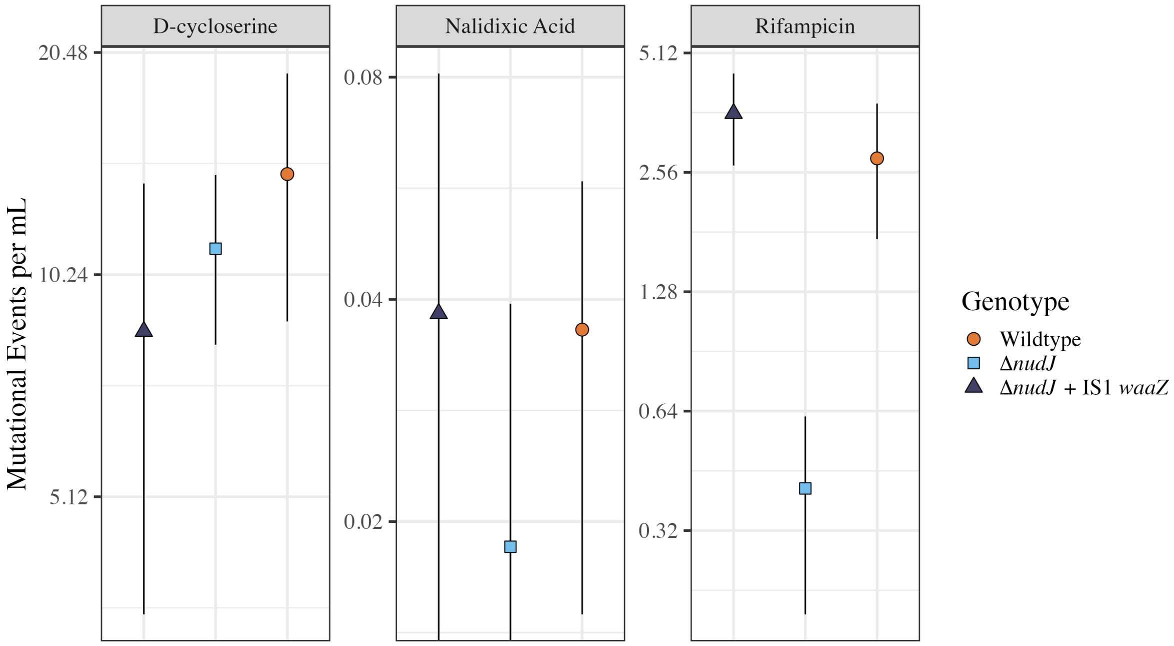

Figure S2: **Qualitative antimutator effect of Δ*nudJ* is consistent across multiple marker loci.** Fluctuation assays to D-cycloserine (5 mg per L), nalidixic acid (30 mg per L) and rifampicin (50mg per L) resistance were carried out using parallel cultures of 400µL, 1.75mL and 1mL respectively. The number of parallel cultures for each fluctuation assay (left to right) are as follows: 22, 44, 22, 47, 94, 179, 22, 44, 22 . Raw data can be found in data file S14.

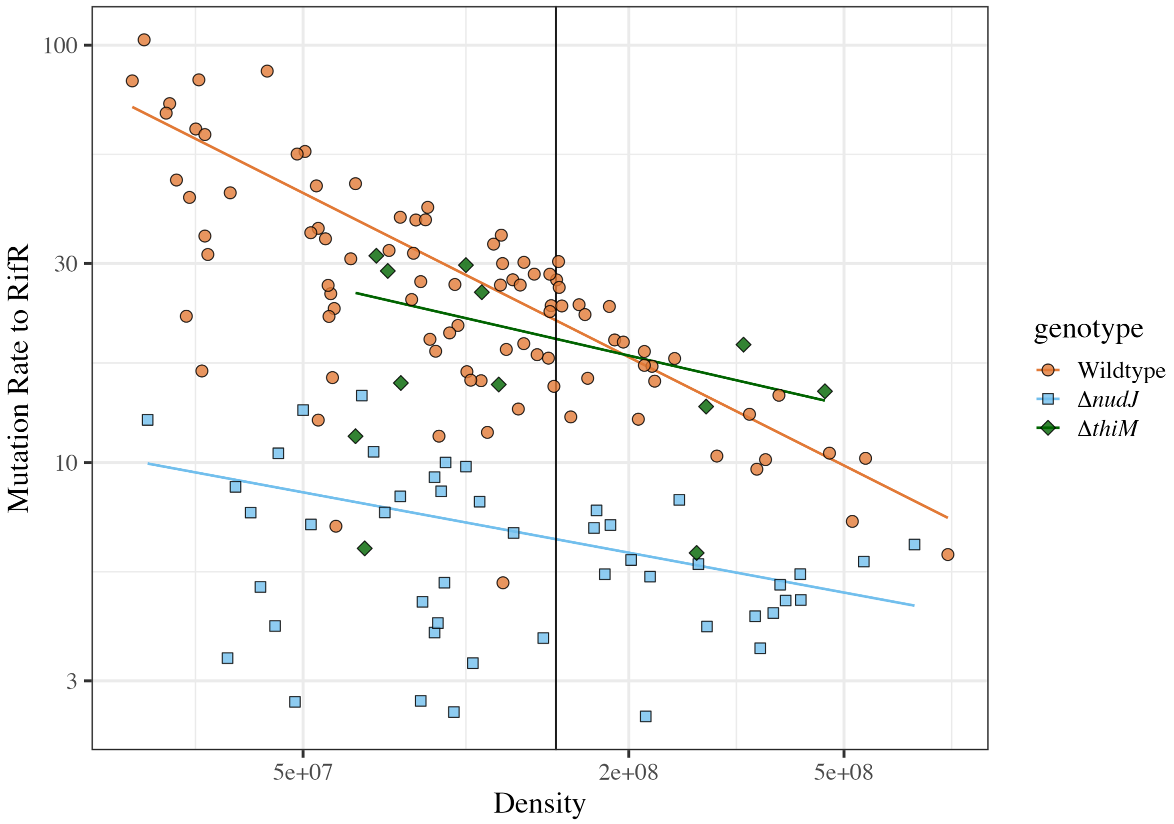

Figure S3: **Mutation rate of *thiM* deletant.** Mutation rate to rifampicin resistance (mutational events per division (x10^9)) plotted as a function of final population density (CFU per mL). Wildtype is shown as orange circles ($N_{\text{Fluctuation Assays}} = 92$, $N_{\text{Parallel Cultures}} =$ 1613, **Δ***nudJ* as blue squares ($N_{\text{FA}} = 47$, $N_{\text{PC}} = 1089$ and **Δ***thiM* as green diamonds ($N_{\text{FA}} =$ 12, $N_{\text{PC}} = 204$). Raw data points are normalised by subtracting random effects associated with experimental block (see supplementary Statistical Methods document). Lines of best fit are shown with the relevant colour for each genotype as fitted by Statistical model 1. Raw data can be found in data file S3.

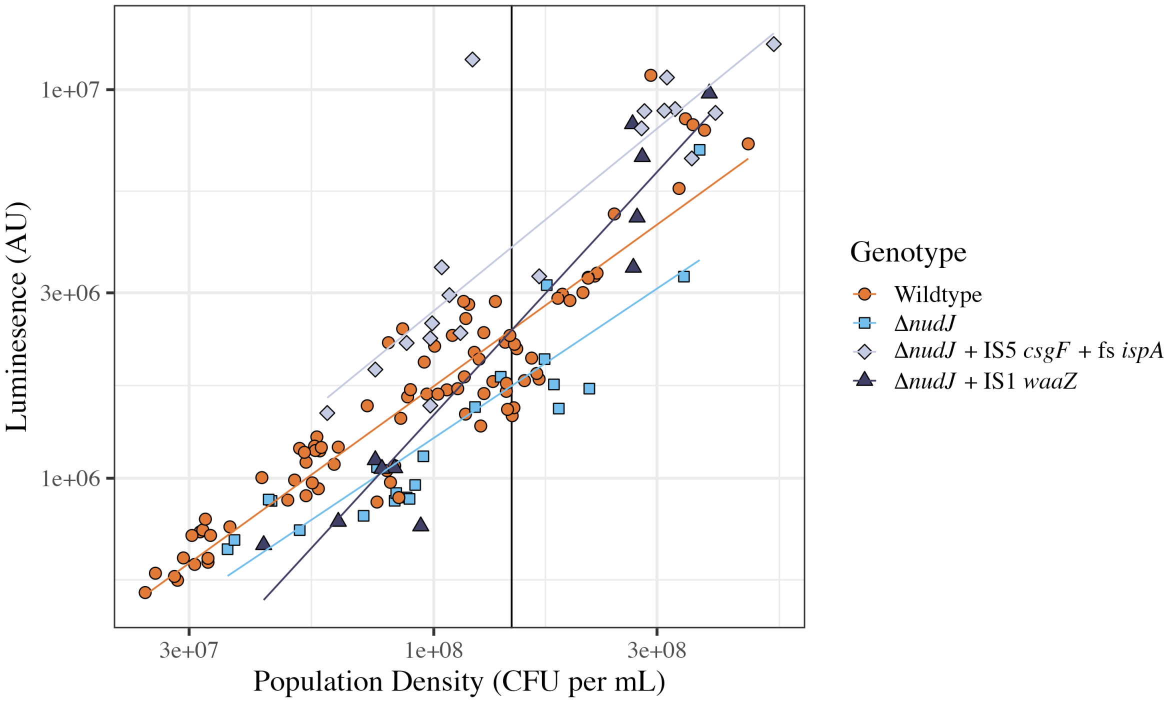

Figure S4: **ATP based luminescence assay.** Luminescence in arbitrary units (AU) is plotted as a function of population density. All luminescence measurements are normalised by subtracting random effects of experimental block and plate as well as fixed effects of experimenter identity, as estimated by Statistical Model 2. Lines of best fit are shown as estimated by Statistical Model 2. The wildtype is shown as orange circles ($N = 88$), **Δ***nudJ* is shown as pale blue squares ($N = 22$), **Δ***nudJ* + IS5 *csgF* + fs *ispA* is shown as grey diamonds ($N = 19$) and **Δ***nudJ* + IS1 *waaZ* is shown as dark blue triangles ($N = 11$). Note log-log axes. Raw data can be found in data file S3.

A

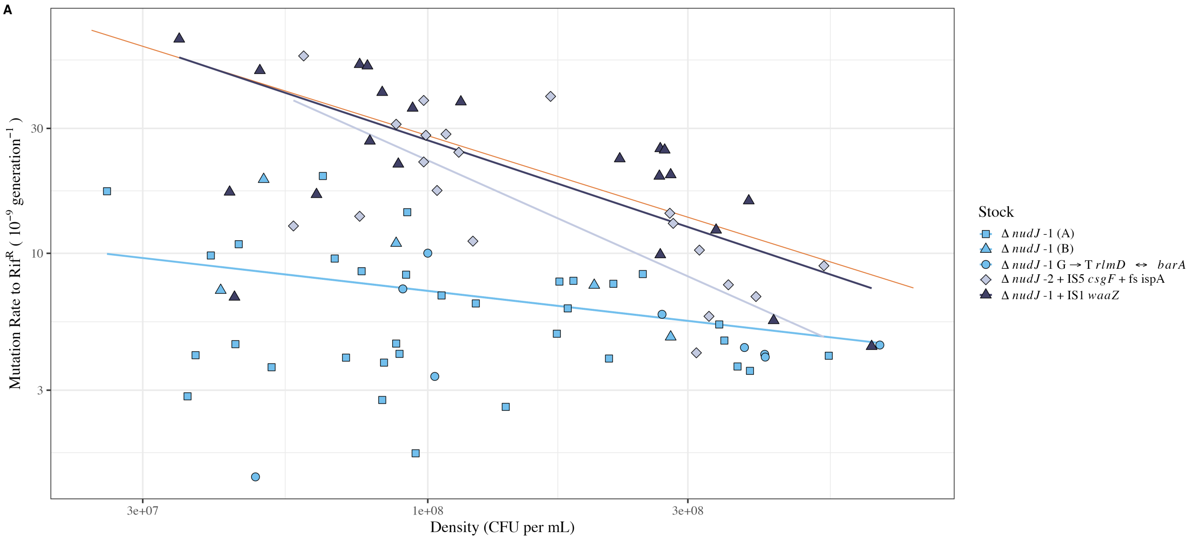

B

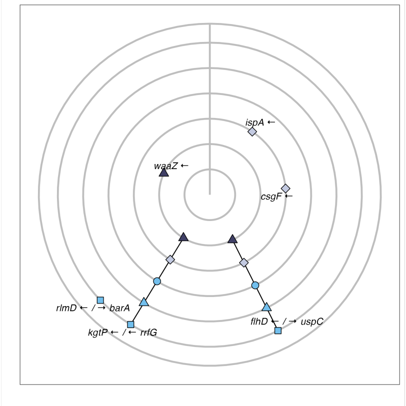

C

Strain	position	mutation	annotation	gene
Δ *nudJ* -2 + IS5 *csgF* + fs ispA	436547	+CG	coding (11/900 nt)	ispA ←
Δ *nudJ* -2 + IS5 *csgF* + fs ispA	1097379	IS5 (–) +4 bp	coding (202-205/417 nt)	csgF ←
Δ *nudJ* -1 G → T *rlmD* ← / → *barA*	2908408	G→T	intergenic (-49/-8)	rlmD ← / → barA
Δ *nudJ* -1 + IS1 *waaZ*	3793366	IS1 (–) +9 bp	coding (183-191/852 nt)	waaZ ←
All	1972967	IS5 (+) +4 bp	intergenic (-513/-264)	flhD ← / → uspC
All	2719426	T→C	intergenic (-321/+2)	kgtP ← / ← rrfG

Figure S5: **Mutation rates of 5 Δ*nudJ* freezer stocks.** **Δ***nudJ*-1 and **Δ***nudJ*-2 denote the 2 independently created *nudJ* deletant strains in the Keio collection. **Δ***nudJ*-1 (A) & (B) indicate the 2 separate freezer stocks taken from Keio deletant **Δ***nudJ*-1 with no secondary mutations. All other stocks are identified by their secondary mutations. A) Mutation rate to rifampicin resistance (mutational events per division (x10^9)) plotted as a function of final population density (CFU per mL). The orange line shows fitted values for the wildtype as a reference. 2 stocks show no off target mutations and are shown as light blue squares (**Δ***nudJ*-1 (A), $N_{\text{Fluctuation Assays}} = 19$, $N_{\text{Parallel Cultures}} = 306$) and light blue triangles (**Δ***nudJ*-1 (B), $N_{\text{FA}} = 0$, $N_{\text{PC}} = 0$), one stock had no off target genic mutations and an intergenic G>T transversion between *rlmD* and *barA* is shown as light blue circles (**Δ***nudJ*-1 + G⟶T *rlmD* ⟵/⟶ *barA*, $N_{\text{FA}} = 0$, $N_{\text{PC}} = 0$), the two stocks with off target genic mutations are shown as grey diamonds (**Δ***nudJ*-2 + *ispA* fs + *csgF* IS5, $N_{\text{FA}} = 0$, $N_{\text{PC}} = 0$) and dark blue triangles (**Δ***nudJ*-1 + *waaZ* IS1, $N_{\text{FA}} = 0$, $N_{\text{PC}} = 0$). Levels are combined for the 3 stocks with no off target genic mutations, improving the fit of the model (Statistical Model 1). Note log-log axis scales in panel A. B) Visualisation of identified mutations in 5 **Δ***nudJ* stocks. Vertical line represents the origin of replication. C) Table of secondary mutations identified in 5 **Δ***nudJ* stocks. Mutations are identified with reference to the wildtype BW25113 and positions are given for the reference genome. All predictions made with breseq (Deatherage and Barrick 2014). Raw data can be found in data file S3 and S11.

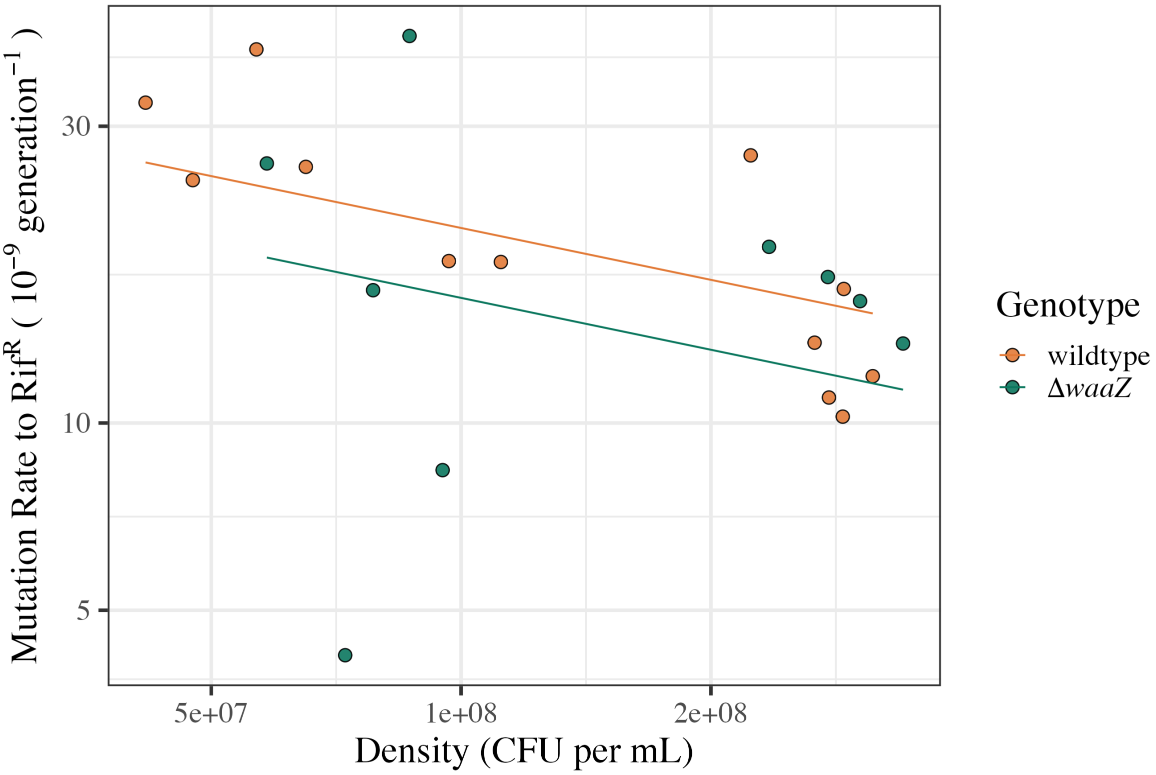

Figure S6: **Mutation rate of Δ*waaZ* is not elevated compared to wildtype BW25113** Mutation rate to rifampicin resistance (mutational events per division (x10^9)) plotted as a function of final population density (CFU per mL). Genotype is indicated by colour: orange circles = wildtype, *N*=12; green circles = **Δ***waaZ*, *N*=9. Mutation rate is estimated using a liquid based fluctuation assay to rifampicin resistance (Methods). Note log-log axes. Raw data can be found in data file S4.

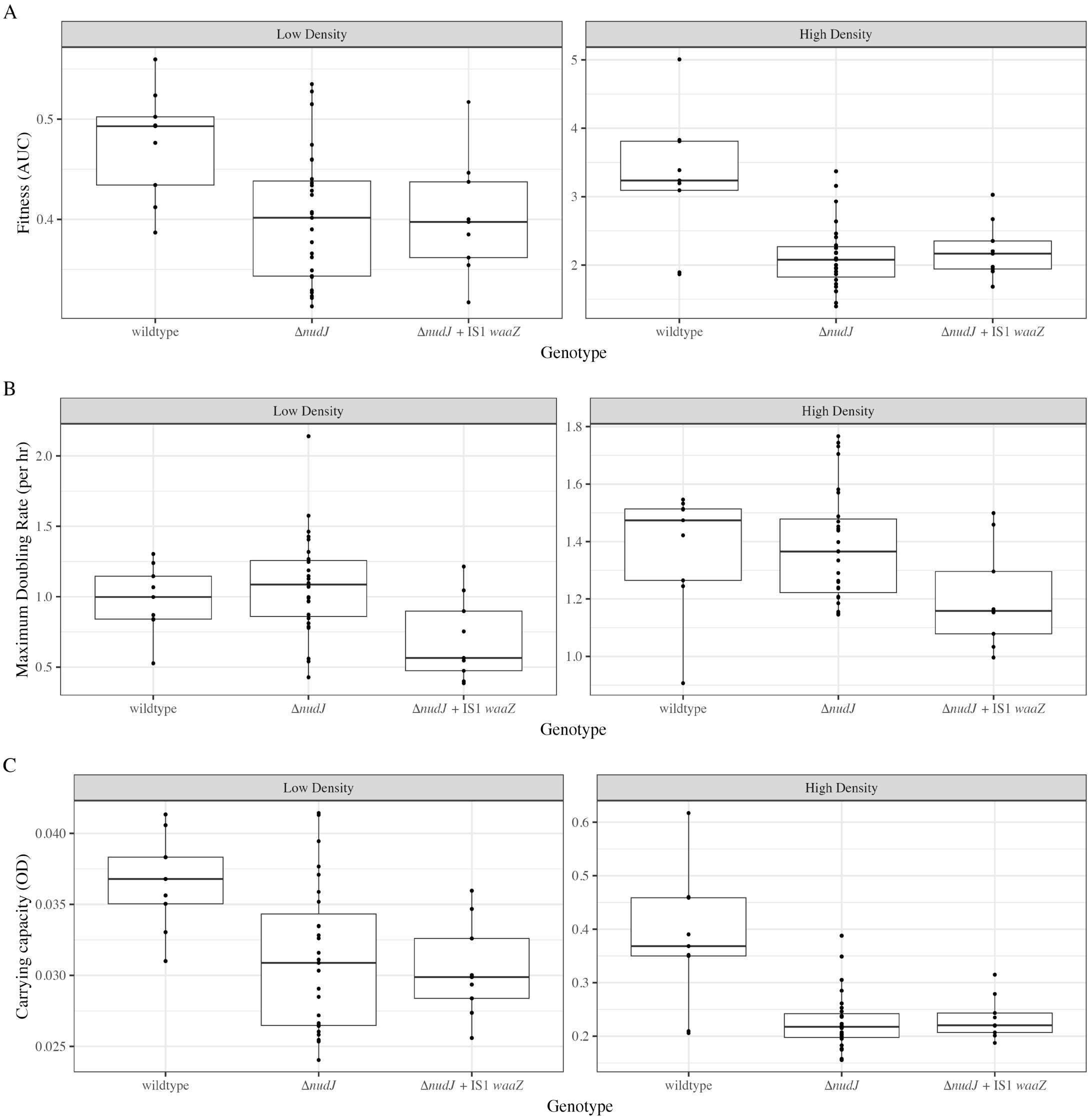

Figure S7: **Loss of fitness in Δ*nudJ* strains results from lower carrying capacity** Data from growth curves with 3 biological replicates each with 3 technical replicates for each genotype aside from **Δ***nudJ* for which 9 biological replicates each with 3 technical replicates were collected. Raw data can be found in data file S12.

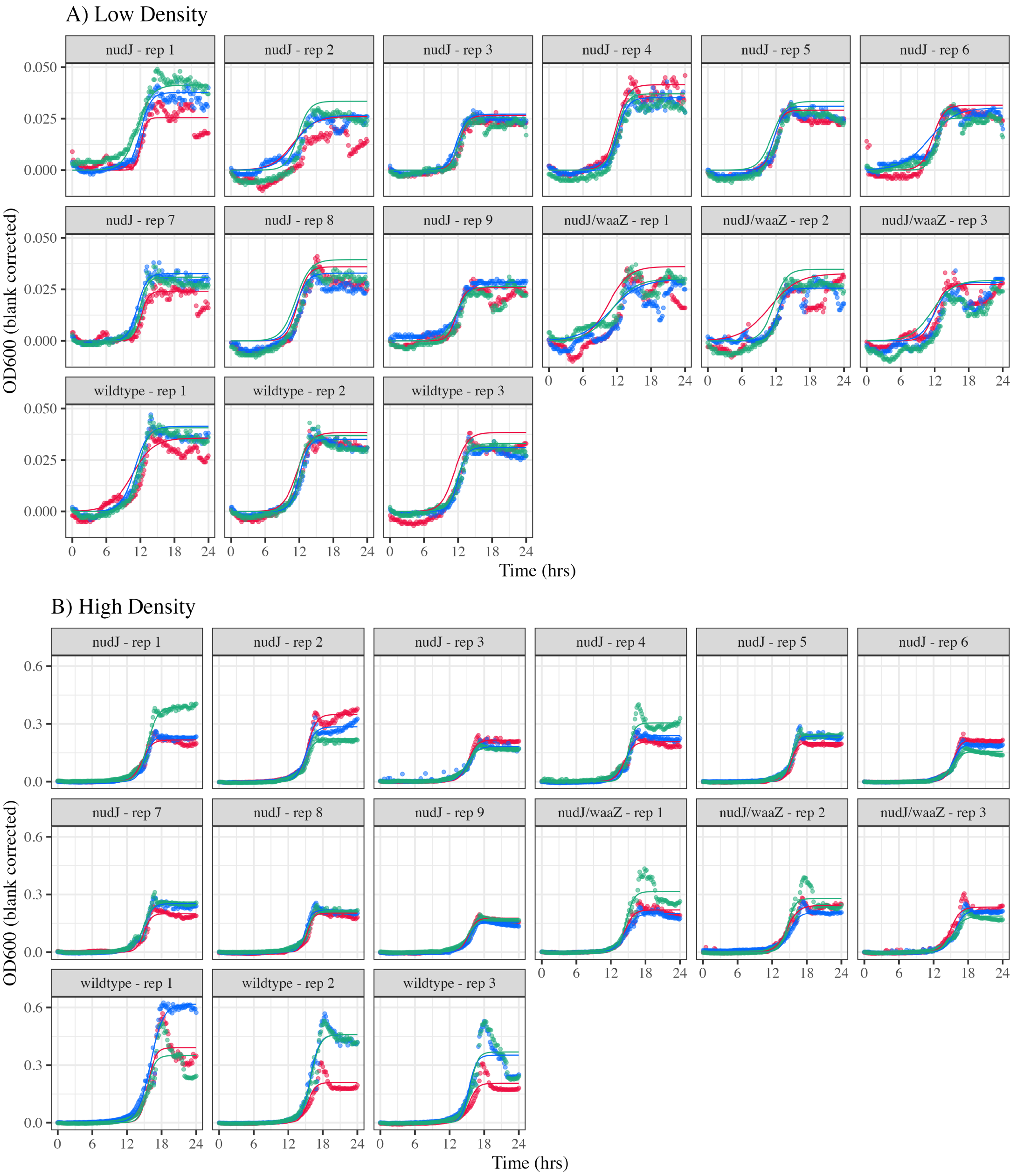

Figure S8: **Raw data from growth curves with wildtype BW25113 and Δ*nudJ* strains** The top plots show low density (80mg.L^{-1} glucose) and the bottom plots show high density (1000mg.L^{-1} glucose), note different y axis limits for these 2 sets of plots. Each facet includes a separate biological replicate with 3 technical replicates coloured in blue, green and red. Points show raw data and lines show associated logistic fits estimated by R package growthcurver (Sprouffske 2020). Raw data can be found in data file S12.

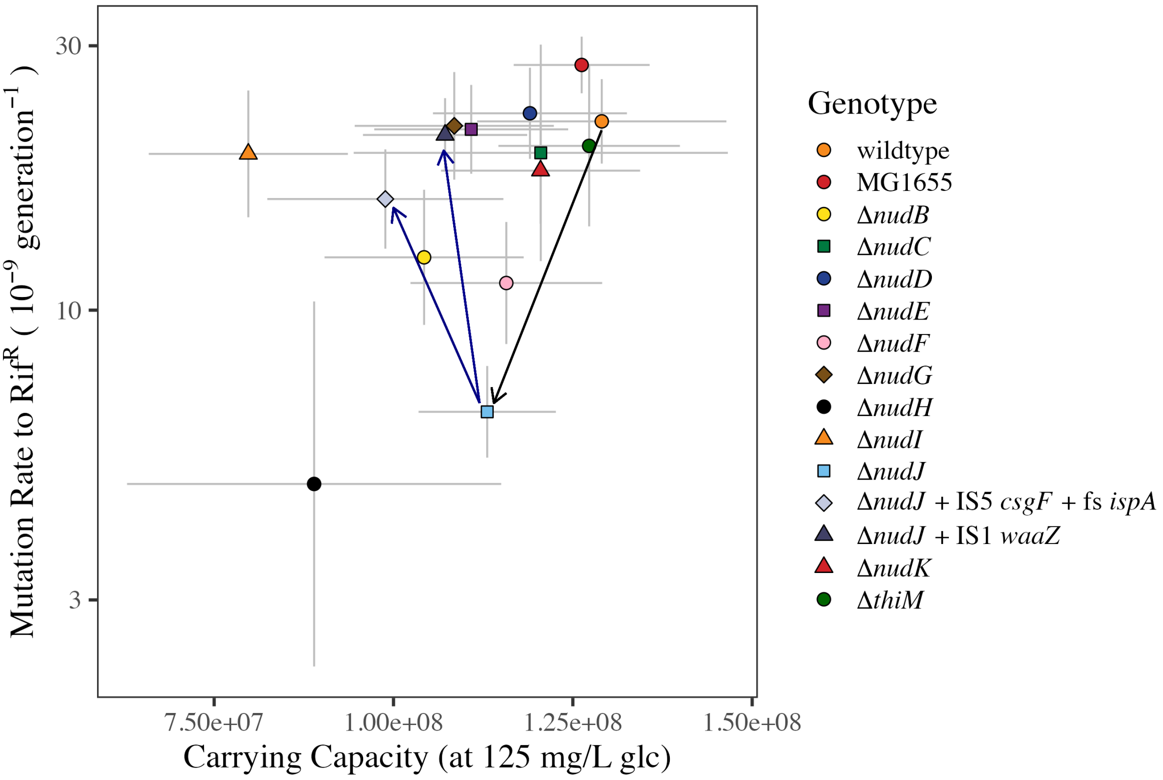

Figure S9: **Mutation rate against carrying capacity: any growth defect in Δ*nudJ* is not recovered by secondary mutations in *waaZ*/*csgF*+*ispA*.** Carrying capacity at 125mg per L glucose is estimated from final CFU counts on non-selective agar from 24 hour growth. Black line shows the reduction in carrying capacity and mutation rate caused by *nudJ* deletion. Blue arrows show that mutation rate, but not carrying capacity, reverts to the wildtype phenotype after secondary mutations. Carrying capacity at 125mg/L glucose fitted by Statistical Model 8 and mutation rate at mean density fitted by Statistical Model 1. Error bars show 95% CI for these parameter estimates. Raw data can be found in data file S3.

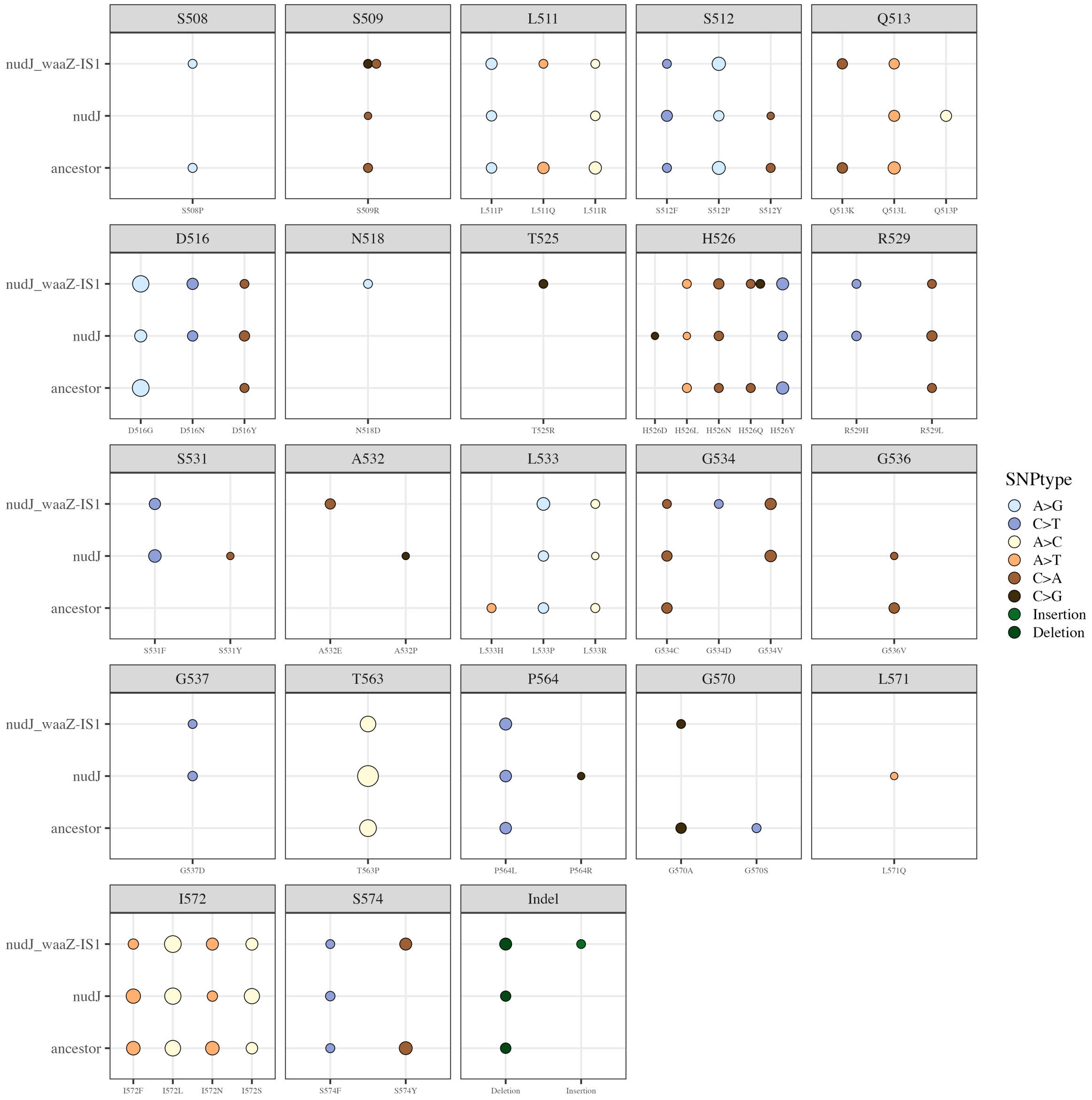

Figure S10: **All observed AA changes in Sanger sequencing of the rifR determining region.** Each subplot indicates a targetted amino acid with the observed replacement amino acid given on the x axis, and the strain in which the given AA substitution was observed given on the y axis. Points are coloured by mutational class and size indicates the proportion of mutations in the given strain accounted for by the given AA substitution (range 0.5% - 24%). Raw data can be found in data file S2.

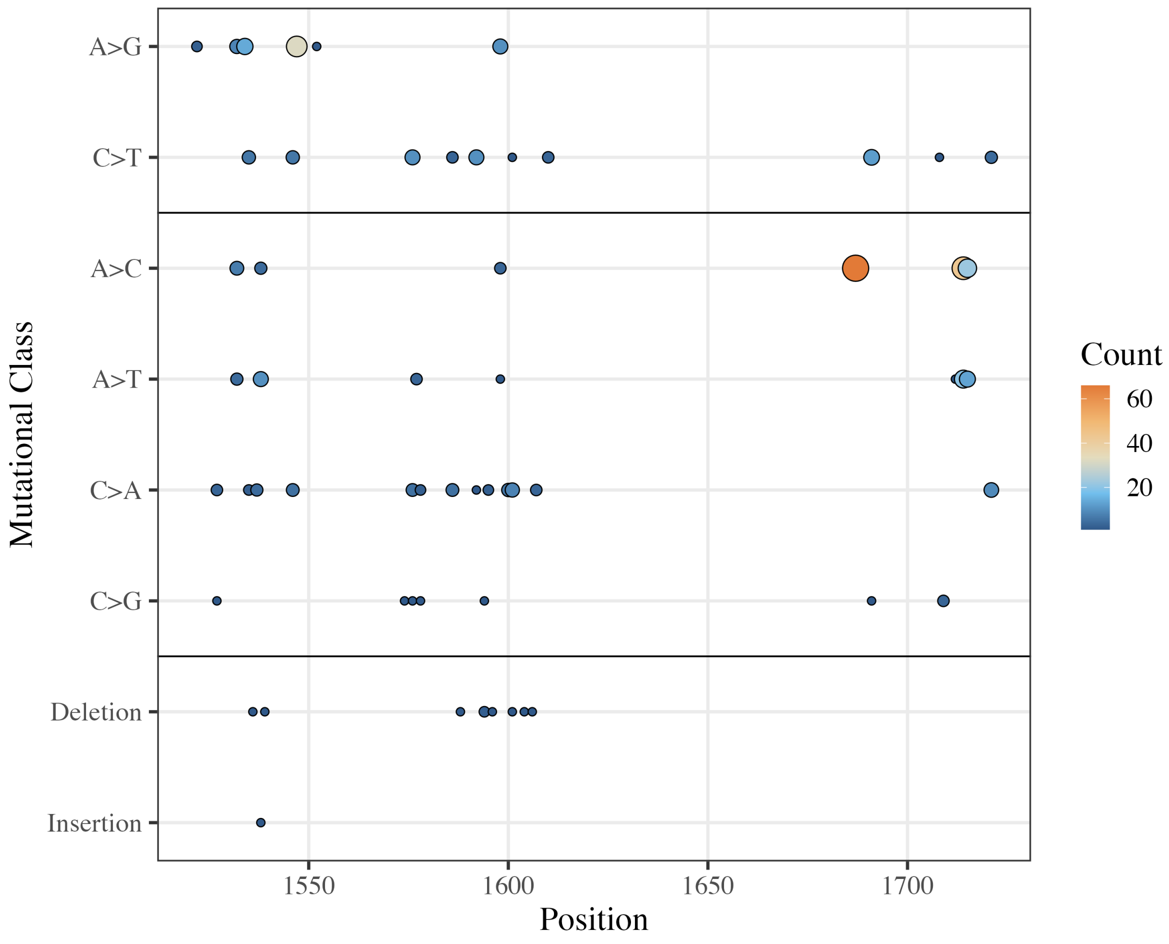

Figure S11: **All observed mutations observed in the wildtype BW25113, Δ*nudJ* and Δ*nudJ* + IS1 *waaZ* backgrounds combined.** Mutations are plotted by position in the genome along the x axis and divided into the 8 observed mutational classes along the y axis. The three sections from top to bottom are transitions, transversions & indels. Each point represents a unique mutation with size and colour indicating the number of times the given mutation was observed among a total of 394 mutations (range 1 - 66). Raw data can be found in data file S2.

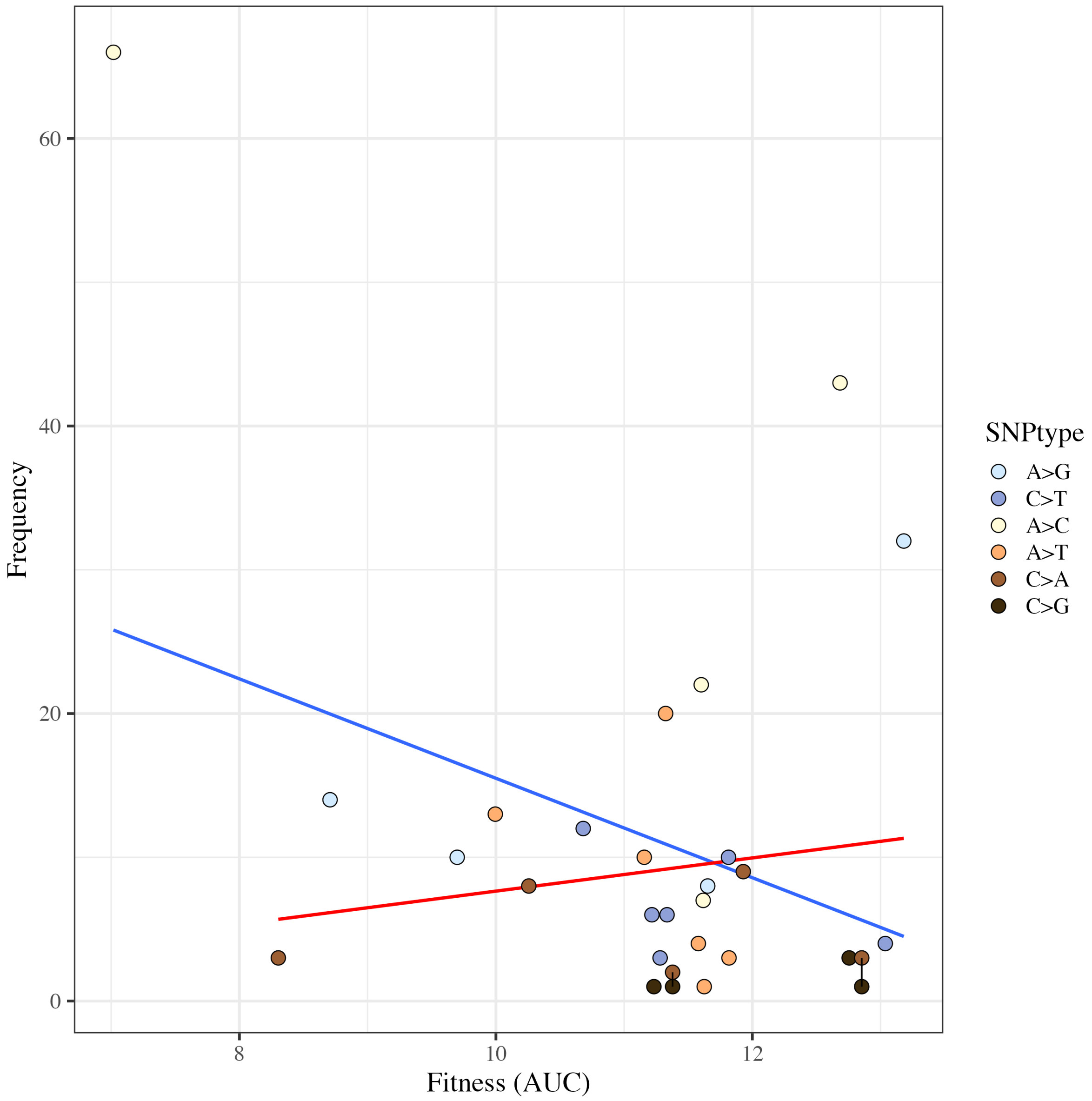

Figure S12: **Frequency of *rpoB* mutant observations across all genetic backgrounds against fitness.** Points show 29 unique mutations resulting in 27 unique AA substitutions, where 2 mutations can cause the same AA sub the points are joined with a black line. There is no significant linear relationship between fitness, as measured in minimal M9 media at 37 degrees without rifampicin, and frequency of mutant observation either including the whole data set (F = 3.64, *P* = 0.0671 Statistical Model 9) or excluding the A>C outlier with >60 observations (AA substitution T563P) (F = 0.521, *P* = 0.477, Statistical Model 10). Raw data can be found in data file S2 and S5.

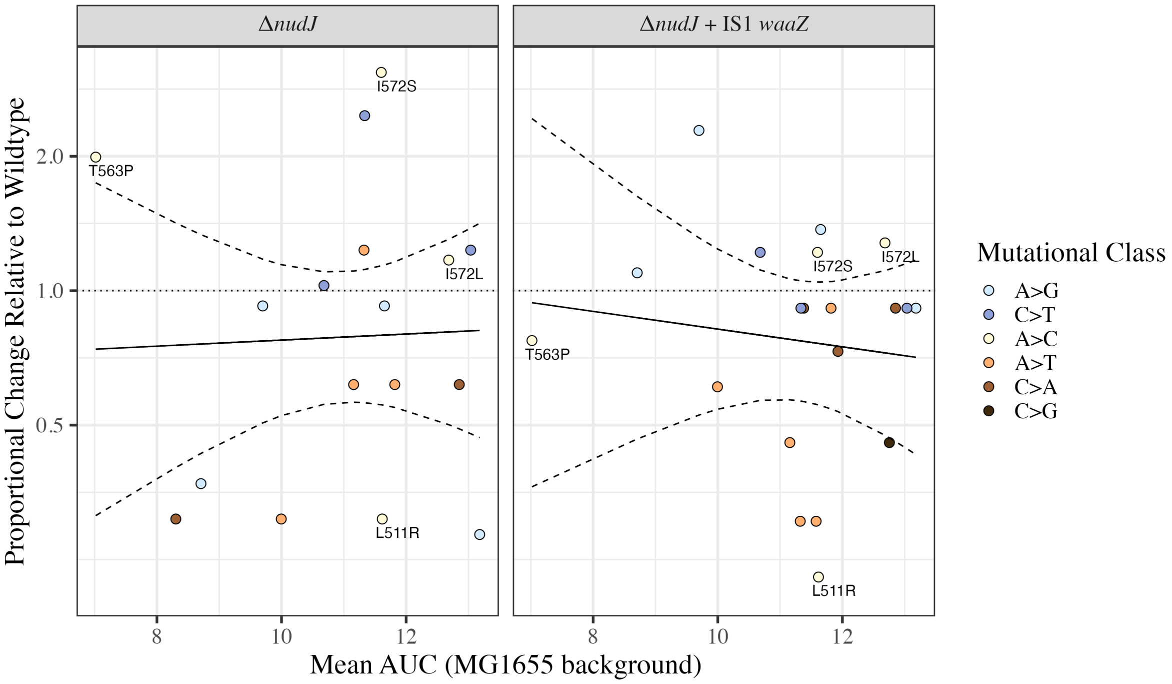

Figure S13: **Test for enrichment of low-fitness rifR mutants in Δ*nudJ* strains.** Each point represents an individual rifR mutant for which fitness by the proxy of AUC has been measured in the MG1655 wildtype background in M9 minimal media in the absence of rifampicin (shown on the x-axis). The relative change in each mutant's representation in **Δ***nudJ* strains compared to the wildtype BW25113 is shown on the y-axis. For example while T563P makes up 12% of sequenced mutants in the wt background it makes up 24% and 9% of mutants in the **Δ***nudJ* and **Δ***nudJ* + IS1 *waaZ* backgrounds, giving enrichment values of 2.0 and 0.77 respectively. All fitness measurements used here are measured in M9 minimal media in the absence of rifampicin at 37°C; the test environment closest to that of our fluctuation assays. Fitted lines show the a linear model fitting log2 transformed proportional change as a function of AUC and **Δ***nudJ* strain identity including an interaction. No significant effect of AUC ($F_{DF=1}$ = 0.0361, P = 0.851) strain identity ($F_{DF=1}$ = 0.0064, P = 0.937) or their interaction ($F_{DF=1}$ = 0.186, P = 0.669) were observed. All A>C mutant are labelled with their amino acid substitution. Note log scale y-axes as proportions are shown. Panels are divided by the **Δ***nudJ* background in which proportional change is shown. Raw data can be found in data file S2 and S5.

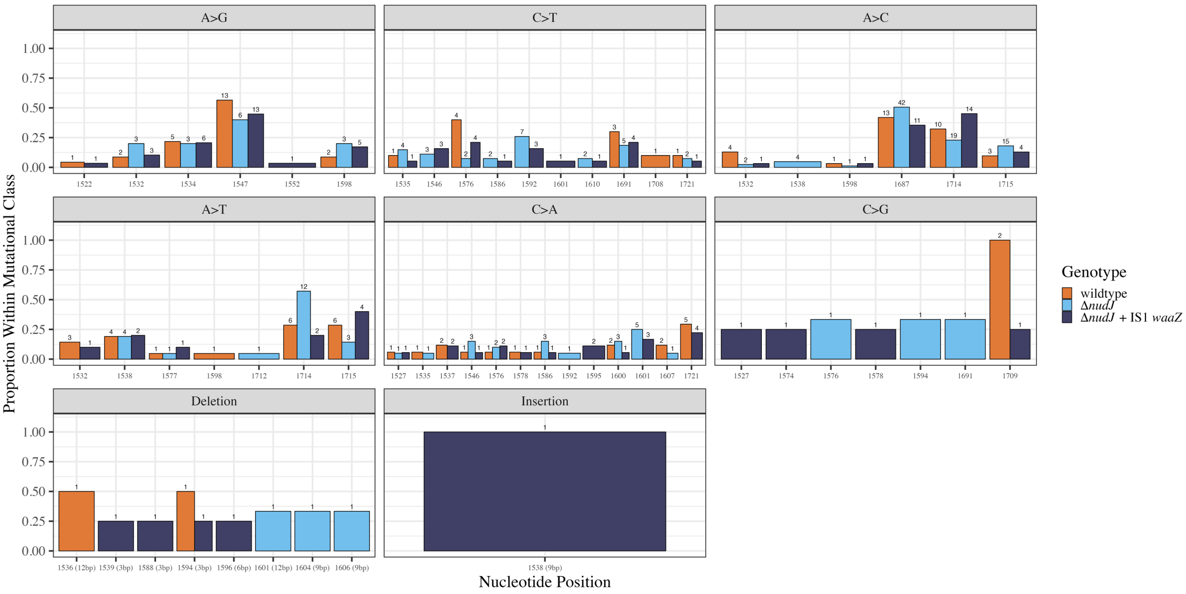

Figure S14: **Proportion of mutants accounted for by each unique mutation within each mutational class.** Bar colour indicates the genetic background in which the mutant evolved. Numbers above the bars indicate the number of sequenced mutants accounted for by the given mutation-background combination. Raw data can be found in data file S2.

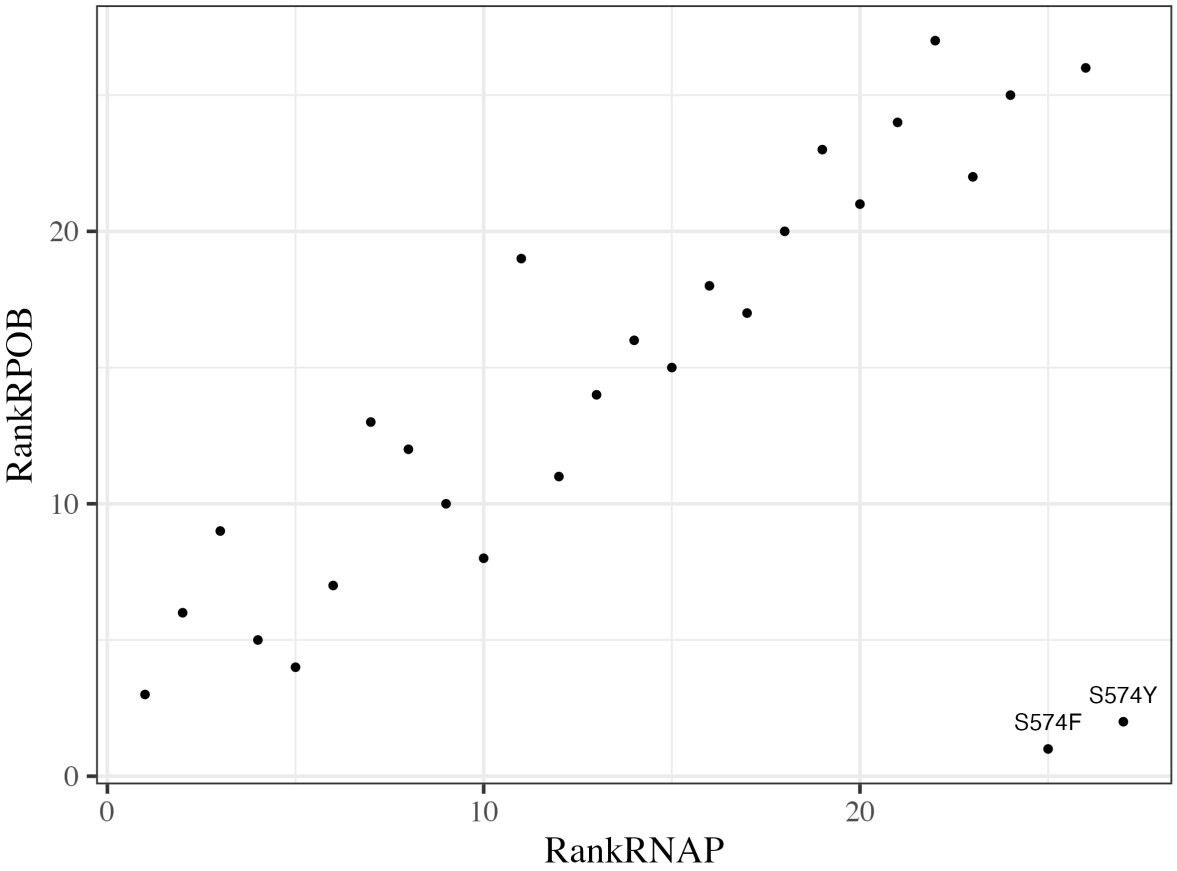

Figure S15: **Ranks of the (de)stabilising effects of *rpoB* mutants on RpoB and the RNAP complex are highly correlated.** Outliers at the 574th AA residue are labelled. Raw data can be found in Table S2. Higher ranks indicate higher values of **ΔΔ**G therefore while S574F are predicted to be highly destabilising to the RNAP complex they are the least destabilising to the RpoB subunit. Raw data can be found in data file S6.

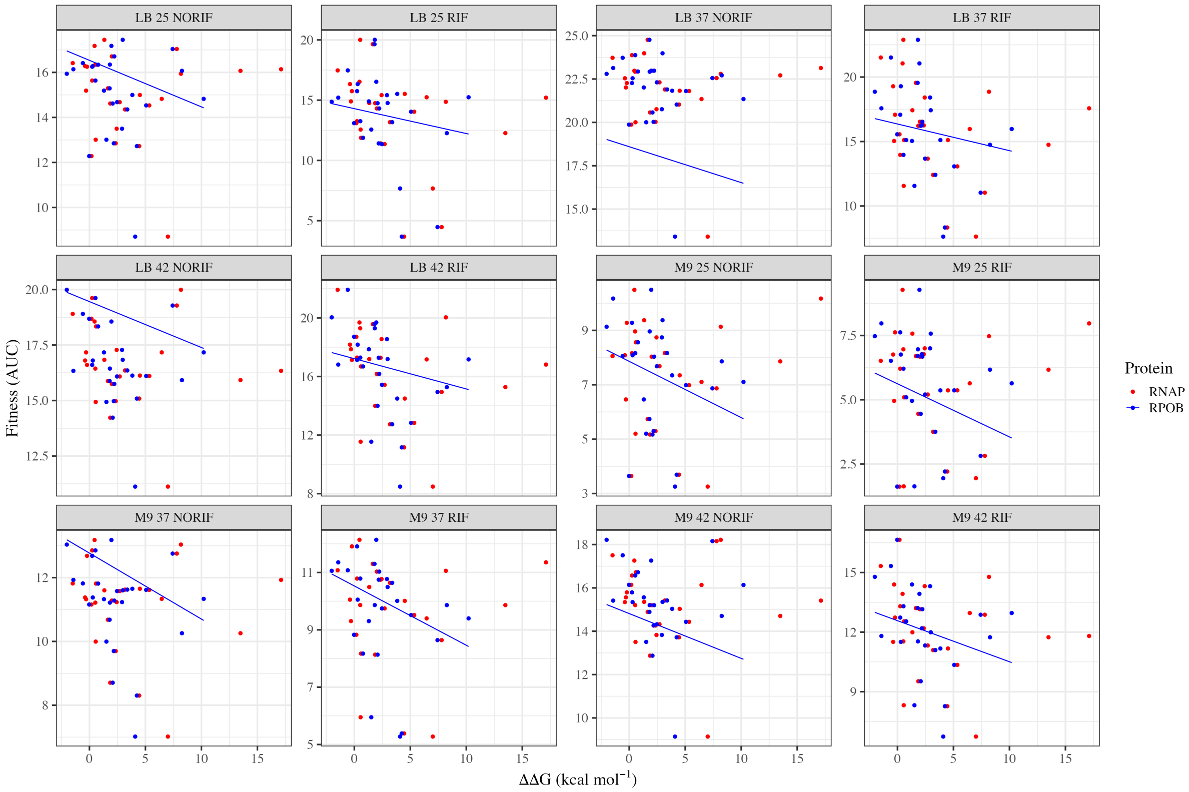

Figure S16: **Decreased stability of protein folding results in decreases in mutant fitness across 12 environments**. Fitness (measured by the proxy of AUC from 24 hour growth curves) is plotted as a function of the change in protein stability to RNAP (red) and RpoB (blue) caused by the given mutation. Greater values of **ΔΔ**G indicate reduced stability. Blue lines show predictions from Statistical Model 7. Note varying y-axis limits. Raw data can be found in data file S5 and S6.

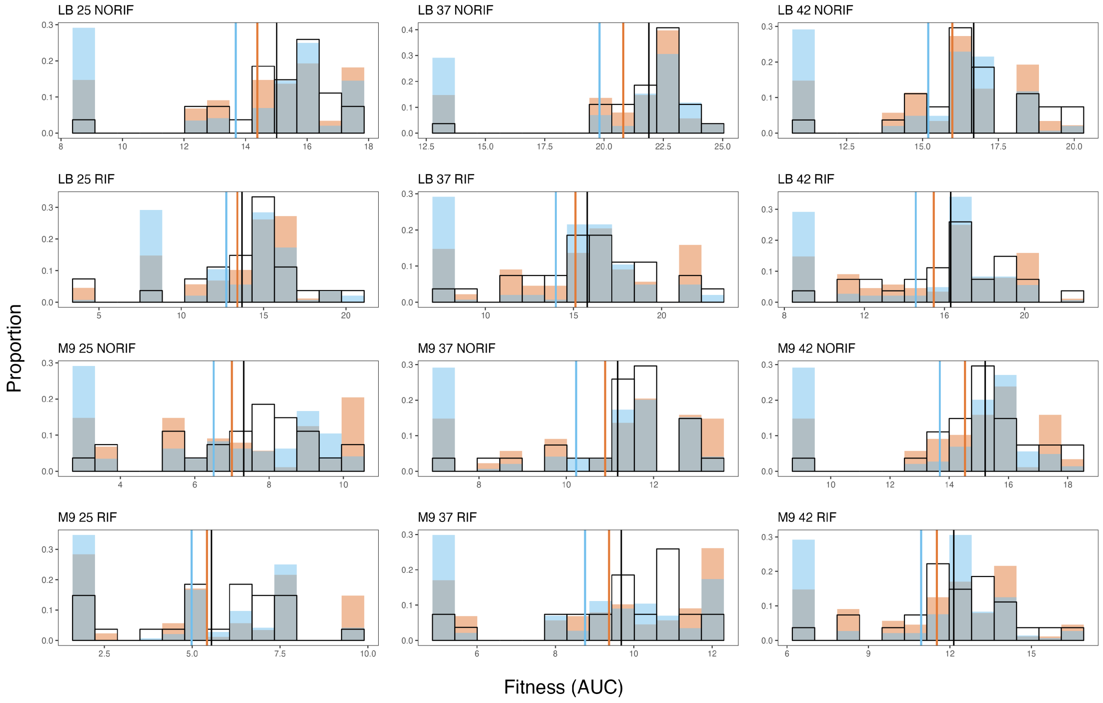

Figure S17: **DFE of rifR mutants differs by background genotype** AUC of 24 hour growth curves of rifR mutants in the MG1655 wildtype background across 12 different environments defined by media type, temperature and presence of rifampicin. The black outline shows the null DFE, with the DFE_{β} of **Δ***nudJ* shown in blue and of the wildtype shown in orange and the means of these groups shown by vertical lines in the appropriate colour. 27 of 56 unique resistance mutations identified in this study are included. Raw data can be found in data file S2 and S5.

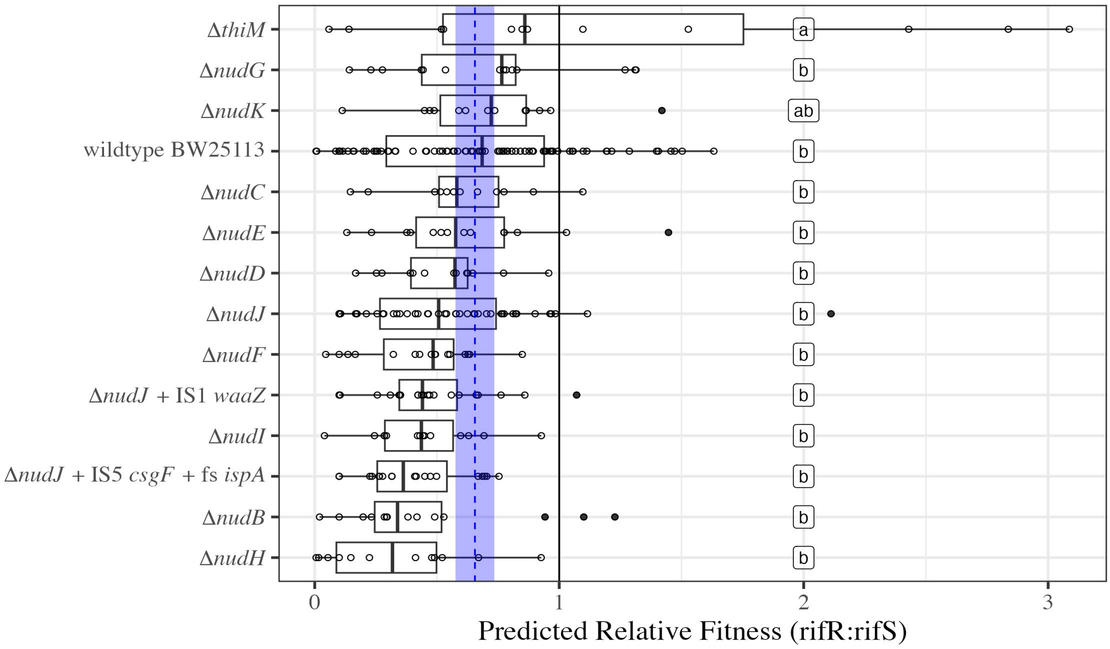

Figure S18: **Predicted cost of rifR mutants relative to rifS cells in each genetic background.** Predictions coestimated with mutational events by flan package (Mazoyer et al. 2021). For each genotype boxplots show the predictive fitness of rifR mutants relative to the rifS population from which they evolved. The middle bar of each box shows the median, for all strains this is bellow 1 indicating that, on average, rifampicin resistance incurs a cost. All invididual data points are shown as circular points, filled points indicate outliers further than 1.5 X Interquartile-range from the closest of $quartile_{0.25}$ or $quartile_{0.75}$ (left and righthand ends of the box of box). Black vertical line shows a fitness ration of 1 i.e. the rifR and rifS populations have equal fitness. Blue vertical line and shaded area show the mean and 95% CI of the rifR:rifS fitness ratio for the wildtype BW25113 strain. Letters shown on the righthand side of the plot show groups without significant difference in mean as calculated by Tukey's 'Honest Significant Difference' method (Graves et al. 2024; 2025) based on a linear model fitting relative rifR:rifS fitness as a function of genotype. Raw data can be found in data file S3.

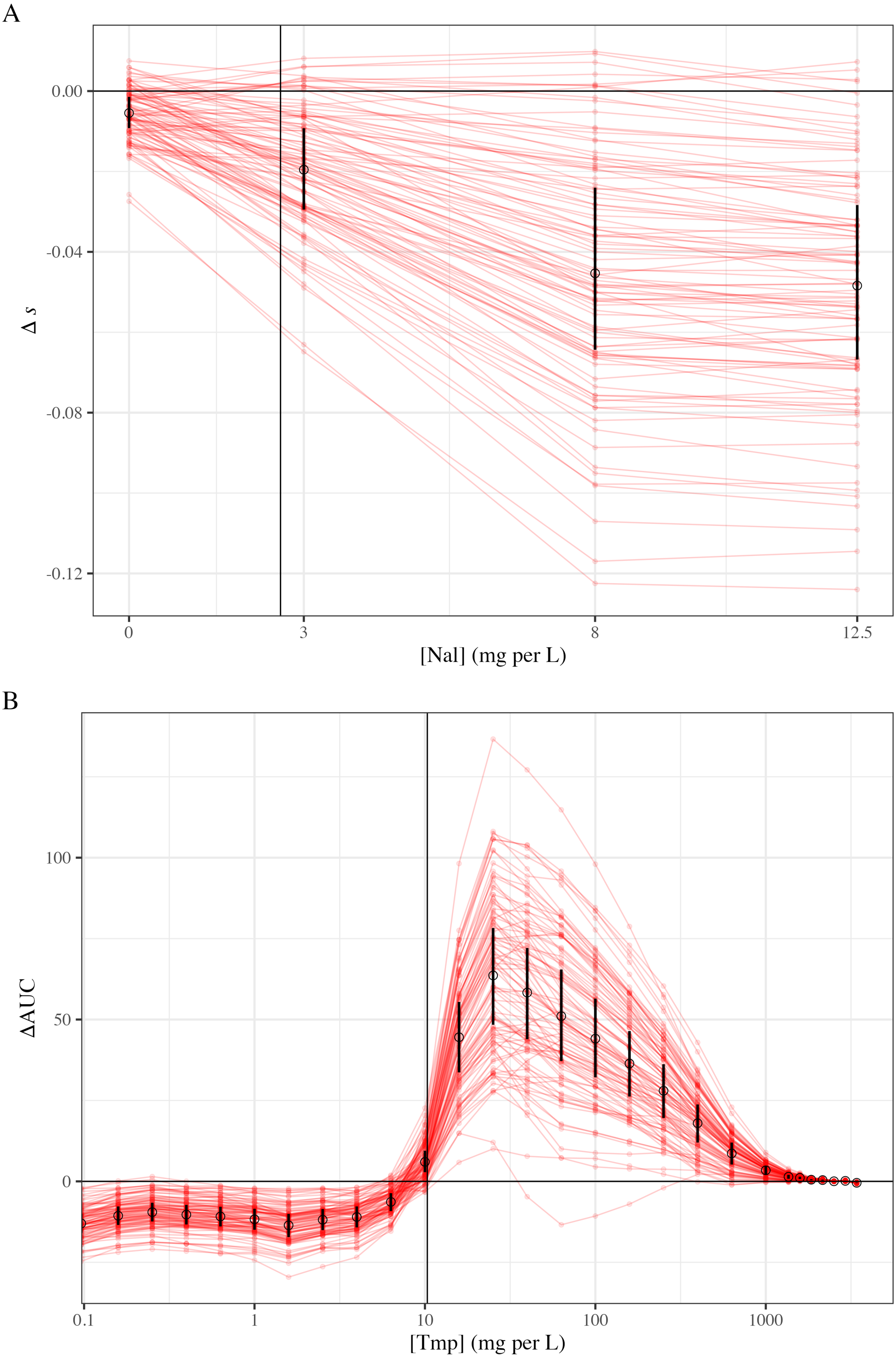
A
0.00
-0.04
-0.08
-0.12
Δ s
0
3
8
12.5
[Nal] (mg per L)
B
100
50
0
ΔAUC
0.1
1
10
100
1000
[Tmp] (mg per L)

Figure S19: **Effect of the altered mutational spectrum of Δ*nudJ* on fitness costs incurred during antibiotic adaptation.** For further details see Methods section 'Bootstrapping fitness differences …'. A) Red points connected by red lines represent the difference in mean selection coefficient (*s*) of nalidixic acid (Nal) resistant mutants accessed by **Δ***nudJ* and the wildtype (**Δ***s*) for a single bootstrap replicate. A random sample of 100 out of 1000 bootstrap replicates are shown. The Nal concentration at which *s* was measured by Harmand et al. (2017) is given on the x-axis. Black circles show the mean value of **Δ***s* across all 1000 bootstrap replicates, with the IQR across all 1000 replicates shown by black error bars. The horizontal black line shows a **Δ***s* value of of 0; points below this line indicate that mutants expected to be accessed by **Δ***nudJ* are less fit than those accessed by the wildtype and vice versa. The vertical black line represents the minimum inhibitory concentration (MIC) of Nal as given by Harmand et al. (2017) . B) As in the first panel with the following differences. The difference in average growth between mutants accessed by **Δ***nudJ* and the wildtype is given on the y-axis (**Δ**AUC). The vertical black line represents the Tmp concentration at which growth of the sensitive wildtype is suppressed to 25% of its AUC in drug-free media (IC75) as given by Palmer et al. (2015) .

Table S1: Mutation rates across nud KOs with comparison to wildtype. All mutation rates are given as the probability of a mutational event conferring rifampicin resistance occuring in a cell division (x1e9). t statistics compare the mutation rate of the given strain to the BW25113 wildtype. Mutation rates are estimated at the mean density of all fluctuation assays.

Treatment	Rate	Lower	Upper	Fold Change (vs wt)	t-value	DF	P (Dunnett's test)
Ancestor (BW25113 wt)	21.90	18.40	26.10	1.000	NA	NA	NA
MG1655	27.70	24.60	31.20	1.260	3.92	283	1.74×10^{-3}
nudB	12.50	9.41	16.50	0.568	-3.96	283	1.48×10^{-3}
nudC	19.20	12.30	30.10	0.877	-0.573	283	1.00
nudD	22.70	18.80	27.40	1.030	0.349	283	1.00
nudE	21.20	17.60	25.50	0.967	-0.354	283	1.00
nudF	11.20	8.69	14.40	0.511	-5.21	283	3.81×10^{-6}
nudG	21.50	17.20	26.90	0.982	-0.164	283	1.00
nudH	4.86	2.28	10.40	0.222	-3.91	283	1.85×10^{-3}
nudI	19.10	14.70	24.90	0.874	-1.01	283	0.999
nudJ	6.55	5.42	7.93	0.299	-12.5	283	$< 1 \times 10^{-6}$
nudJ_ispA	15.90	12.90	19.50	0.724	-3.08	283	3.91×10^{-2}
nudJ_waaZ	20.70	17.80	24.10	0.945	-0.727	283	1.00
nudK	17.90	14.30	22.30	0.815	-1.83	283	0.703
thiM	19.80	14.20	27.70	0.903	-0.599	283	> 0.999

Table S2: All amino acid substitutions observed in the RRDR in this study. ‘AAsub’ lists the amino acid substitution in RpoB resulting from the mutation. ‘Position’ lists the mutated position in the gene, in the case of insertions and deletions the first base pair of the mutation is given. ‘SNPtype’ lists the mutational class of the mutation. ‘ancestor’, ‘nudJ’ and ‘nudJ_waaZ-IS1’ list the number of times this mutation was observed in the wildtype strain BW25113, *nudJ* deletant and the *nudJ* deletant with an IS1 insertion in *waaZ*. ‘ddG_RNAP’ and ‘ddG_RPOB’ list the predicted change in the Gibbs free energy of folding (kcal per mol) caused by the given mutation to the RNAP complex and the RpoB subunit respectively. Number of sequenced mutants is: wildtype N = 92, **Δ***nudJ* N = 201 and **Δ***nudJ*+IS1*waaZ* N = 102. ‘Ins (dup)’ refers to an insertion which is a duplication of the adjacent nucleotide sequence.

AAsub	Position	SNPtype	ancestor	nudJ	nudJ_waaZ-IS1	ddG_RNAP	ddG_RPOB
S508P	1522	A>G	1	0	1	1.49	2.98
S509R	1527	C>A	1	1	1	0.247	0.537
S509R	1527	C>G	0	0	1	0.247	0.537
L511P	1532	A>G	2	3	3	4.51	3.83
L511Q	1532	A>T	3	0	1	2.71	2.47
L511R	1532	A>C	4	2	1	5.35	5.07
S512P	1534	A>G	5	3	6	1.86	2.08
S512F	1535	C>T	1	4	1	6.46	10.2
S512Y	1535	C>A	1	1	0	9.18	10
Q513K	1537	C>A	2	0	2	-0.331	0.493
Q513L	1538	A>T	4	4	2	0.183	-0.0213
Q513P	1538	A>C	0	4	0	2.06	4.61
D516N	1546	C>T	0	3	3	0.527	1.83
D516Y	1546	C>A	1	3	1	-0.423	1.32
D516G	1547	A>G	13	6	13	0.458	1.96
N518D	1552	A>G	0	0	1	1.11	1.84
T525R	1574	C>G	0	0	1	2.43	2.91
H526D	1576	C>G	0	1	0	2.36	2.05
H526N	1576	C>A	1	2	2	1.18	1.44
H526Y	1576	C>T	4	2	4	-1.38	1.2
H526L	1577	A>T	1	1	1	-1.49	-0.584

H526Q	1578	C>A	1	0	1	-0.386	0.3
H526Q	1578	C>G	0	0	1	-0.386	0.3
R529H	1586	C>T	0	2	1	2	2.22
R529L	1586	C>A	1	3	1	-0.124	-1.42
S531F	1592	C>T	0	7	3	0.592	0.779
S531Y	1592	C>A	0	1	0	1.34	6.32
A532P	1594	C>G	0	1	0	2.83	0.732
A532E	1595	C>A	0	0	2	-0.112	-0.729
L533H	1598	A>T	1	0	0	5	2.39
L533P	1598	A>G	2	3	5	2.35	2.18
L533R	1598	A>C	1	1	1	2.76	2.83
G534C	1600	C>A	2	3	1	8.42	6.2
G534D	1601	C>T	0	0	1	13.4	10.5
G534V	1601	C>A	0	5	3	13.5	8.26
G536V	1607	C>A	2	1	0	4.45	4.24
G537D	1610	C>T	0	2	1	21	10.2
T563P	1687	A>C	13	42	11	7.01	4.09
P564L	1691	C>T	3	5	4	1.65	1.8
P564R	1691	C>G	0	1	0	2.07	1.94
G570S	1708	C>T	1	0	0	11.6	9.47
G570A	1709	C>G	2	0	1	7.8	7.43
L571Q	1712	A>T	0	1	0	3.18	3.38
I572F	1714	A>T	6	12	2	-0.3	1.3
I572L	1714	A>C	10	19	14	-0.215	0.248
I572N	1715	A>T	6	3	4	0.561	1.52
I572S	1715	A>C	3	15	4	1.33	2.97
S574F	1721	C>T	1	2	1	8.17	-2.02
S574Y	1721	C>A	5	0	4	17.1	-1.43
12 bp del	1536	Deletion	1	0	0		
9 bp ins (dup)	1538	Insertion	0	0	1		

3 bp del	1539	Deletion	0	0	1		
3 bp del	1588	Deletion	0	0	1		
3 bp del	1594	Deletion	1	0	1		
6 bp del	1596	Deletion	0	0	1		
12 bp del	1601	Deletion	0	1	0		
9 bp del	1604	Deletion	0	1	0		
9 bp del	1606	Deletion	0	1	0		

Table S3: Mutational classes comparing the proportion of each SNP type in **Δ***nudJ* to the wildtype. Chi squared statistics for a binomial test of association between the genetic background and the proportion of observed mutants carrying the given mutational type are given with associated degrees of freedom and P values. P values corrected for the false discovery rate are given in the final column.

Mutational Class	Chi-Squared	DF	P	P (fdr corrected)
A>G	7.6500	2	0.00566	0.0227
A>C	9.6800	2	0.00187	0.0149
C>A	0.0846	2	0.77100	1.0000
C>T	0.4450	2	0.50500	0.8080
A>T	2.4000	2	0.12100	0.2420
C>G	0.0185	2	0.89200	1.0000
Deletion	4.3500	2	0.03710	0.0988
Insertion	0.0000	2	1.00000	1.0000
